# Distinct ATRX functions cooperate with 9-1-1 and CST complexes to safeguard replication and telomere integrity

**DOI:** 10.1038/s41594-026-01827-2

**Published:** 2026-06-30

**Authors:** Sandra Segura-Bayona, Marija Maric, Tohru Takaki, Zornitsa Manova, Tyler H. Stanage, Aurora I. Idilli, Shudong Li, Graeme Hewitt, Feras E. Machour, Rhona Millar, Marek Adamowicz, Ronnie Ren Jie Low, Phil Ruis, Benura Azeroglu, Todd Fallesen, Harshil Patel, Steven Howell, Panagiotis Kotsantis, Michael Howell, Simon J. Boulton

**Affiliations:** 1 DSB Repair Metabolism Laboratory, The Francis Crick Institute, London, UK; 2 Genome Stability Group, King’s College London, School of Cancer & Pharmaceutical Sciences, Comprehensive Cancer Centre, London, UK; 3 Crick Advanced Light Microscopy STP, The Francis Crick Institute, London, UK; 4 Bioinformatics and Biostatistics STP, The Francis Crick Institute, London, UK; 5 Proteomics STP, The Francis Crick Institute, London, UK; 6 Screening and Automated Science STP, The Francis Crick Institute, London, UK; 7 Present Address: Division of Biomedical and Life Sciences, Faculty of Health and Medicine, Lancaster University, Lancaster, UK

**Keywords:** Single-strand DNA breaks, Telomeres, Stalled forks, Stalled forks, Telomeres

## Abstract

Mutations in the *ATRX* chromatin remodeler confer a predisposition to a developmental genetic disorder and cancer, but how ATRX safeguards genome and telomere stability remains unresolved. Here, we uncover critical dependencies for the CTC1–STN1–TEN1 (CST) complex and RAD9A–HUS1–RAD1 (9-1-1) clamp in *ATRX*-deficient cells. *ATRX–CST* synthetic lethality manifests following accumulation of telomeric G-rich single-stranded DNA (ssDNA), which results in telomere loss and cell death. Conversely, we attribute *ATRX–9-1-1* synthetic lethality to genome-wide ssDNA lesions, which compromise DNA replication. We further show that ATRX suppresses DNA damage during replication stress by counteracting the activity of the FAM111A protease. We demonstrate that roles of ATRX in telomere maintenance and replication are genetically separable, requiring its ATPase activity and PIP-box, respectively. We also show that such roles protecting genome stability are largely independent of the ATRX–DAXX interaction. Collectively, our data show that functions of ATRX in suppressing toxic ssDNA lesions are context-dependent and are key to global DNA replication and telomere integrity.

## Abstract (web summary)

Segura-Bayona et al. uncover genetically separable ATRX functions that independently safeguard telomeres and genome replication, revealing how CST and 9-1-1 pathways suppress accumulation of toxic single-stranded DNA and preserve chromosome integrity.

## Main

Endogenous DNA damage and mutations in DNA-repair genes are associated with developmental syndromes and predisposition to cancer. One source of endogenous damage is replication stress, which can arise from DNA secondary structures, transcription-replication conflicts, abasic sites, ribonucleotide misincorporation or DNA-protein cross-links that present significant obstacles for DNA replication^1^. Replication stress can occur in distinct regions of the genome and poses a threat to genome and epigenome maintenance and, thus, cell proliferation and identity.

Cells have evolved replication-stress responses, including the checkpoint and DNA-damage-tolerance pathways. DNA-replication-fork stalling results in stretches of ssDNA, which are coated by RPA. RPA-coated ssDNA is a platform for recruiting and activating the ATR–ATRIP kinase complex^2,3^. ATR activation at stalled forks relies on two distinct pathways involving TOPBP1 and ETAA1 (refs. ^4–6^). TOPBP1-mediated ATR activation requires the heterotrimeric clamp complex 9-1-1, which is loaded onto 5′-ssDNA–double-stranded DNA (dsDNA) junctions by RAD17–RFC^4^. ETAA1-mediated ATR activation involves binding RPA–ssDNA directly^5,6^. ATR kinase signaling, resulting in CHK1 phosphorylation^7,8^, orchestrates the S/G2 checkpoint, suppressing origin firing and promoting fork stability and restart^9^.

Telomeres are intrinsically difficult to replicate and are a source of endogenous replication stress owing to their G-rich nature and propensity to form secondary structures^10^. To aid with resolution of telomeric structures, different factors cooperate to prevent fork stalling. The CTC1–STN1–TEN1 (CST) complex binds G-rich telomeric ssDNA^11^, ensures timely disengagement of telomerase from telomeres^12,13^ and recruits DNA-polymerase-α–primase to perform C-strand fill-in synthesis^14–16^. CST also plays a role in genome-wide DNA replication by stabilizing stalled forks under stress and promoting their restart^17–22^, as well as in double-strand break (DSB) repair by counteracting end resection^23^.

Germline mutations in the *ATRX* gene are associated with a rare genetic disorder known as ATR-X syndrome, characterized by intellectual disability and developmental abnormalities^24^. *ATRX* somatic mutations are also prevalent in specific types of tumors (detected in ~20% of sarcomas, ~40% of certain brain tumors and ~15% of pancreatic neuroendocrine tumors)^25,26^, making it one of the most mutated genes in cancer. ATRX, a chromatin remodeler of the SNF2 superfamily, forms a complex with the histone H3.3 chaperone DAXX^27–29^, which facilitates histone H3.3 deposition at repetitive heterochromatic genomic regions, many of which are enriched in putative G-quadruplex (G4)–DNA forming sequences, including telomeres^30–34^. ATRX–DAXX also deposits H3.3 at euchromatic regulatory elements, such as CpG islands^35^, and suppresses spurious transcription and replication stress in heterochromatin^36–39^. To date, the reported roles of the ATRX–DAXX complex as an H3.3 chaperone during metabolism of G4-DNA^34^ and during homologous recombination^40^ require ATRX’s PHD-containing ADD chromatin-binding domain, interaction with DAXX, interaction with PCNA (PIP-box) and its ATPase activity. However, the underlying molecular mechanisms through which ATRX safeguards genome and telomere stability remain to be elucidated.

Here, we uncover synthetic lethal genetic interactions between ATRX deficiency and two key ssDNA metabolism complexes, CST and 9-1-1, highlighting critical roles for ATRX in averting toxic ssDNA accumulation across the genome. Whereas the loss of *ATRX–CST* drives telomere instability and telomere loss following telomeric G-rich ssDNA accrual, the loss of *ATRX–9-1-1* leads to replication fork stalling and collapse that compromise genome-wide replication. We show that ATRX limits replication stress by preventing the engagement of the FAM111A protease with active replication forks. Unexpectedly, we demonstrate that the ability of ATRX to suppress accumulation of toxic ssDNA lesions at telomeres and genome-wide is independent of DAXX and HP1α binding, but requires its ATPase function and PCNA interaction motif, respectively. Our findings reveal genetically separable functions for ATRX in preventing the accumulation of toxic ssDNA lesions, which are essential for global DNA replication, telomere integrity and cell survival.

## ssDNA metabolism complexes protect cells from ATRX loss

**Fig. 1:**
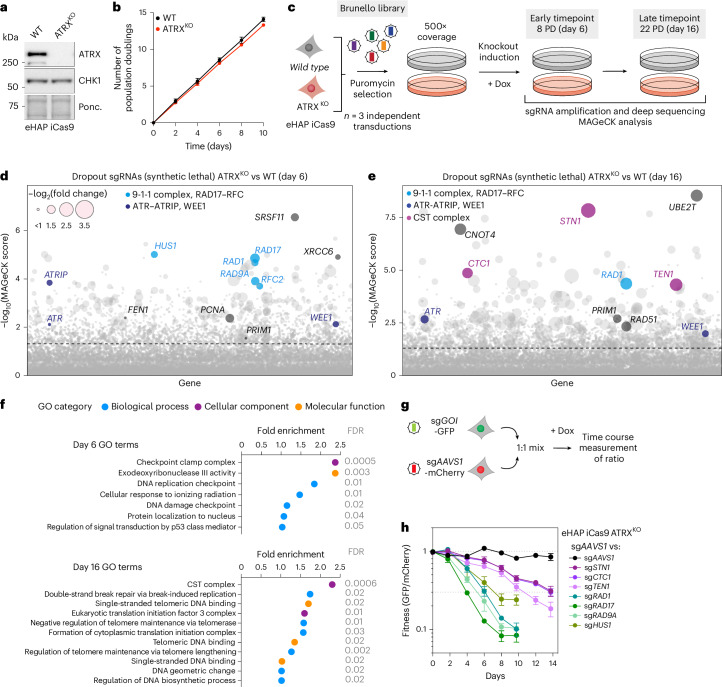
ssDNA metabolism complexes protect cells from ATRX loss. **a**, Immunoblot of whole cell extracts (WCEs) from eHAP iCas9 WT or *ATRX*-null cells, probed for ATRX. Total CHK1 and Ponceau (Ponc.) stain were used as loading controls. **b**, Growth curves in WT and *ATRX*-null eHAP iCas9 in unchallenged conditions. Data are shown as mean ± s.d. (*n* = 3 biological replicates). **c**, Schematic of the screening pipeline. **d**,**e**, Bubble plots illustrating the synthetic-lethal dropout hits identified by MAGeCK analysis at day 6 (**d**) and day 16 (**e**). The bubble size is proportional to log_2_(fold change). Genes are arranged on the *x* axis in alphabetical order. For full hit lists, see Supplementary Table 1. **f**, GO pathway enrichment of top 50 synthetic lethal genes (day 6) or top 100 synthetic lethal genes (day 16). FDR, false discovery rate. **g**, Schematic of two-colour competitive growth assays. **h**, Competitive growth assays in eHAP iCas9 *ATRX*-null cells transduced with virus expressing the indicated sgRNAs against genes of interest (GOI), as in **g**. Data are mean ± s.e.m. (*n* = 3 biological replicates). Source data

**Extended Data Fig. 1:**
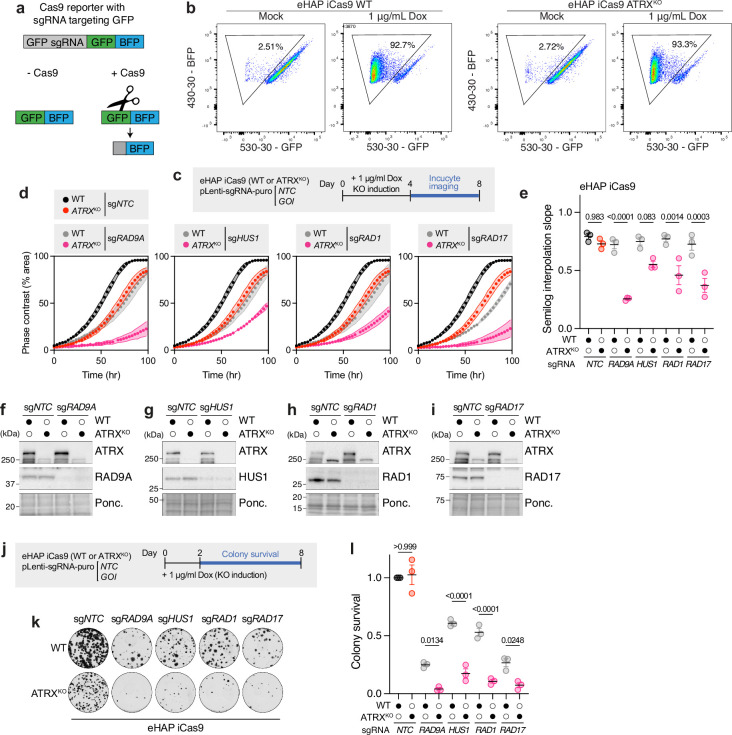
ssDNA metabolism complexes protect cells from ATRX loss. **(a)** Schematic of Cas9 GFP-BFP reporter assay. **(b)** WT and *ATRX*-null eHAP iCas9 clones were screened for Cas9 activity using GFP-BFP reporter after 96 hr Dox. BFP+ cells were gated and the loss of GFP expression was measured using FACS. Gates represent the percentage of ‘cut’ BFP + GFP- cells. **(c)** Schematic of pipeline for inducible knockout proliferation assays. **(d)** Proliferation curves for eHAP iCas9 WT or *ATRX*-null cells transduced with the indicated sgRNA seeded following 96 h Dox. Data for sg*NTC* are the same in the four graphs for reference. Data are mean ± SEM of technical triplicate, representative of n = 3 biological replicates. **(e)** Quantification of proliferation experiments as shown in (**d**). Semilog interpolation slopes of proliferation curves are shown. Data are mean ± SEM (n = 3 biological replicates, one-way ANOVA). **(f-i)** Immunoblot of WCE from WT or *ATRX*-null eHAP iCas9 cells transduced with *NTC*, *RAD9A* (**f**), *HUS1* (**g**), *RAD1* (**h**), *RAD17* (**i**) sgRNAs following 96 h Dox. Ponceau stain used as loading control. **(j)** Schematic of pipeline for inducible knockout colony survival assays. **(k)** Representative clonogenic survival assay in WT or *ATRX*-null eHAP iCas9 cells transduced with the indicated sgRNAs. Cells were seeded for colony formation in technical triplicate 2 days after adding Dox. Well diameter, 16 mm. **(l)** Quantification of clonogenic survival assays in cells treated as in (**k**). Data are mean ± SEM, normalised to WT sg*NTC* cells (n = 3 biological replicates, one-way ANOVA). Source data

To investigate the contribution of ATRX to genome stability maintenance, we sought to identify uncharacterized genetic interactions with *ATRX* through genome-scale CRISPR–Cas9 screens. To this end, we developed isogenic constitutive *ATRX* knockout (KO) in telomerase-positive chronic myelogenous leukemia-derived diploid eHAP cells expressing doxycycline-inducible Cas9 (iCas9), which retained proliferation capacity comparable to that of the parental (WT) cells (Fig. 1a,b and Extended Data Fig. 1a,b). To define the *ATRX* genetic interaction network in human cells, we transduced the isogenic parental and *ATRX*-null eHAP cells with the lentiviral Brunello single-guide (sg) RNA library (Fig. 1c). We analyzed dropout of single guide RNA (sgRNA) counts with the MAGeCK algorithm, comparing *ATRX*-null cells relative to the parental cells at two timepoints, day 6 (early) and day 16 (late) after the addition of doxycycline (Fig. 1c–e). Gene Ontology (GO) analyses of dropout genes highlighted enrichment of several complexes involved in DNA replication and telomere metabolism (Fig. 1f). Genes associated with the replication checkpoint clamp 9-1-1 and clamp loader RAD17–RFC subunits scored as the top dropout hits at the early time point (Fig. 1d,f). Additional members of the replication checkpoint pathway, such as *ATR*-*ATRIP*, the 9-1-1 interactor *RHNO1* (ref. ^41^) and the alternative ATR activator *ETAA1* (ref. ^6^) emerged as significant hits, albeit with lower rankings (Fig. 1d,e and Supplementary Table 1). By contrast, all CST-complex-subunit genes, including *CTC1*, *STN1* and *TEN1*, scored as top hits, but only at the late timepoint (Fig. 1e,f). We chose to explore the 9-1-1 checkpoint clamp and the CST complex in detail, the most significant complexes at both time points (Fig. 1f), given their links to the recognition and/or metabolism of ssDNA.

**Extended Data Fig. 2:**
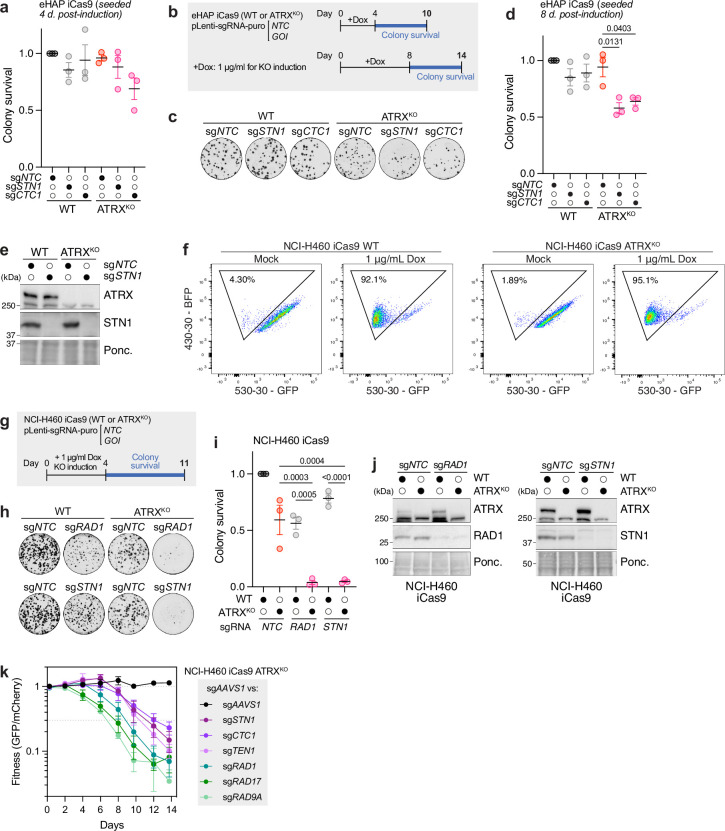
9-1-1 and CST complexes cooperate with ATRX to promote cell survival. **(a)** Quantification of clonogenic survival assays in WT or *ATRX*-null eHAP iCas9 cells transduced with *NTC*, *STN1* or *CTC1* sgRNA. Cells were seeded for colony formation in technical triplicate following 96 h Dox. Data are mean ± SEM, normalised to WT sg*NTC* cells (n = 3 biological replicates, one-way ANOVA). **(b)** Schematic of pipeline for inducible knockout colony survival assays. **(c)** Representative clonogenic survival assay in WT or *ATRX*-null eHAP iCas9 cells transduced with *NTC*, *STN1* or *CTC1* sgRNAs. Cells were seeded for colony formation in technical triplicate 8 days after adding Dox. Well diameter, 16 mm. **(d)** Quantification of clonogenic survival assays in cells treated as in (**c**). Data are mean ± SEM, normalised to WT sg*NTC* cells (n = 3 biological replicates, one-way ANOVA). **(e)** Immunoblot of WCE from WT or *ATRX*-null eHAP iCas9 cells transduced with *NTC* or *STN1* sgRNAs following 96 h Dox. Ponceau stain used as loading control (n = 3). **(f)** WT and *ATRX*-null NCI-H460 iCas9 clones were screened for Cas9 activity using the GFP-BFP reporter. **(g)** Schematic of pipeline for inducible knockout colony survival assays in NCI-H460 cells. **(h)** Representative clonogenic survival assay in WT or *ATRX*-null NCI-H460 iCas9 cells transduced with *NTC*, *RAD1* or *STN1* sgRNAs. Cells were seeded for colony formation in technical triplicate following 96 h Dox. Well diameter, 16 mm. **(i)** Quantification of clonogenic survival assays in cells treated as in (**h**). Data are mean ± SEM, normalised to WT sg*NTC* (n = 3 biological replicates, one-way ANOVA). **(j)** Immunoblot of WCE from WT or *ATRX*-null NCI-H460 iCas9 cells transduced with the indicated sgRNA following 96 h Dox. Ponceau stain used as loading control (n = 3). **(k)** Competitive growth assays in NCI-H460 iCas9 *ATRX*-null cells transduced with virus expressing the indicated sgRNAs against genes of interest (GOI) as in Fig. 1g. Data are mean ± SEM (n = 3 biological replicates). Source data

To further validate the genetic interaction between *ATRX* and the 9-1-1 DNA-replication checkpoint clamp observed at the early time point, we carried out live-cell imaging 96 h after Cas9 induction (Extended Data Fig. 1c). Inducible knockout of genes encoding the 9-1-1 clamp subunits, *RAD9A*, *HUS1* and *RAD1*, and the gene encoding the alternative clamp loader subunit, *RAD17*, showed a synergistic growth defect in eHAP *ATRX*-null cells, whereas the wild type tolerated loss of 9-1-1 and RAD17 (9-1-1/RAD17) and achieved confluency (Extended Data Fig. 1d–i and Supplementary Table 2). The synergistic cell-killing interaction between *ATRX* and the 9-1-1/RAD17 was also corroborated in clonogenic survival assays (Extended Data Fig. 1j–l and Supplementary Table 2). To confirm the genetic dependency between *ATRX* and the CST complex observed at the late time point, we carried out clonogenic survival assays in which we inducibly ablated the CST-complex-subunit genes *STN1* and *CTC1* in parental or *ATRX*-null eHAP iCas9 cell lines. Loss of *CTC1* and *STN1* resulted in a non-significant reduction in clonogenic survival in combination with *ATRX* loss at day 10 after Cas9 induction (Extended Data Fig. 2a,b). However, a significant growth defect was observed when CST subunits were inducibly knocked out in *ATRX*-null cells for 14 days (Extended Data Fig. 2b–e and Supplementary Table 2). We next developed isogenic constitutive *ATRX*-null pairs in the non-small-cell lung cancer line NCI-H460, in which inducible knockout of *RAD1* and *STN1* corroborated the pronounced synergistic cell killing with *ATRX* loss in clonogenic survival (Extended Data Fig. 2f–j and Supplementary Table 2).

To determine whether the time dependency of the two ssDNA-metabolism complexes could reflect a dichotomy in the underlying biology, we performed competitive growth assays with time-resolved imaging in eHAP and NCI-H460 iCas9 *ATRX*-null cells, using sgRNAs targeting the safe-harbor locus *AAVS1* and *CTC1*, *STN1*, *TEN1*, *RAD9A*, *HUS1*, *RAD1* and *RAD17* (Fig. 1g). In unchallenged conditions and upon Cas9 induction, sgRNAs targeting 9-1-1/RAD17 caused a significant growth disadvantage in eHAP *ATRX*-null cells (ratio < 0.3 over those targeting *AAVS1*) between days 4 and 7, whereas sgRNAs targeting CST reached that level of growth inhibition only between days 11 and 14 (Fig. 1h). This time difference was less marked in NCI-H460 *ATRX*-null cells, but both complexes still exhibited distinct dropout timing, with sgRNAs targeting 9-1-1/RAD17 becoming depleted between days 6 and 9 and sgRNAs targeting CST on days 10 and 12 (Extended Data Fig. 2k). This raised the possibility that 9-1-1/RAD17 and CST complex activities protect against distinct aspects of ATRX deficiency.

## G-rich telomeric ssDNA is responsible for the *ATRX*–*CST* genetic interaction

**Fig. 2:**
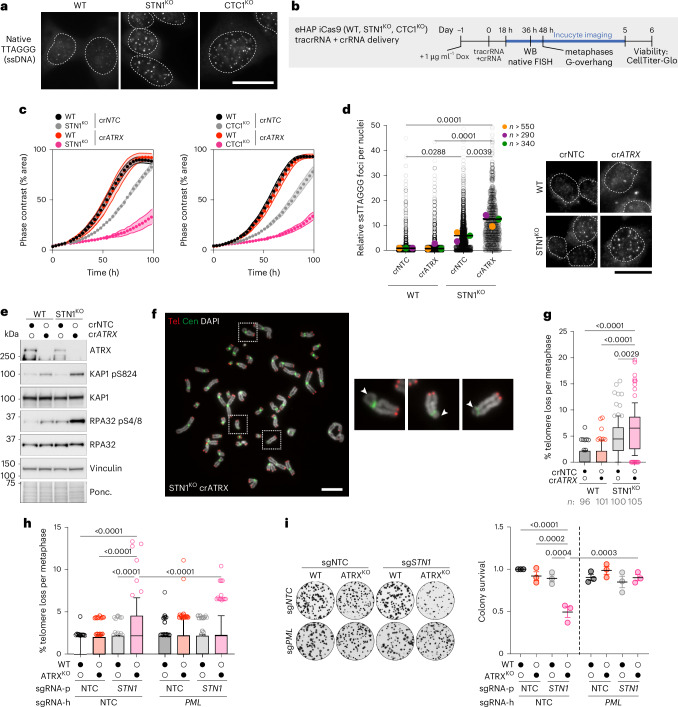
G-rich telomeric ssDNA is responsible for the *ATRX*–*CST* genetic interaction. **a**, Representative ssTTAGGG micrographs showing increased telomere G-rich ssDNA accumulation in the indicated cell lines (*n* = 3). Dotted line represents DAPI. Scale bar, 20 µm. **b**, Schematic of the experimental workflow for inducible knockout transfection and downstream assays. **c**, Proliferation curves for eHAP iCas9 WT, *STN1*-null or *CTC1*-null cells seeded 18 h after transfection with the indicated crRNA and tracrRNA in the presence of dox. Data are mean ± s.d. of six technical replicates, representative of *n* = 4 biological replicates. **d**, Left, quantification of ssTTAGGG foci in WT or *STN1*-null eHAP iCas9 cells 36 h after transfection with the indicated crRNA and tracrRNA in the presence of dox. Cells (*n*, each represented as a gray dot) and mean (colored dots) of *n* = 3 biological replicates (statistics on means, one-way ANOVA). Right, representative ssTTAGGG micrographs. Dotted line represents DAPI. Scale bar, 20 µm. **e**, Immunoblot of WCE from cells treated as described in **d**. Vinculin and Ponceau stain were used as loading controls. **f**, Representative images of telomere and centromere FISH in DAPI-stained metaphase spreads. Insets are magnifications of single chromosomes, with white arrowheads indicating signal-free ends. Scale bar, 10 µm. **g**, Quantification of the percentage of telomeres with no signal in WT or *STN1*-null eHAP iCas9 cells 48 h after transfection with the indicated crRNA and tracrRNA in the presence of dox. Box plots represent individual metaphases (***n***) with the interquartile range (IQR); the horizontal line indicates the median; and whiskers extend from the 10th to 90th percentile, pooled from 3 biological replicates (one-way ANOVA). **h**, Quantification of the percentage of telomeres with no signal in WT or *ATRX*-null eHAP iCas9 cells transduced with puromycin (p)-selectable non-targeting control sgRNA (sgNTC) or sg*STN1* and subsequently with hygromycin (h)-selectable sgNTC or sg*PML*, 10 days after dox treatment began. Box plots represent individual metaphases (*n* = 150) with the IQR; the horizontal line indicates the median; and the whiskers extend to the 10th to 90th percentile, pooled from 3 biological replicates (one-way ANOVA). **i**, Left, representative clonogenic survival assay in WT or *ATRX*-null eHAP iCas9 cells transduced with puromycin-selectable sgNTC or sg*STN1* and subsequently with hygromycin-selectable sgNTC or sg*PML*. Cells were seeded for colony formation in technical triplicate 8 days after dox treatment began. Well diameter, 16 mm. Right, quantification of clonogenic survival assays. Data are mean ± s.e.m., normalized to WT cells transduced with puromycin-selectable sgNTC and hygromycin-selectable sgNTC (*n* = 3 biological replicates, one-way ANOVA). Source data

**Extended Data Fig. 3:**
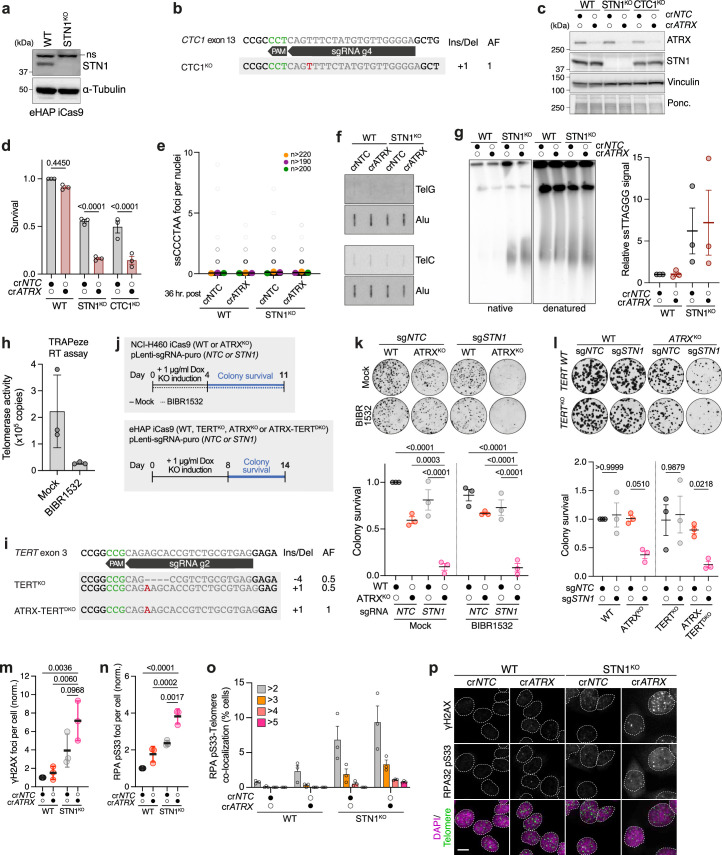
Molecular determinants of the ATRX:CST genetic interaction. **(a)** Immunoblot of WCE from eHAP iCas9 WT and *STN1*-null clone. Alpha-tubulin levels used as loading control. **(b)** Mutation analysis of eHAP iCas9 *CTC1*-null clone. Insertion/deletion size (Ins/Del) and allele frequency (AF) are shown. **(c)** Immunoblot of WCE from WT, *STN1*-null or *CTC1*-null eHAP iCas9 cells transfected with the indicated crRNA+tracrRNA. Vinculin and Ponceau stain used as loading controls. **(d)** eHAP iCas9 WT, *STN1*-null or *CTC1*-null cells were seeded 18 hours after crRNA+tracrRNA transfection in the presence of Dox. Cell viability was determined after 6 days using CellTiter-Glo. Data are mean ± SEM, normalised to WT cr*NTC* (n = 3 biological replicates, one-way ANOVA). **(e)** Quantification of ssCCCTAA foci in WT or *STN1*-null eHAP iCas9 cells 36 hours after crRNA+tracrRNA transfection in the presence of Dox. Cells (n, each represented as grey dot) and mean (coloured dots) of n = 3 biological replicates. **(f)** Representative G- and C-circle assay in eHAP iCas9 WT or *STN1*-null cells transfected as in (**e**). **(g)** Left: representative G-overhang analysis of telomeres fractionated by pulse-field gel electrophoresis. Single-stranded telomeric DNA was detected using a ^32^P-[AACCCT]_4_ probe under native conditions, followed by in situ denaturation and rehybridization to capture total telomeric DNA. Right: quantification of normalized overhang signals relative to WT cr*NTC*. Data are mean ± SEM (n = 3 biological replicates). **(h)** hTERT activity in NCI-H460 cell extracts ± 15 µM BIBR1532 measured by TRAPeze RT assay. Data are mean ± SD (n = 3 technical replicates). **(i)** Mutation analysis of eHAP iCas9 *TERT*-null clones, isolated in WT or ATRX-KO g3 background. Insertion/deletion size (Ins/Del) and allele frequency (AF) are shown. **(j)** Schematic of pipeline for inducible knockout colony survival assays shown in (**k-l**). **(k)** Top: Representative clonogenic survival assay in WT or *ATRX*-null NCI-H460 iCas9 cells expressing *NTC* or *STN1* sgRNAs ± 15 µM BIBR1532. Well diameter, 16 mm. Bottom: Quantification of clonogenic survival assays. Data are mean ± SEM, normalised to WT sg*NTC* mock (n = 3 biological replicates, one-way ANOVA). **(l)** Top: Representative clonogenic survival assay in WT, *ATRX*-null, *TERT*-null or *ATRX-TERT*-null eHAP iCas9 cells expressing *NTC* or *STN1* sgRNAs. Well diameter, 16 mm. Bottom: Quantification of clonogenic survival assays. Data are mean ± SEM, normalised to WT sg*NTC* (n = 3 biological replicates, RM one-way ANOVA). **(m-n)** Quantification of nuclear γH2AX (**m**) and RPA32 pS33 (**n**) foci in WT or *STN1*-null eHAP iCas9 cells 36 hours after crRNA+tracrRNA transfection. Data are mean ± SD, normalised to WT cr*NTC* cells (n = 3 biological replicates with ≥400 nuclei/replicate, one-way ANOVA). **(o)** Quantification of percentage of cells, treated as in (**m-n**), with RPA32 pS33-telomere colocalizations. Data are mean ± SEM (n = 3 biological replicates). **(p)** Representative micrographs of cells treated as in (**m-o**) stained with γH2AX, RPA32 pS33, denaturing telomeric PNA FISH and DAPI. Merged telomere and DAPI channels are shown. Scale bar = 10 um. Source data

Although CST has functions in genome-wide DNA replication beyond telomere maintenance^17–21^, potential fork-stalling events elsewhere are mitigated by dormant origin firing, whereas defects in telomere replication are likely to progressively accumulate over population doublings owing to infrequent origin activation in telomeres^10,42^. Given that the significant dropout of all CST-complex subunits in *ATRX*-null cells emerged only at the late time point (Fig. 1e,f,h), we hypothesized that stable knockout clones of CST would have high levels of telomeric ssDNA and therefore elicit the synthetic lethal phenotype earlier. To test this, we generated constitutive CST-KO cell lines, which are tolerated in eHAP cells (*STN1*-null and *CTC1*-null) (Extended Data Fig. 3a,b). As predicted^14–16^, *STN1*-null and *CTC1*-null cells accumulated G-rich telomeric ssDNA (Fig. 2a). IncuCyte imaging of *STN1*-null and *CTC1*-null cells that were transiently transfected with synthetic CRISPR RNA (crRNA) targeting *ATRX* in combination with unmodified transactivating crRNA (tracrRNA) following Cas9 expression showed rapid loss of viability at early time points, which prevented double-knockout (DKO) cells from achieving confluency (Fig. 2b,c, Extended Data Fig. 3c,d and Supplementary Table 2). These data suggest that the accumulation of G-rich telomeric ssDNA following loss of the CST complex places a critical dependence on ATRX.

Apart from the physiological G-overhang, telomeric ssDNA signal is associated with pathological under-replicated DNA, ssDNA gaps or persistent replication intermediates^43,44^. We therefore systematically analyzed single-stranded C-rich (ssCCCTAA) and G-rich (ssTTAGGG) telomeric DNA in constitutive *STN1*-null cells. As expected, *STN1*-null cells accumulated G-rich telomeric ssDNA foci (Fig. 2a,d) but not significant levels of C-rich telomeric ssDNA (Extended Data Fig. 3e). Following acute depletion of *ATRX* using a crRNA, the number of ssTTAGGG foci was significantly increased 36 h after transfection compared with the number in parental *STN1*-null cells (Fig. 2d). Telomeric ssDNA was not present in the form of G- or C-circles, unlike the by-products formed during alternative lengthening of telomeres (ALT) (Extended Data Fig. 3f), suggesting that the *ATRX–CST* genetic interaction cannot be explained by CST’s previously described role in promoting C-circles in ALT cells^45,46^.

We next set out to test whether ATRX is required for G-overhang maintenance. *STN1*-null cells exhibited a ~6.5-fold increase in G-overhang compared with the amount in parental eHAP cells (Extended Data Fig. 3g). After 48 h of transfection with *ATRX* crRNA, the G-overhang did not change significantly (Extended Data Fig. 3g). We reasoned that, in the absence of CST-mediated fill-in, if the hyper-extended G-overhang was responsible for the *ATRX–CST* genetic interaction, the loss of G-strand extension by telomerase would attenuate the lethality. To test this hypothesis, we abolished telomerase activity through chemical inhibition with BIBR1532 in NCI-H460 cells or by generation of full *TERT* knockouts in eHAP cells (Extended Data Fig. 3h–j). In accordance with the lack of a significant change in G-overhang length between the *STN1*-null and cr*ATRX*–*STN1* DKO cells, the *ATRX–STN1* synthetic genetic interaction was independent of telomerase activity (Extended Data Fig. 3j–l), suggesting that the synthetic lethality between *ATRX* and *STN1* is independent of G-rich telomere repeat addition by telomerase.

**Extended Data Fig. 4:**
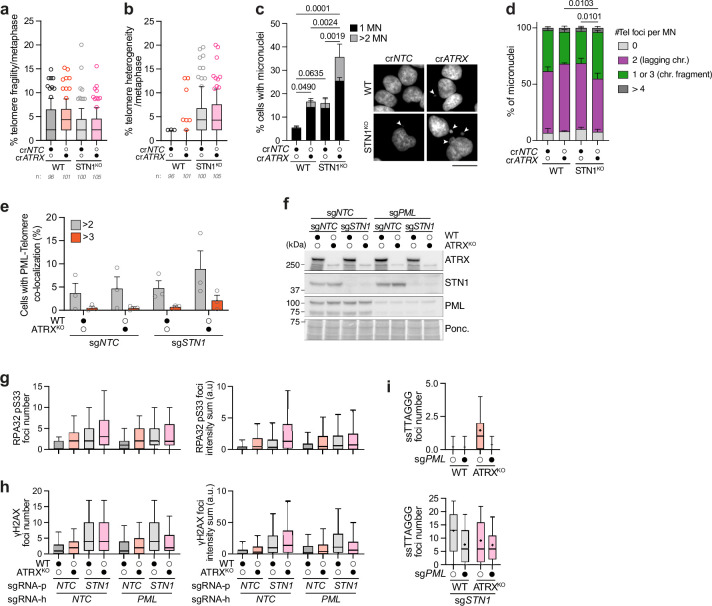
G-rich telomeric ssDNA is responsible for ATRX:CST genetic interaction. **(a-b)** Quantification of the percent of telomeres with fragility (**a**) or heterogeneity (**b**) in WT or *STN1*-null eHAP iCas9 cells 48 hours after transfecting with the indicated crRNA+tracrRNA in the presence of Dox. Box plots represent individual metaphases (**n**) with IQR, horizontal line indicates median, and whiskers extend to 10^th^ and 90^th^ percentile, pooled from 3 biological replicates. **(c)** Left: quantification of % micronuclei (MN)-positive cells in WT or *STN1*-null eHAP iCas9 cells 36 hours after transfecting with the indicated crRNA+tracrRNA in the presence of Dox. Data are mean ± SD of n = 3 biological replicates, each with more than 200 cells analysed (one-way ANOVA). Right: representative MN DAPI micrographs. Scale bar: 20 µm. **(d)** Quantification of percentage denaturing TelC-denaturing FISH signal present in micronuclei in cells as in (**c**). Data are mean ± SEM (n = 3 biological replicates, two-way ANOVA, effect on ‘1 or 3’ Tel foci shown). **(e)** WT or *ATRX*-null eHAP iCas9 cells were transduced with *NTC* or *STN1* sgRNAs. Quantification of percentage of cells with the indicated numbers of PML-telomere colocalizations, 10 days after starting Dox. Data are mean ± SEM, normalised to WT sg*NTC* cells (n = 3 biological replicates). **(f)** Immunoblot of WCE from WT or *ATRX*-null eHAP iCas9 cells transduced with *NTC* or *STN1* sgRNA-puromycin and subsequently with *NTC* or *PML* sgRNA-hygromycin, 8 days after starting Dox. Ponceau stain used as loading control. **(g-h)** Quantification of nuclear RPA32 pS33 (**g**) and γH2AX (**h**) foci number and intensity in cells as in (**f**), 10 days after starting Dox treatment. Box plots represent individual nuclei with IQR, horizontal line indicates median, and whiskers extend to 10^th^ and 90^th^ percentile, pooled from n = 2 biological replicates (>1300individual nuclei per condition). **(i)** Quantification of ssTTAGGG nuclear foci in cells as in (**f**), 10 days after starting Dox treatment. Box plots represent individual nuclei with IQR, horizontal line indicates median, and whiskers extend to 10^th^ and 90^th^ percentile, pooled from n = 2 biological replicates (>900 individual nuclei per condition). Source data

Notably, we found that *ATRX* deletion in *STN1*-null cells sparked a rapid accumulation of DNA-damage markers, including phosphorylation of RPA32 Ser4 and Ser8, RPA32 Ser33, KAP1 Ser824 and H2AX Ser139 (Fig. 2e and Extended Data Fig. 3m–p). To determine how telomere integrity was affected by the loss of *ATRX* in CST-deficient cells, we examined telomere fragility, heterogeneity and loss. Although acute *ATRX* loss did not affect telomere fragility or heterogeneity in *STN1*-null cells, it resulted in a significant increase in signal-free ends indicative of telomere loss, with an average of three telomeres completely devoid of detectable telomere FISH signal per metaphase (Fig. 2f,g and Extended Data Fig. 4a,b). Cells lacking both *STN1* and *ATRX* also had elevated levels of micronuclei when compared with those in the single-KO lines, with ~40% of cells having at least one micronucleus per primary nucleus; about one-third of these had two or more micronuclei (Extended Data Fig. 4c). Micronuclei in cr*ATRX–STN1* DKO cells exhibited increased numbers of telomere foci, indicating the presence of chromosomal DNA fragments compatible with telomere loss (Extended Data Fig. 4d).

Telomeres in *ATRX*-deficient cells exhibit increased interactions with PML bodies^47^. In normal human cells, PML bodies are associated with repair factors, such as BLM and MRE11, and with a few telomeres, preferentially when they are damaged^48–50^. In ALT cells, PML recruits the BLM complex^43^, which is responsible for generating single-stranded telomere intermediates^46,51^. Given the trend toward increased association of telomeres with PML bodies in sg*STN1*–*ATRX* DKO cells (Extended Data Fig. 4e), we set out to test whether PML loss could suppress the generation of telomeric G-rich ssDNA and induction of the damage response in eHAP sg*STN1*–*ATRX* DKO cells. The phosphorylation of RPA32 Ser33 and H2AX Ser139, observed in sg*STN1*–*ATRX* DKO cells, was reduced upon PML loss, implying that the protein is involved in DNA-damage accumulation in sg*STN1*–*ATRX* DKO cells (Extended Data Fig. 4f–h). Notably, G-rich telomeric ssDNA accumulated as a time-dependent consequence of chronic *ATRX* loss (Extended Data Fig. 4i), rather than an immediate effect detectable within two cell cycles of acute *ATRX* depletion (Fig. 2d). PML loss also completely abolished G-rich ssDNA in constitutive *ATRX*-null cells, whereas it only partially reduced G-rich ssDNA in inducible sg*STN1* cells, implicating PML in ssDNA generation upon *ATRX* loss and a subset of ssDNA generated in CST-deficient cells (Extended Data Fig. 4i). G-rich ssDNA levels in the sg*STN1*–sg*PML–ATRX* triple-KO cells were comparable to those in the sg*STN1*–sg*PML* DKO, suggesting that the signal present in sg*STN1*–sg*PML* DKO cells might represent G-overhangs. This supports the notion that they are independent of PML. Prolonged induction of sg*STN1* in *ATRX*-null cells also recapitulated the significant increase in signal-free ends indicative of telomere loss, which was rescued upon PML loss (Fig. 2h). The significant levels of signal-free ends observed in constitutive *STN1*-null cells (Fig. 2g) were not evident after 10 days of inducible *STN1* depletion (Fig. 2h), suggesting that a longer timeframe is required to elicit telomere loss in *STN1*-deficient cells. Finally, the synergistic growth defect of sg*STN1*–*ATRX* DKO cells was entirely rescued by PML loss (Fig. 2i). We conclude that ATRX ensures telomere integrity through inhibition of PML-dependent telomeric ssDNA formation, and this is crucial for cell survival when G-rich ssDNA accumulates upon prolonged CST absence.

## 9-1-1 cooperates with ATRX to suppress global replication fork collapse

**Fig. 3:**
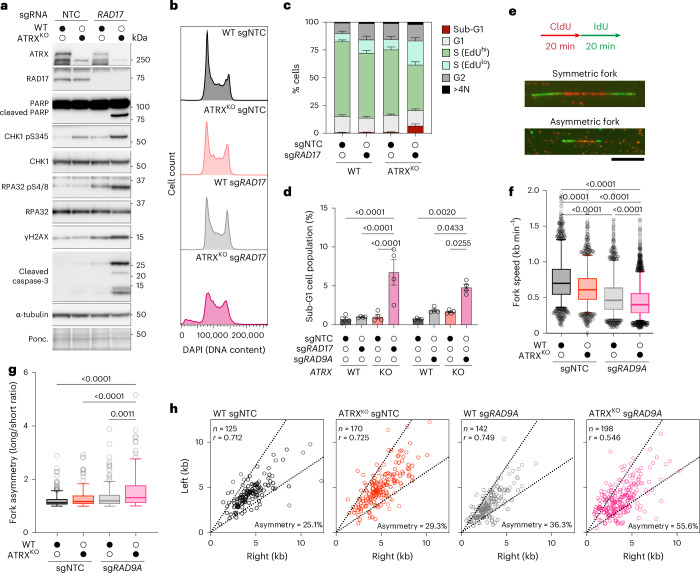
9-1-1 cooperates with ATRX to suppress global replication-fork collapse. **a**, Immunoblot of WCE from WT or *ATRX*-null eHAP iCas9 cells transduced with sgNTC or sg*RAD17* following 72 h dox. α-Tubulin and Ponceau stain were used as loading controls. pS4/8, phosphorylated at Ser4 and Ser8. **b**, Representative FACS DNA content analysis of eHAP iCas9 cells treated as in **a**. **c**, The percentage of cells in different cell-cycle stages. WT or *ATRX*-null eHAP iCas9 cells were transduced with sgNTC or sg*RAD17* following 72 h of dox treatment and pulsed with EdU. Data are mean ± s.e.m. (*n* = 4 biological replicates). **d**, Quantification of the sub-G1 cell population in the indicated cell lines following 72 h dox. Data are mean ± s.e.m. (*n* = 4 biological replicates, one-way ANOVA). **e**, Experimental setup and representative images of DNA fibers. Scale bar, 10 µm. **f**, Distribution of replication-fork speeds of DNA fibers, prepared as in **e**. Box plots represent individual fibers with the IQR, the horizontal line indicates the median and whiskers extend from the 5th to 95th percentile, pooled from *n* = 3 biological replicates (one-way ANOVA). **g**, Relative fork asymmetry. Fork asymmetry is expressed as the ratio of the longer track to the shorter one for each pair of sister replication forks. Box plots represent individual fibers with the IQR, the horizontal line indicates the median and whiskers extend to the furthest data points within 1.5 × IQR, pooled from *n* = 3 biological replicates (one-way ANOVA). **h**, Scatterplot of DNA fibers fork asymmetry prepared as in (**e**). Data are pooled from n = 3 biological replicates. One data point in *ATRX-*null sgNTC cells and two data points in *ATRX*-null sg*RAD9A* cells fall outside the plotted axis limits. n, number of bidirectional DNA fibers analysed, r, Pearson coefficient. Source data

**Extended Data Fig. 5:**
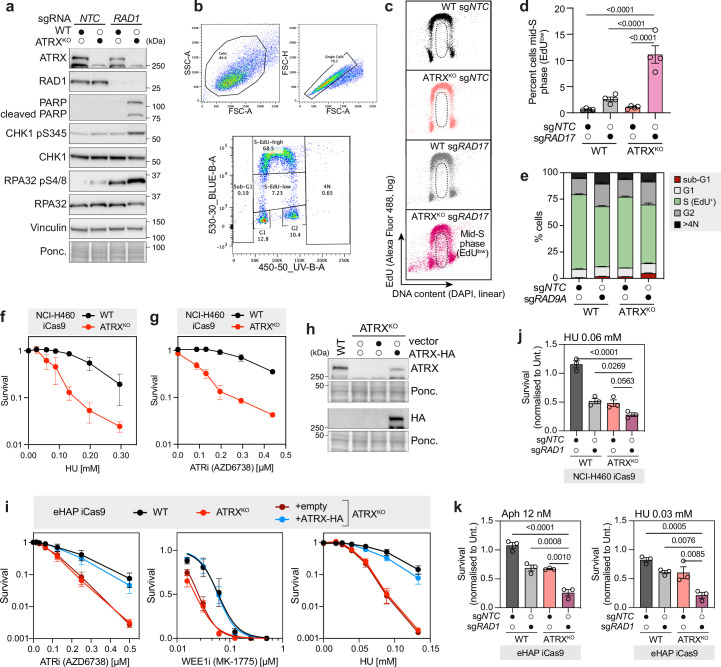
9-1-1 cooperates with ATRX to suppress global replication fork collapse. **(a)** Immunoblot of WCE from WT or *ATRX*-null eHAP iCas9 cells expressing *NTC* or *RAD1* sgRNA after 72 h Dox. Vinculin and Ponceau stain used as loading controls (n = 3 biological replicates). **(b)** Flow cytometry gating strategy for EdU/DNA analysis (forward scatter (FSC)/side scatter (SSC), doublet exclusion, EdU-AF488/DAPI with frequencies shown for all subpopulations). **(c)** Representative EdU/DNA content plots for WT or *ATRX*-null eHAP iCas9 cells expressing *NTC* or *RAD17* sgRNA after 72 h Dox. **(d)** Quantification of mid-S-phase (EdU-low) population as gated in (c). Data are mean ± SEM (n = 4 biological replicates, one-way ANOVA). **(e)** Cell cycle distribution in WT or *ATRX*-null eHAP iCas9 cells expressing *NTC* or *RAD9A* sgRNA after 72 h Dox and pulsed with EdU. Data are mean ± SEM (n = 4 biological replicates). **(f-g)** NCI-H460 iCas9 WT or *ATRX*-null cells were treated continuously for 6 days with the indicated doses of HU (**f**) and ATR inhibitor AZD6738 (**g**). Viability was determined using CellTiter-Glo. Data are mean ± SEM, normalised to untreated (n = 3 biological replicates). **(h)** Immunoblot of WCE from WT or *ATRX*-null eHAP iCas9 cells stably expressing HA-tagged ATRX cDNA. Ponceau stain used as loading control. **(i)** eHAP iCas9 cells as in (**h**) were treated continuously for 5 days with the indicated doses of AZD6738, MK-1775 or HU, and viability was determined using CellTiter-Glo. Data are mean ± SEM, normalised to untreated (n = 3 [ATRi and WEE1i] or n = 5 [HU] biological replicates). **(j)** NCI-H460 iCas9 WT or *ATRX*-null cells expressing *NTC* or *RAD1* sgRNA were treated continuously for 6 days with HU, viability was determined using CellTiter-Glo. Data are mean ± SD, normalised to untreated (n = 3 biological replicates, one-way ANOVA). **(k)** eHAP iCas9 WT or *ATRX*-null cells expressing *NTC* or *RAD1* sgRNA were treated continuously for 5 days with aphidicolin (Aph) or HU, viability was determined using CellTiter-Glo. Data are mean ± SD, normalised to untreated (n = 3 biological replicates, one-way ANOVA). Source data

To understand how the combined loss of ATRX and 9-1-1/RAD17 promotes cell death, we examined DNA-damage signaling in cells expressing NTC, *RAD1*- or *RAD17*-targeting sgRNAs 72 h after Cas9 activation. Loss of 9-1-1/RAD17 in *ATRX*-null cells resulted in activation of DNA damage signaling, including phosphorylation of CHK1 at Ser345, RPA32 at Ser4 and Ser8 and H2AX at Ser139 (Fig. 3a and Extended Data Fig. 5a). We assume that CHK1 phosphorylation in cells lacking 9-1-1 is mediated by ETAA1-dependent ATR activation (refs. ^5,6^) or cross-talk between PIKKs (ATM or DNA-PK), as reported previously^52,53^. Additionally, activation of apoptosis was evident from PARP1 or Caspase-3 cleavage in double mutants (Fig. 3a and Extended Data Fig. 5a). *ATRX*-null cells or ablation of 9-1-1/RAD17 in parental WT cells had only mild effects on the cell cycle, whereas ablation of 9-1-1/RAD17 in *ATRX*-null cells resulted in alterations in S-phase and an accumulation of a sub-G1 population, consistent with apoptosis (Fig. 3b–d and Extended Data Fig. 5b–e). Notably, in sg*RAD17*–*ATRX* DKO cells, ~10% of mid-S phase cells failed to incorporate the nucleotide analogue EdU, suggestive of compromised DNA replication (Fig. 3c and Extended Data Fig. 5c,d).

To examine DNA replication directly, we measured fork extension rates and symmetry of bidirectional forks using the DNA fiber assay. To this end, we sequentially pulse-labeled active replication forks with CldU and IdU nucleotide analogues and used immunofluorescence to label replication forks (Fig. 3e). *ATRX* or *9-1-1* single-knockout cells exhibited reduced replication fork-extension rates and a modest increase in fork asymmetry, which is suggestive of fork stalling and dormant origin activation. Combined loss of *ATRX* and *9-1-1* resulted in a further attenuation in fork speed when compared with parental and single KOs (Fig. 3f). Consistent with the reduced EdU incorporation (Fig. 3c and Extended Data Fig. 5c,d), >50% of newly activated replication origins exhibited fork asymmetry in sg*RAD9A*–*ATRX* DKO cells (Fig. 3g,h). *ATRX*-null cells showed heightened sensitivity to chemical ATR inhibition (ATRi) or G2/M checkpoint inhibition^54^ (WEE1i), as well as to DNA-replication perturbation following hydroxyurea (HU), in both eHAP and NCI-H460 cells (Extended Data Fig. 5f–i). Concomitant loss of *ATRX* and *9-1-1* resulted in additive sensitivity to DNA replication perturbation by HU or aphidicolin (Extended Data Fig. 5j,k). Taken together, these findings suggest that ATRX is required to prevent the accumulation of endogenous replication-borne toxic lesions and the observed synthetic lethality with 9-1-1/RAD17 stems from global replication fork stalling and/or collapse, which triggers apoptosis.

## ATRX restrains FAM111A chromatin engagement to protect replication forks from degradation

**Fig. 4:**
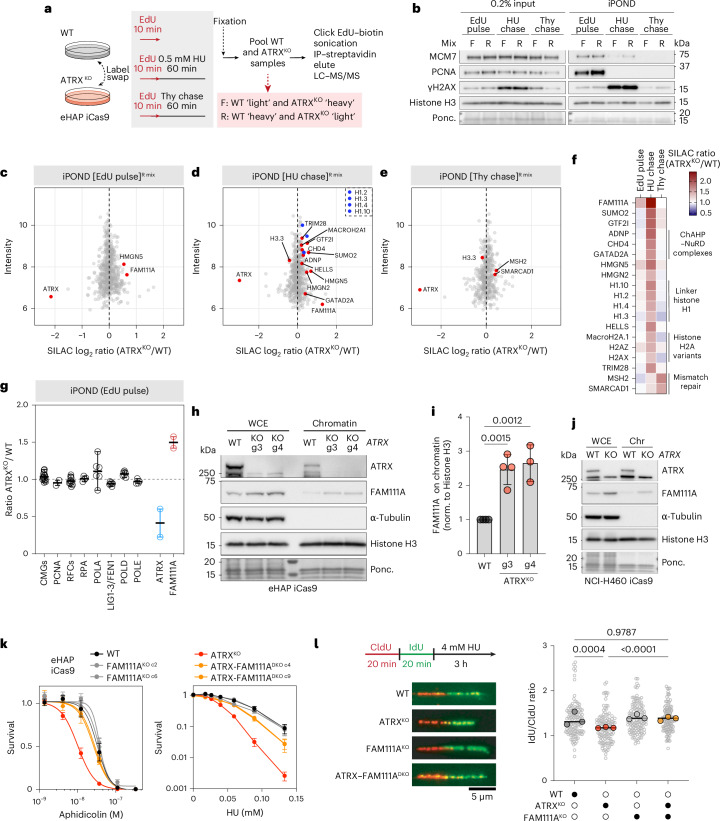
ATRX restrains FAM111A chromatin engagement to protect replication forks from degradation. **a**, Schematic of iPOND–SILAC–MS strategy for proteomics analysis of active and stalled replication forks, and post-replicative chromatin. The ‘heavy’ and ‘light’ isotope label was swapped during SILAC labeling for the two biological replicates, resulting in pooled forward (F) and reverse (R) mixes before iPOND purification. **b**, Immunoblot of input and iPOND pull-downs. MCM7 and PCNA are controls for active forks and γH2AX is a control for damaged forks. Histone H3 and Ponceau stain are used as loading controls (*n* = 2). **c–e**, Volcano plots illustrating protein intensity versus the log_2_ ratio of ATRX^KO^/WT SILAC for proteins identified by iPOND–MS in active replication forks (**c**), stalled replication forks (**d**) and post-replicative chromatin (**e**). The highlighted data points represent proteins that were significantly enriched or depleted in both biological replicates. **f**, Heat map of the ATRX^KO^/WT SILAC ratio of proteins identified as enriched in both biological replicates of iPOND–MS. **g**, Enrichment analysis showing the WT/ATRX^KO^ ratio of proteins identified through SILAC iPOND–MS at active replication forks. For CMGs (MCM2–MCM7 and GINS4), RFCs (RFC1–RFC5), RPA (RPA1 and RPA2), POLA (POLA1, PRIM1 and PRIM2), POLD (POLD1–POLD3), POLE–POLE3, LIG1-3 and FEN1 SILAC ratios include each detected subunit. Data are shown as the mean with the range (*n* = 2 biological replicates). **h**, Immunoblot of WCE versus chromatin from WT or *ATRX*-null asynchronous eHAP cells. α-Tubulin was used as a soluble fraction loading control. Histone H3 was used as a chromatin fraction loading control. **i**, Quantification of FAM111A chromatin loading as in **h** relative to levels of histone H3 chromatin. Data are mean ± s.d. (*n* = 4 biological replicates for WT and KO-g3, *n* = 3 biological replicates for KO-g4, one-way ANOVA). **j**, Immunoblot of WCE versus chromatin from WT or *ATRX*-null asynchronous NCI-H460 cells. α-Tubulin was used as a soluble fraction loading control. Histone H3 was used as a chromatin fraction loading control. **k**, eHAP cells were treated continuously for 5 days with the indicated doses of aphidicolin or HU, and viability was determined using CellTiter-Glo. Two independent KO clones for FAM111A-null (c2 and c6) and ATRX-FAM111A-null (c4 and c9) are shown. Data are mean ± s.e.m., normalized to untreated (*n* = 6 (aphidicolin) or *n* = 7 (HU) biological replicates). **l**, Quantification of fork degradation rate (IdU to CldU ratio) through DNA fiber experiment following 4 mM HU treatment in the indicated genotypes (*n* = 3 biological replicates; one-way ANOVA). Source data

**Extended Data Fig. 6:**
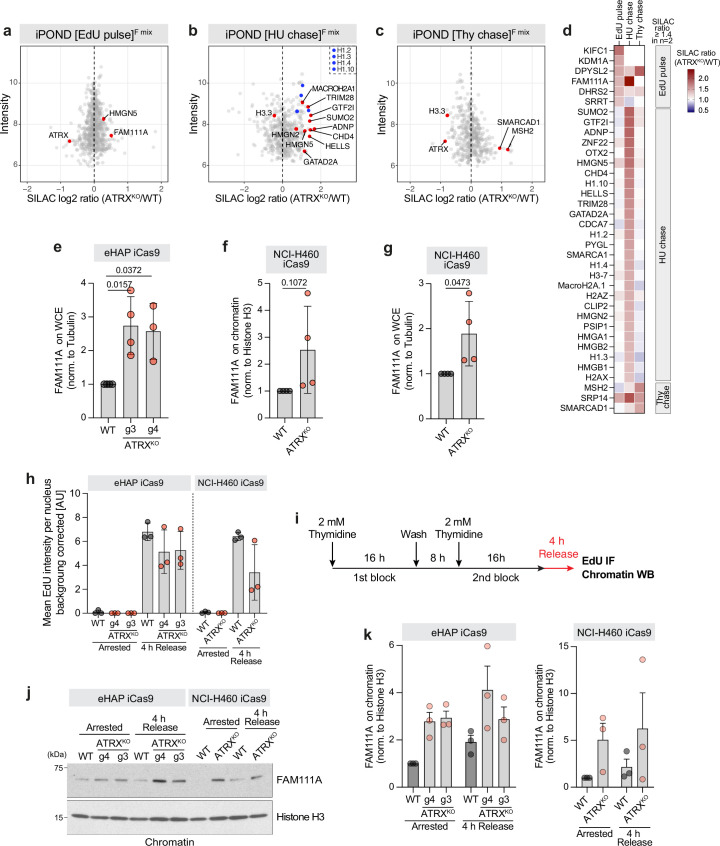
ATRX restrains FAM111A chromatin engagement. **(a-c)** Volcano plots of the second biological replicate, representing intensity versus the ATRX^KO^/WT SILAC log2 ratio of the iPOND-MS identified proteins in active replication forks (**a**), stalled replication forks (**b**) and post-replicative chromatin (**c**). Data points highlighted represent proteins enriched/depleted significantly changed in both biological replicates. **(d)** Heat map of ATRX^KO^/WT SILAC ratio of the 36 proteins identified as enriched in both biological replicates of iPOND-MS. **(e)** Quantification of FAM111A levels in WCE, relative to Alpha-Tubulin WCE levels. Data are mean ± SD (n = 4 biological replicates for WT and KO-g3, n = 3 biological replicates for KO-g4, one-way ANOVA). **(f)** Quantification of FAM111A chromatin loading in NCI-H460 cells relative to Histone H3 chromatin levels. Data are mean ± SD (n = 4 biological replicates, unpaired two-tailed t-test). **(g)** Quantification of FAM111A levels in WCE in NCI-H460 cells relative to Alpha-Tubulin WCE levels. Data are mean ± SD (n = 4 biological replicates, unpaired two-tailed t-test). **(h)** Mean EdU intensity per nucleus in eHAP or NCI-H460 cells, synchronised by double-thymidine block, either arrested at G1/S or released into S-phase. Data are mean ± SD (n = 3 biological replicates). **(i)** Schematic of pipeline for double-thymidine block cell synchronisation. **(j)** Immunoblot of chromatin fractions WT or *ATRX*-null synchronised eHAP and NCI-H460 cells as in (**g-h**). Histone H3 was used as a chromatin fraction loading control. **(k)** Quantification of FAM111A chromatin loading in synchronised eHAP or NCI-H460 cells relative to Histone H3 chromatin levels. Data are mean ± SEM, normalised to WT G1/S-arrested cells (n = 3 biological replicates). Source data

We hypothesized that ATRX may have previously unappreciated functions in the recruitment and/or turnover of specific factors at replication forks. To explore this possibility, we performed isolation of proteins on nascent DNA (iPOND) coupled with stable-isotope labeling with amino acids in cell culture (SILAC)-based quantitative mass spectrometry to compare protein occupancy at the fork between WT and *ATRX*-null cells^55^. We compared protein enrichments at active replication forks (EdU-pulse-labeled nascent chromatin) and at post-replicative chromatin that was allowed to mature during a 60-minute chase. Additionally, we included a chase sample in the presence of HU to enrich for stalled replication forks (Fig. 4a,b). ATRX was significantly enriched in WT eHAP cells in all conditions, including active forks, stalled forks and post-replicative chromatin (Fig. 4c–e and Supplementary Table 3). Although prior iPOND^56–58^ and nascent chromatin capture^59^ studies have reported damage-dependent and partially divergent findings, our data demonstrate that ATRX is associated with nascent chromatin under all conditions tested, including the unperturbed state. In the identified replication and chromatin-associated factors, 36 proteins emerged as being differentially enriched by a 1.4-fold average or more in *ATRX*-null cells in both biological replicates (Fig. 4c–f and Extended Data Fig. 6a–d). Notably, comparison between HU-treated *ATRX*-null and WT samples uncovered factors directly involved in chromatin condensation, remodeling and/or repair, including SUMO2; TRIM28; members of the ChAHP (CHD4, ADNP and HP1) and nucleosome remodeling and deacetylase (NuRD) repressor complexes; linker histone H1; and the histone H2A variants H2A.V, H2AX and macroH2A.1, including its histone chaperone and helicase HELLS (Fig. 4d,f and Extended Data Fig. 6b,d). These data suggested that nascent chromatin is most affected in the absence of ATRX upon severe fork stalling and replication stress.

The core replisome machinery, such as CMGs, PCNA, RFCs, RPA, DNA polymerases (POLD, POLA and POLE) and lagging-strand processing components (LIG1, LIG3 and FEN1), did not change remarkably upon *ATRX* loss at active replication forks (Fig. 4g). By contrast, FAM111A was one of the few proteins significantly enriched in *ATRX*-null cells at active replication forks in both unchallenged and HU-treated conditions (Fig. 4c,d,f,g). FAM111A is a PCNA-interacting protease that is mutated de novo in Kenny–Caffey syndrome type 2 and Gracile bone dysplasia, which interacts with active replication forks and exhibits specific ssDNA-binding activity in vitro^57,59–61^. Although its protease activity has been shown to protect against topoisomerase-induced DNA-protein cross-links (DPCs)^61^, its unrestrained function, best illustrated by ectopic overexpression of wild-type or the constitutively active R569H mutant, results in toxic ssDNA formation, replicative stress and cell death^62,63^. To validate that FAM111A differentially associates with newly synthesized DNA in *ATRX*-null cells, we first compared levels in the chromatin fraction. Consistent with iPOND–SILAC proteomics, two independent *ATRX*-null clones exhibited significant enrichment of FAM111A on chromatin (Fig. 4h,i). Quantification of both chromatin and whole-cell extract fractions revealed that this enrichment is accompanied by an increase in total cellular FAM111A levels (Fig. 4h and Extended Data Fig. 6e), indicating that the chromatin-associated increase may reflect, in part, elevated protein abundance. To assess whether this observation was restricted to a rapidly proliferating cell line, we extended these analyses to the slower-dividing NCI-H460 cell line. In asynchronous NCI-H460 cultures, *ATRX* loss was associated with increased total cellular and chromatin-associated FAM111A, although the latter did not reach statistical significance (Fig. 4j and Extended Data Fig. 6f,g). To further address potential cell-cycle specificity, we compared G1/S-arrested cells with matched populations released into S-phase (Extended Data Fig. 6h,i). Chromatin fractionation demonstrated that increased FAM111A association was detectable not only following S-phase entry, but also in G1/S-arrested *ATRX*-null cells (Extended Data Fig. 6i–k). These data indicate that enhanced chromatin engagement of FAM111A upon *ATRX* loss is not strictly confined to ongoing DNA replication.

**Extended Data Fig. 7:**
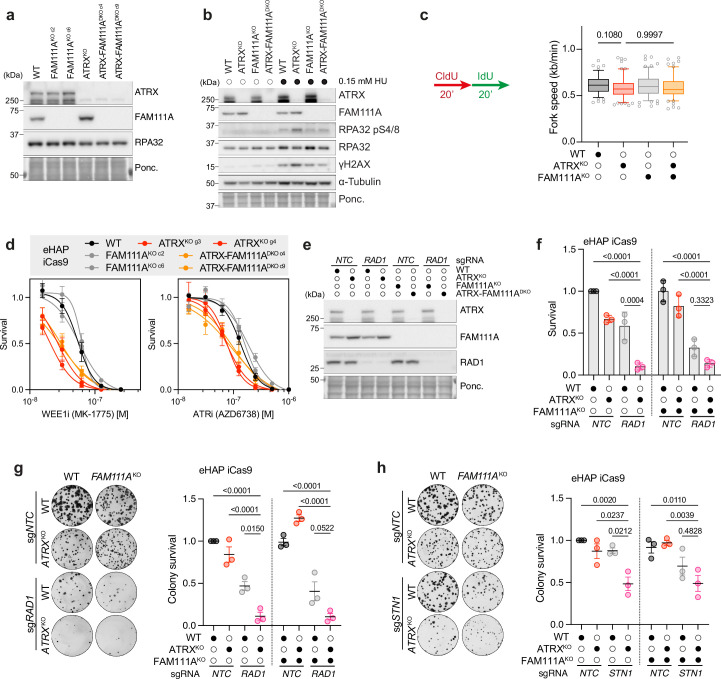
FAM111A promotes replication fork degradation in *ATRX*-null cells. **(a)** Immunoblot of WCE from eHAP iCas9 WT, *FAM111A*-null, *ATRX*-null and *ATRX-FAM111A*-null probed for ATRX and FAM111A. RPA32 levels and Ponceau stain used as loading controls. **(b)** Immunoblot of WCE from the indicated cell lines ± 0.15 mM HU for 24 hours. Alpha-tubulin and Ponceau stain used as loading controls (n = 3). **(c)** Left: Experimental setup for DNA fiber assay. Right: Distribution of replication fork speeds of DNA fibers in the indicated cell lines. Box plots represent individual fibres with IQR, horizontal line indicates median, and whiskers extend to 5^th^ and 95^th^ percentile, pooled from n = 3 biological replicates (two-way ANOVA). **(d)** eHAP cells were treated continuously for five days with WEE1 or ATR inhibitor at the indicated doses, and viability was determined using CellTiter-Glo. Data are mean ± SEM, normalised to untreated (n = 4 biological replicates). **(e)** Immunoblot of WCE from WT, *ATRX*-null, *FAM111A*-null and *ATRX-FAM111A*-null eHAP iCas9 cells transduced with *NTC* or *RAD1* sgRNA-puromycin, 3 days after starting Dox. Ponceau stain used as loading control. **(f)** WT, *ATRX*-null, *FAM111A*-null and *ATRX-FAM111A*-null eHAP iCas9 cells transduced with *NTC* or *RAD1* sgRNA-puromycin were seeded 3 days after starting Dox. Cell viability was determined after 6 days using CellTiter-Glo. Data are mean ± SD, normalised to WT sg*NTC* cells (n = 3 biological replicates, one-way ANOVA). **(g)** Left: Representative clonogenic survival assay in WT, *ATRX*-null, *FAM111A*-null and *ATRX-FAM111A*-null eHAP iCas9 cells transduced with *NTC* or *RAD1* sgRNAs. Cells were seeded for colony formation in technical triplicate 3 days after adding Dox. Well diameter, 16 mm. Right: Quantification of clonogenic survival assays. Data are mean ± SEM, normalised to WT sg*NTC* cells (n = 3 biological replicates, one-way ANOVA). **(h)** Left: Representative clonogenic survival assay in WT, *ATRX*-null, *FAM111A*-null and *ATRX-FAM111A*-null eHAP iCas9 cells transduced with *NTC* or *STN1* sgRNAs. Cells were seeded for colony formation in technical triplicate 8 days after adding Dox. Well diameter, 16 mm. Right: Quantification of clonogenic survival assays. Data are mean ± SEM, normalised to WT sg*NTC* cells (n = 3 biological replicates, one-way ANOVA). Source data

We next evaluated the survival of cells with *FAM111A* genetic inactivation in both parental and *ATRX*-null backgrounds (Extended Data Fig. 7a) in response to replication-inhibiting drugs. Loss of *FAM111A* did not affect survival after aphidicolin or HU treatment compared with WT cells (Fig. 4k). However, the combined loss of *FAM111A* and *ATRX* alleviated ATRX-dependent aphidicolin and HU hypersensitization (Fig. 4k). The HU resistance observed in *ATRX*-null eHAP cells upon *FAM111A* loss is consistent with previous studies in U2OS cells (ALT-positive *ATRX*-deficient)^62,63^. Of note, *FAM111A* knockout in *ATRX*-deficient cells attenuated HU-induced phosphorylation of RPA32 Ser4 and Ser8 and H2AX Ser139 (Extended Data Fig. 7b). We next examined whether FAM111A contributes to the reduced fork progression observed in *ATRX*-null cells (Fig. 3f). DNA fiber analysis showed that *ATRX–FAM111A* DKO cells did not display a significant change in fork speed compared with *ATRX*-null cells, indicating that FAM111A does not account for the basal fork slowing associated with *ATRX* deficiency (Extended Data Fig. 7c). Given the suppression of the sensitivity to replication perturbation and HU-induced damage signaling by FAM111A loss in *ATRX*-null cells (Fig. 4k and Extended Data Fig. 7b), we next focused on conditions of exogenous replication stress. *ATRX*-null cells exhibited a significant fork protection defect upon HU treatment, consistent with earlier reports^37^ (Fig. 4l). Notably, the loss of *FAM111A* fully restored fork stability, with nascent DNA degradation being no longer detectable in *ATRX–FAM111A* DKO cells (Fig. 4l). These results suggest that FAM111A drives pathological fork resection specifically in *ATRX*-null cells during replication stress.

Next, we asked whether *FAM111A* deficiency impacts the response of *ATRX*-deficient cells to checkpoint inhibition. In contrast to HU or aphidicolin sensitivity, deletion of *FAM111A* did not suppress the hypersensitivity of *ATRX*-null cells to ATR or WEE1 inhibitors (Extended Data Fig. 7d). Consistently, deleting *FAM111A* failed to rescue the synthetic lethality of untreated *ATRX–9-1-1* DKO cells (Extended Data Fig. 7e–g) or *ATRX–STN1* DKO cells (Extended Data Fig. 7h). By contrast, *ATRX–FAM111A* DKO rescues *ATRX*-null cell survival specifically under HU- or aphidicolin-induced replication stalling, but not under checkpoint inhibition or telomeric ssDNA accrual. This suggests that other factors, such as unrestrained origin firing, replication fork stabilization and repair, or DNA processing in PML bodies, contribute to the loss of viability seen in *ATRX–9-1-1* or *ATRX–STN1* DKO cells, respectively, which is independent of FAM111A.

## ATRX PIP-box suppresses genome-wide replicative stress

**Fig. 5:**
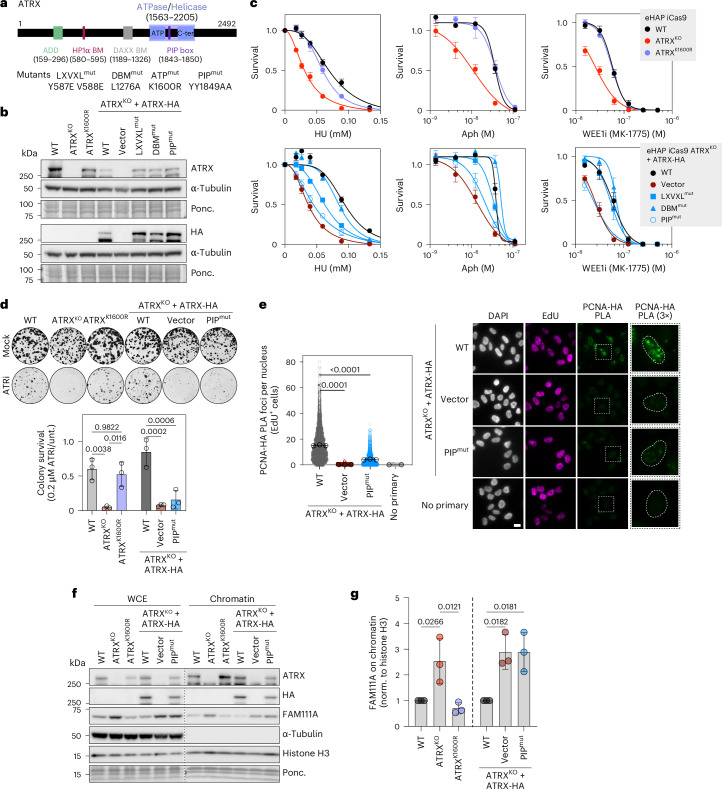
ATRX PIP-box suppresses genome-wide replicative stress. **a**, Schematic of full-length ATRX and mutants used to reconstitute *ATRX*-null cells. **b**, Immunoblot of WCE from cell lines, including K1600R knock-in and *ATRX*-null cells stably expressing HA-tagged *ATRX* cDNA mutants. α-Tubulin and Ponceau stain were used as loading controls. **c**, The indicated cell lines were treated continuously for 5 days with the indicated doses of HU, aphidicolin or the WEE1 inhibitor MK-1775, and viability was determined using CellTiter-Glo. Data are shown as mean ± s.e.m., normalized to untreated cells (*n* = 3 (HU and WEE1i), *n* = 4 (aphidicolin) biological replicates). **d**, Top, representative clonogenic survival micrographs of eHAP cells with or without continuous treatment with ATR inhibitor for 5 days. Well diameter, 16 mm. Bottom, quantification of clonogenic survival assays as indicated. Data are mean ± s.d., normalized to untreated (unt.) cells (*n* = 3 biological replicates, one-way ANOVA). **e**, Left, quantification of PLA foci number per nucleus in EdU^+^ *ATRX*-null eHAP cells stably expressing HA-tagged ATRX-WT, ATRX-PIP^mut^ or empty vector (*n* = 3 biological replicates, except the control condition with no primary antibodies where *n* = 2; one-way ANOVA). Right, representative images of PCNA–HA-ATRX signals in the indicated cell lines. Insets are ×3 magnifications of the indicated fields, where dotted line represents DAPI. Scale bar, 10 µm. **f**, Immunoblot of WCE versus chromatin from the indicated eHAP cells. α-Tubulin was used as a soluble fraction loading control. Histone H3 was used as a chromatin fraction loading control. **g**, Quantification of FAM111A chromatin loading as in **f**, relative to chromatin-bound histone H3 levels. Data are mean ± s.d. (*n* = 3 biological replicates, one-way ANOVA). Source data

**Extended Data Fig. 8:**
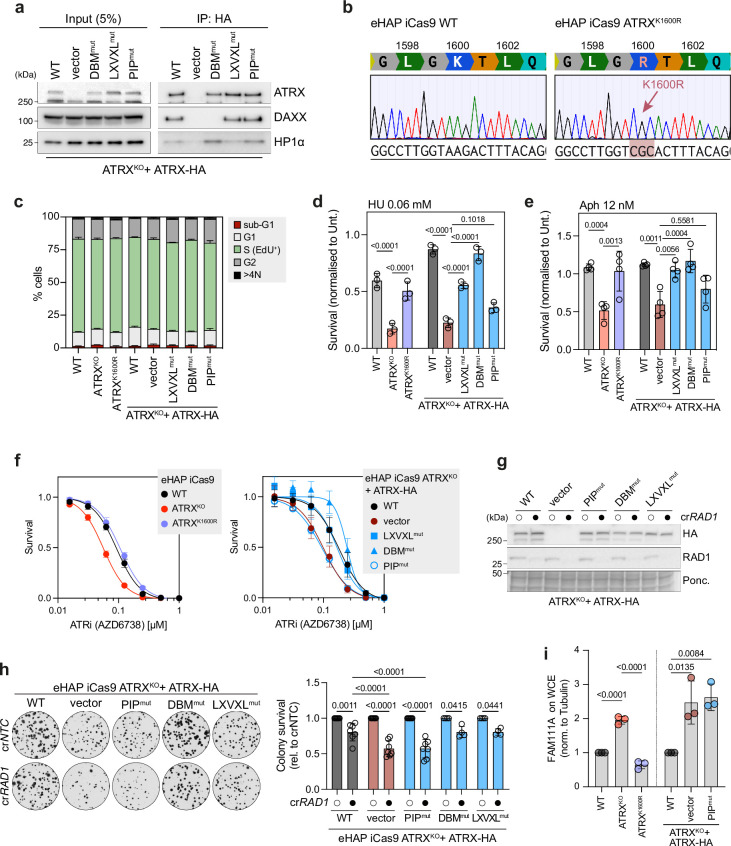
ATRX PIP-box suppresses genome-wide replicative stress. **(a)** Immunoblots of input lysates and HA immunoprecipitations from eHAP *ATRX*-null cells stably complemented with piggybac ATRX-HA constructs (n = 3). **(b)** ~ 800 bp PCR product flanking *ATRX* exon 17 was Sanger sequenced to confirm endogenous K1600R homozygous knockin. **(c)** Quantification of % of cells in different cell-cycle stages. The indicated eHAP iCas9 cell lines were pulsed with EdU for 15 min. Data are mean ± SEM (n = 3 biological replicates). **(d-e)** eHAP cells were treated continuously for five days with HU (**d**) or aphidicolin (**e**) at the indicated doses, and viability was determined using CellTiter-Glo. Data are mean ± SD, normalised to untreated of n = 3 (**d**) or n = 4 (**e**) biological replicates (one-way ANOVA). **(f)** The indicated cell lines were treated continuously for 5 days with the indicated doses of ATR inhibitor AZD6738, and viability was determined using CellTiter-Glo. Data are mean ± SEM, normalised to untreated cells (n = 3 biological replicates). **(g)** Immunoblot of WCE from the indicated eHAP iCas9 cell lines, 48 hours post-*NTC* or *RAD1* crRNA transfection. Ponceau stain used as loading control. **(h)** Left: Representative clonogenic survival assay in ATRX-HA complemented cells, seeded 48 hours post-*NTC* or *RAD1* crRNA transfection and Dox addition. Well diameter, 16 mm. Right: Quantification of colony survival determined 6 days post-seeding. Data are mean ± SD, normalised to cr*NTC* cells (n = 6 biological replicates for WT, empty and PIP-mut; n = 3 for DBM-mut and LXVXL-mut, one-way ANOVA). **(i)** Quantification of FAM111A levels in WCE, relative to Alpha-Tubulin WCE levels. Data are mean ± SD (n = 3 biological replicates, one-way ANOVA). Source data

ATRX is implicated in chromatin maintenance and DNA-repair processes, which rely on distinct protein-protein interactions (Fig. 5a). The amino-terminal ATRX–DNMT3–DNMT3L (ADD) domain binds to histone H3 trimethylated at K9 (H3K9me3) and unmodified H3K4 (H3K4me0), and the LXVXL HP1-interacting motif binds with HP1α^64,65^. ATRX binding to the histone chaperone DAXX requires an ~30-amino-acid motif and can be disrupted by mutation of Leu 1276 (ref. ^66^) (Extended Data Fig. 8a). A PCNA-interacting peptide (PIP)-box in ATRX located at residues 1843–1850 is required for irradiation-induced DNA-repair synthesis^40^. Finally, the SNF2-type ATPase and helicase enzymatic activity in the C-terminal domain facilitates histone exchanges at repetitive sequences and can be abolished by mutation of Lys 1600 (refs. ^67,68^).

To determine which domains in ATRX are required for protection against telomeric and global ssDNA lesions, we generated the indicated panel of point mutations (Fig. 5a). Complementation of *ATRX*-null cells using a C-terminal HA-tag stable expression system was achieved for WT and most mutants (Fig. 5b and Extended Data Fig. 5h). However, the ATPase-inactive ATRX K1600R substitution displayed severely reduced expression, which precluded its phenotypic analysis. To address this, we generated a homozygous knock-in cell line by direct Lys1600 substitution through Cas9-mediated homologous recombination at the endogenous *ATRX* locus in WT cells (Fig. 5b and Extended Data Fig. 8b). Cells expressing ATRX mutants were viable and did not show overt cell-cycle defects (Extended Data Fig. 8c).

Using this panel of ATRX mutants, we found that the genome-wide replication-stress sensitivity of *ATRX*-null cells was recapitulated with a PIP-box mutant (Fig. 5c and Extended Data Fig. 8d,e). Unexpectedly, ATRX ATPase activity and the ATRX–DAXX interaction were largely unnecessary for maintaining cell fitness during replication inhibition (Fig. 5c and Extended Data Fig. 8d,e). Similarly, the ATRX–HP1α interaction, mediated by the LXVXL motif, was only weakly required for survival in the presence of HU (Fig. 5c and Extended Data Fig. 8d,e).

We also observed a significant synergistic growth defect with ATR or WEE1 inhibition upon *ATRX* loss or with the PIP-mutant, but not with the ATPase inactive, DAXX-binding motif or LXVXL motif mutants (Fig. 5c,d and Extended Data Fig. 8f). Transient transfection with synthetic crRNA targeting *RAD1* following Cas9 expression showed that the ATRX PIP-box is required to protect cells against failure to activate the replication checkpoint (Extended Data Fig. 8g,h). Notably, ATRX interaction with PCNA at sites of DNA synthesis in unchallenged EdU^+^ cells was significantly reduced when lacking an intact PIP motif (Fig. 5e), and chromatin engagement of FAM111A was dependent on ATRX PIP-box but independent of ATRX ATPase activity (Fig. 5f,g and Extended Data Fig. 8i). Collectively, these data reveal that ATRX has distinct functions during DNA replication, independent of its roles in the ATRX–DAXX complex: the ATPase activity, DAXX-binding and HP1α-binding are dispensable for genome-wide replication, whereas the PCNA-interacting peptide is essential to suppress genome-wide replication stress.

## ATRX ATPase suppresses telomeric ssDNA independent of DAXX interaction

**Fig. 6:**
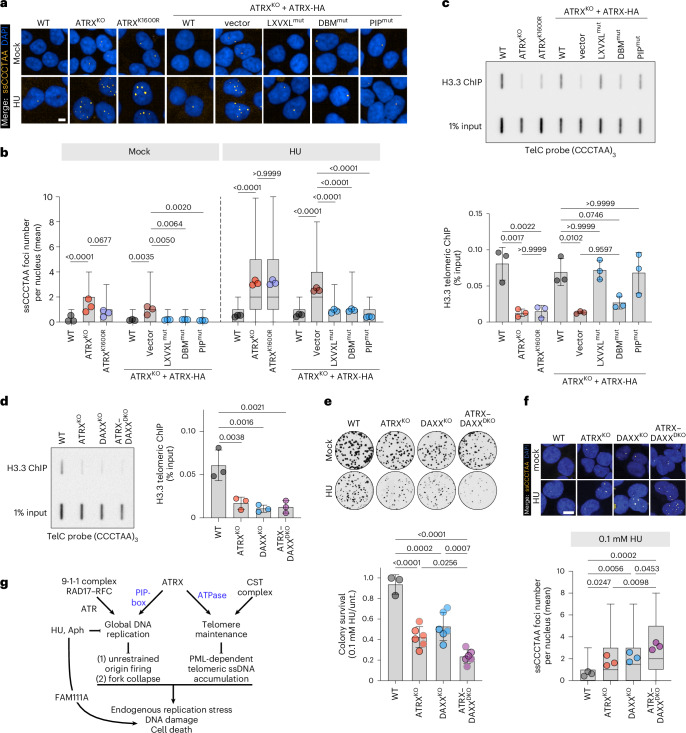
ATRX ATPase suppresses telomeric ssDNA independent of DAXX interaction. **a**, Representative ssCCCTAA micrographs showing telomere C-rich ssDNA accumulation in the indicated cell lines in the presence or absence of 0.15 mM HU. Scale bar, 10 µm. **b**, Quantification of ssCCCTAA foci per nucleus in cells, as in **a**. Box plots represent individual nuclei with the IQR, the horizontal line indicates the median and whiskers extend from the 5th to 95th percentiles. Colored dots are means of *n* = 3 biological replicates, with a minimum average of 600 nuclei per replicate (one-way ANOVA). **c**, Top, representative ChIP experiment using antibodies to histone H3.3. The immunoprecipitated DNA was blotted and probed with a telomere-specific probe. Bottom, quantification of H3.3 ChIP signal as a percentage of input at telomeres in the indicated cell lines. Data are mean ± s.d. (*n* = 3 biological replicates, one-way ANOVA). **d**, Left, representative histone H3.3 ChIP, with immunoprecipitated DNA blotted and probed with a telomere-specific probe. Right, quantification of H3.3 ChIP signal as a percentage of input at telomeres in the indicated cell lines. Data are shown as mean ± s.d. (*n* = 3 biological replicates, one-way ANOVA). **e**, Top, representative clonogenic survival micrographs of eHAP cells with or without continuous treatment with HU for 5 days. Well diameter, 16 mm. Bottom, quantification of clonogenic survival assays as indicated. Data are mean ± s.d., normalized to untreated cells (*n* = 3 biological replicates for WT, *n* = 6 for other genotypes which include two independent KO clones, one-way ANOVA). **f**, Top, representative ssCCCTAA micrographs showing telomere C-rich ssDNA accumulation in the indicated cell lines in the presence or absence of 0.1 mM HU. Scale bar, 10 µm. Bottom, quantification of ssCCCTAA foci per nucleus. Box plots represent individual nuclei with the IQR, the horizontal line indicates the median and whiskers extend from the 5th to 95th percentiles. Colored dots are means of *n* = 3 biological replicates (one-way ANOVA). **g**, Schematic model that illustrates how ATRX cooperates with 9-1-1 (through its PIP box) and CST (through its ATPase) complexes to suppress toxic ssDNA and maintain global DNA replication and telomere integrity, respectively. Source data

**Extended Data Fig. 9:**
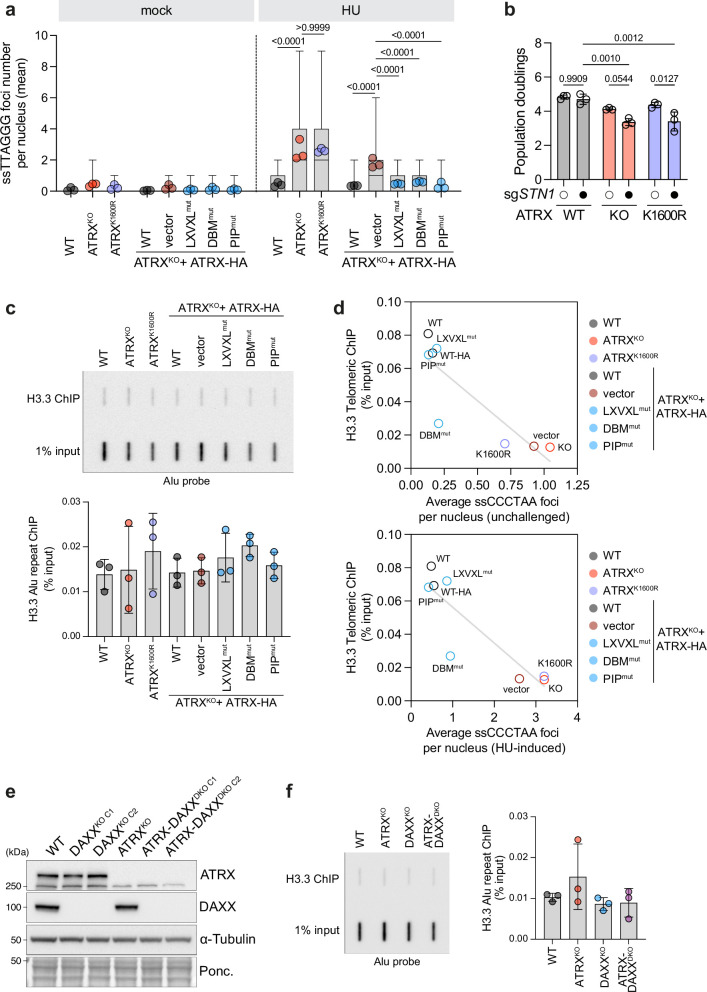
ATRX ATPase suppresses telomeric ssDNA independent of ATRX-DAXX interaction. **(a)** Quantification of ssTTAGGG foci per nucleus in the indicated cell lines in the presence or absence of 0.15 mM HU. Box plots represent individual nuclei with IQR, horizontal line indicates median, and whiskers extend to 5th and 95th percentile. Coloured dots are means of n = 3 biological replicates, with a minimum average of 600 nuclei per replicate (one-way ANOVA). **(b)** Quantification of population doublings of cells seeded 8 days post-transduction with sg*NTC* or sg*STN1* lenti-CRISPR-v2-hygromycin, determined 3 days post-seeding. Cells were previously selected with hygromycin and allowed to recover in fresh medium. Data are mean ± SD (n = 3 biological replicates, one-way ANOVA). **(c)** Top: representative ChIP experiment using antibodies raised against Histone H3.3. The immunoprecipitated DNA was blotted and probed with a probe specific for Alu repeats. Bottom: Quantification of H3.3 ChIP signal at Alu repeats as % of input, in the indicated cell lines. Data are mean ± SD (n = 3 biological replicates). **(d)** Correlation plots of average unchallenged H3.3 telomeric ChIP signal and ssCCCTAA foci per nucleus in unchallenged cells (top) or ssCCCTAA foci per nucleus in HU-treated cells (bottom) in the indicated cell lines. A linear regression is shown for reference with a grey line. **(e)** Immunoblot of WCE from eHAP iCas9 WT, *DAXX*-null, *ATRX*-null and *ATRX-DAXX*-null probed for ATRX and DAXX. Alpha-Tubulin levels and Ponceau stain used as loading controls. **(f)** Top: representative Histone H3.3 ChIP, with immunoprecipitated DNA blotted and probed with a probe specific for Alu repeats. Bottom: Quantification of H3.3 ChIP signal at Alu repeats as % of input, in the indicated cell lines. Data are mean ± SD (n = 3 biological replicates). Source data

To determine which activities of ATRX are required to protect against telomeric ssDNA, we assessed cells through native FISH using both telomeric G-rich and C-rich probes. The ATPase-inactivating K1600R substitution significantly impaired ATRX’s ability to suppress the generation of telomeric ssDNA (Fig. 6a,b and Extended Data Fig. 9a). This was most apparent after treating cells with a low dose of HU, which inhibits replication, but was also observed for C-rich ssDNA in unchallenged cells (Fig. 6a,b and Extended Data Fig. 9a). By contrast, ATPase-inactive ATRX-K1600R cells were not hypersensitive to HU or aphidicolin treatments (Fig. 5c and Extended Data Fig. 8d,e). The ATRX PIP-box motif, which protects against replication-stress sensitivity in eHAP cells, was dispensable for the generation of telomeric ssDNA (Figs. 5c and 6a,b and Extended Data Fig. 9a). Unexpectedly, the ATRX–DAXX interaction, mediated by ATRX Leu1276, and the ATRX–HP1α interaction, mediated by the LXVXL motif, were not required to suppress telomeric ssDNA (Fig. 6a,b and Extended Data Fig. 9a). Consistent with the ATRX ATPase suppressing telomeric ssDNA, ATRX ATPase-mutant cells displayed a growth defect in combination with *STN1* depletion (Extended Data Fig. 9b).

To further determine the chromatin-based mechanisms underlying ATRX-mediated telomere ssDNA protection, we performed chromatin immunoprecipitation (ChIP) for histone H3.3 at telomeric regions in our panel of ATRX mutant cell lines. As expected, *ATRX*-null cells showed reduced H3.3 occupancy at telomeres, consistent with ATRX’s role as an H3.3 chaperone within the ATRX-DAXX complex (Fig. 6c and Extended Data Fig. 9c). Cells expressing ATRX-K1600R or ATRX mutants defective for DAXX binding demonstrated a decrease in telomeric H3.3 occupancy comparable to *ATRX*-null cells, whereas the PIP-box motif and LXVXL-motif mutants phenocopied WT ATRX (Fig. 6c). These results indicate that the loss of telomeric H3.3 deposition can be uncoupled from the generation of telomeric ssDNA (Extended Data Fig. 9d), suggesting that ATPase-dependent functions of ATRX are the crucial determinant for suppressing ssDNA at telomeres, independent of its interaction with DAXX.

We next investigated the genetic relationship between ATRX and its binding partner DAXX. We established isogenic *DAXX*-null eHAP iCas9 cell lines in parental and *ATRX*-null backgrounds, generating single-KO and DKO cells (Extended Data Fig. 9e). ChIP analysis confirmed reduced H3.3 occupancy at telomeres in all mutants, consistent with epistasis and ATRX–DAXX’s role as a canonical H3.3 chaperone (Fig. 6d and Extended Data Fig. 9f). In viability assays, *DAXX* loss phenocopied the HU sensitivity observed in *ATRX*-null cells, whereas *ATRX–DAXX* DKO led to additive sensitivity to HU (Fig. 6e). Similarly, *DAXX* loss phenocopied the generation of HU-induced telomeric ssDNA species, whereas *ATRX*–*DAXX* DKO cells exhibited pronounced accumulation of HU-induced C-rich telomeric ssDNA, exceeding the levels observed in cells with single KO of either gene (Fig. 6f).

Collectively, these data reveal genetically separate functions for ATRX during DNA replication and telomere maintenance independent of its roles in the ATRX–DAXX complex: the ATPase activity of ATRX is essential for restricting telomeric ssDNA formation but is dispensable for genome-wide replication, whereas the PCNA-interacting motif is essential to suppress genome-wide replication stress but is dispensable for telomere maintenance. These results also suggest that although the ATRX–DAXX interaction contributes to H3.3 deposition at telomeres, it is not sufficient to suppress telomeric ssDNA, and that ATRX ATPase-dependent remodeling at telomeres has a central protective role independent of H3.3 loading.

## Discussion

*ATRX* is one of the most mutated genes in cancer, but how it safeguards genome and telomere stability remains unresolved. Here, we uncover context-dependent functions of ATRX in suppressing toxic ssDNA lesions and the compensatory pathways that *ATRX*-deficient cells critically rely on to prevent genome instability and cell death (Fig. 6g). Our data reveal previously unappreciated intrinsic functions of ATRX in genome maintenance that are independent of its interaction with DAXX and its canonical function as an H3.3 chaperone. Although DAXX has been shown to suppress R-loops, contributing to centromere stability and the transcriptional silencing of endogenous retroviruses independent of ATRX^66,69,70^, we show here that ATRX suppresses toxic ssDNA accumulation without relying on DAXX. We show that, contrary to current dogma, ATRX’s function in suppressing telomeric ssDNA requires its ATPase activity, but not its PIP-box motif, whereas its role during DNA replication requires its PIP-box motif, but not its ATPase activity.

We identify a synthetic lethal interaction between *ATRX–CST*, linking ATRX to the suppression of PML-dependent telomeric ssDNA formation (Fig. 2). Normally, the CST complex limits accumulation of G-rich ssDNA, and its loss results in progressive telomeric G-rich ssDNA accrual, with the magnitude of the phenotype correlating with the severity of the *ATRX–CST* synthetic lethality. It is possible that the lengthened G-overhang in CST deficient cells assembles an extended D-loop within the telomeric loop (t-loop), a structure where the 3′ ssDNA overhang invades double stranded telomeric repeats^71^. On the basis of our data, these D-loops could be ~6.5-fold longer than those in WT cells. ATRX could have a role in maintaining an extended D-loop, similar to its previously established involvement in homologous recombination (HR), downstream of RAD51 filament assembly and the action of RAD54 (ref. ^40^). However, the telomeric ATRX function we uncover depends exclusively on its ATPase activity, whereas ATRX’s role during HR^40^ requires both its ATPase and PIP-box activities, suggesting a distinct mechanism.

Instead, the most parsimonious explanation is that cells lacking CST might accumulate internal G-rich telomeric ssDNA (C-strand gaps)^72–74^, creating the exquisite dependency on ATRX ATPase-activity. We speculate that interstitial telomeric ssDNA could invade neighboring DNA creating loops that are subject to telomere trimming in PML nuclear bodies. Alternatively, fork stalling when encountering a gapped substrate could localize to PML bodies and result in one-ended DSBs, resulting in telomere loss. Previous reports demonstrated that CST complex activity, through POLA–PRIM, initiates the synthesis of complementary C-rich lagging strands in exposed gap or G4-DNA structures^74^. Our data suggest that telomeric ssDNA generation depends solely on ATRX ATPase function and is decoupled from the H3.3 chaperone complex, unlike the suppression of G4-DNA-induced stress, for which both ATPase and DAXX interaction are essential^34^. Together, these findings support a model in which CST and ATRX act in parallel to maintain telomere integrity, with ATRX averting catastrophic processing in PML bodies when CST is absent.

We define a synthetic lethal interaction between *ATRX* and *9-1-1*, which is recapitulated with an ATR inhibitor, implicating the ATRX PIP-box in protecting against endogenous lesions that critically rely on the replication checkpoint clamp 9-1-1. Loss of viability of *ATRX–9-1-1* DKO cells results from spontaneous replication-fork collapse, which culminates in apoptosis (Fig. 3). This is consistent with prior studies highlighting ATR inhibition as a potential vulnerability in *ATRX*-deficient ALT cancers^75^. Our data raise the possibility that the ATRX PIP-box function escorting the replisome is key to prevent 5′-ssDNA–dsDNA junctions that recruit 9-1-1. This is consistent with observations showing that the 9-1-1 complex protects against replication-fork stalling by shielding Okazaki fragments downstream of a replicative block from endonucleolytic attack^76^. Although ATRX ATPase activity and DAXX interaction are required for safeguarding against G4-DNA induced stress^34^, neither of these activities is necessary for protection against replication perturbation and S-phase checkpoint inhibition, which excludes G4-DNA as the source of ssDNA in this context. It will be important to determine whether disruption of additional ATRX motifs, including the ATRX–MRN (MRE11–RAD50–NBS1) interaction interface^77^, contributes to ssDNA accumulation or altered fork dynamics.

Our data show that ATRX prevents toxic lesions during DNA replication perturbation independent of ATRX ATPase activity, ATRX–DAXX and ATRX–HP1α interactions (Fig. 5). This establishes the existence of a non-canonical function of ATRX that escorts the replisome, independent of H3.3 deposition but dependent on its interaction with PCNA. Our experiments establish that ATRX counteracts chromatin engagement of FAM111A, particularly at active or stalled replication forks and independent of ATRX ATPase activity (Figs. 4 and 5). We further show that the ssDNA inducer FAM111A is responsible for damage accumulation and fork degradation at stalled replication forks in *ATRX*-deficient cells and is a key determinant of sensitivity to replication inhibition. Notably, FAM111A expression seems elevated in ATRX deficiency (Figs. 4h and 5f), which could reflect a potential link between its chromatin association and protein stability, as well as indirect transcriptional regulation^78^. FAM111A overexpression has been reported to undergo auto-cleavage, triggering its protease activity, which could increase ssDNA at forks through cleavage of replisome components, such as RFC1 (ref. ^62^). However, this was not observed in the presence of endogenous levels of FAM111A protease. To date, the targets of FAM111A protease are itself, nuclear pore-associated factors and TOP1 and PARP1 in cells treated with topoisomerase and PARP inhibitors^61–63,79^. The increased FAM111A loading at replication forks we observe in *ATRX*-null cells could still be in an auto-inhibited state from its own amino-terminal region^80^, and its release could be primed by exogenous damage. Although ATRX function could hinder FAM111A fork accessibility by limiting the presence of ssDNA, FAM111A could also act on endogenous ssDNA lesions, subsequently creating vulnerabilities in *ATRX*-deficient cells that require unique ssDNA metabolism complexes for their repair.

Finally, our data argue that the genome-maintenance functions of ATRX described here are largely independent of its canonical interaction with DAXX. Disruption of ATRX–DAXX binding does not phenocopy *ATRX* loss in the generation of telomeric ssDNA, replication-stress sensitivity or 9-1-1-dependent synthetic interaction, although it impairs H3.3 deposition at telomeres. This suggests that defective histone H3.3 loading per se is insufficient to explain the accumulation of telomeric ssDNA or the replication-associated lesions observed in *ATRX*-deficient cells. Instead, ATRX ATPase activity, but not DAXX binding, is required to suppress telomeric ssDNA, whereas the ATRX PIP-box mediates replication functions. Notably, although genetic ablation of *DAXX* phenocopies *ATRX* loss in replication-stress sensitivity and generation of telomeric ssDNA, combined *ATRX–DAXX* loss produces additive phenotypes, implying parallel, rather than epistatic, pathways. Collectively, these observations support a model in which ATRX mediates chromatin remodeling at telomeres and escorts replication forks, functions that are mechanistically distinct from its role as an H3.3 chaperone through DAXX.

## Methods

### Bacterial strains

Stbl3 *Escherichia coli* strain (genotype: F–mcrB mrr hsdS20(rB–, mB–) recA13 supE44 ara-14 galK2 lacY1 proA2 rpsL20(StrR) xyl-5 λ–leu mtl-1) was transformed with piggybac or lentiviral mammalian expression plasmids and grown in Luria Broth at 30 °C in the presence of ampicillin (100 µg ml^−1^).

### Cell lines

The human chronic myelogenous leukemia-derived cell line eHAP (haploid purchased from Horizon Discovery, cat. no. C669; stable diploid cells were used throughout) and the human lung cancer cell line NCI-H460 (purchased from ATCC, cat. no. HTB-177) were further modified by a lentiviral integration of a doxycycline-inducible Cas9 nuclease (iCas9) with an Edit-R inducible lentiviral Cas9 vector (Horizon Discovery)^81,82^. eHAP iCas9 and NCI-H460 iCas9 (parental WT cell lines) were derived as single clones from the pool of transduced cells after blasticidin selection (8 µg ml^−1^ for eHAP, 2.5 µg ml^−1^ for NCI-H460) and were chosen on the basis of a high cutting efficiency and negligible leakiness using a BFP–GFP reporter assay (Addgene cat. no. 67980). eHAP iCas9 parental cell line (and, where indicated, the *ATRX*-KO line) was used for the generation of *ATRX*, *STN1*, *CTC1, TERT*, *FAM111A* and *DAXX* full knockouts. eHAP cell lines were grown in Iscove′s modified Dulbecco′s medium (IMDM) with 10% Tet-free FBS and 1% penicillin–streptomycin. NCI-H460 iCas9 cells were used for the generation of *ATRX* full knockouts. NCI-H460 cell lines were grown in Roswell Park Memorial Institute (RPMI)-1640 medium with 10% Tet-free FBS and 1% penicillin–streptomycin. All cell lines were grown at 37 °C in 5% CO_2_. Cell lines were sourced, deposited and authenticated using STR profiling at the Cell Science facility at the Francis Crick Institute.

Although *ATRX*-null clones in eHAP cells and wild-type cells proliferate at similar rates in population-doubling assays, these same *ATRX*-null clones display mildly reduced growth in highly diluted conditions. NCI-H460 *ATRX*-null clones grow at a slower rate than wild-type cells. Similarly, *STN1*-null and *CTC1*-null clones in eHAP cells proliferate slower than do wild-type cells, and this effect is accentuated over cumulative passaging. All experimental sets were carried out in biological replicates, no more than two weeks apart.

### Plasmids

ATRX-HA was introduced into pDONR221 (Invitrogen) by amplification from IF-GFP-ATRX (Addgene cat. no. 45444) using primers attb1-ATRX-F: 5′-GGGGACAAGTTTGTACAAAAAAGCAGGCTTCATGACCGCTGAGCCCATG-3′ and attb2-ATRX-R: 5′-GGGGACCACTTTGTACAAGAAAGCTGGGTATCAAGCGTAATCTGGAACATCG-3′ (Sigma). Mutant variants were introduced by Q5 site-directed mutagenesis (NEB) in pDONR221, according to the manufacturer’s instructions (oligonucleotides, ordered from Sigma, are listed in Supplementary Table 5). Mammalian expression vectors were made in a piggyBac (PB) transposon delivery system PB-EF1α-DEST-IRES-puromycin. ATRX-HA was cloned into PB-EF1α-DEST-IRES-puromycin using the Gateway technology (Thermo Fisher Scientific), according to the manufacturer’s protocol. Expression constructs were introduced into eHAP iCas9 ATRX-KO cells by co-transfection with Super PiggyBac Transposase (PB200A-1, System Biosciences).

### Lentiviral transductions

To produce lentivirus, 900,000 293 FT cells in a six-well plate were transfected with packaging plasmids (566 ng of pLP1, 266 ng of pLP2, 370 ng of pLP/VSVG) along with 1 µg of lentiviral vector plasmid using 4 µl Lipofectamine 2000 (Thermo Fisher Scientific), per the manufacturer’s instructions. Medium was refreshed 18 h later. Virus-containing supernatant was collected 72 h post-transfection, cleared through a 0.45-µm filter, supplemented with 8 µg ml^−1^ polybrene (Sigma), and used for infection of target cells. Transductants were selected after one day of recovery. The following antibiotics were used for selection of transductants: puromycin (eHAP 0.4 µg ml^−1^; NCI-H460 1 µg ml^−1^; each for 2–3 days), hygromycin (eHAP 0.4 mg ml^−1^ for 3–4 days) and blasticidin (eHAP 8 µg ml^−1^, NCI-H460 2.5 µg ml^−1^; for 5 days).

### Generation of CRISPR full knockout cell lines

Knockouts of *ATRX*, *FAM111A* and *DAXX* in eHAP iCas9 and NCI-H460 iCas9 cells were made by transfection of the cell line with synthetic tracrRNA or crRNA using Lipofectamine RNAiMAX (Thermo Fisher Scientific). *ATRX*, *FAM111A* and *DAXX* targeting sequences are listed in Supplementary Table 4. After transfection, cells were incubated with 1 µg ml^−1^ doxycycline to induce Cas9 expression for 72 h, then cells were seeded in 96-well plates to derive single-cell clones. Knockouts of *STN1*, *CTC1* and *TERT* in eHAP iCas9 cells were made by transient transfection of the cell line with lenti-sgRNA-puromycin or lenti-sgRNA-hygromycin *(*Addgene nos. 104990 and 104991); targeting sequences are listed in Supplementary Table 4. Upon transfection, cells were incubated with 1 µg ml^−1^ doxycycline to induce Cas9 expression for 72 h, and were selected for 48 h in puromycin at a concentration of 0.4 µg ml^−1^ after 24 h of transfection. Then, cells were seeded in 96-well plates to derive single-cell clones. All stable knockout clones were validated by immunoblotting, sequencing the genomic DNA for the presence of a nonsense or frameshift mutation and screened for iCas9 cutting efficiency (with the BFP–GFP reporter assay).

### Generation of Dox-inducible Cas9-knockout cell lines

Inducible CRISPR-knockout cell lines were generated by transducing iCas9 cells with lentivirus produced from the lenti–sgRNA–puro or lenti–sgRNA–hygro constructs (Addgene nos. 104990 and 104991); target sequences of sgRNAs are listed in Supplementary Table 4. Inducible knockout of target proteins was confirmed in the pooled cell line by immunoblotting following treatment with 1 µg ml^−1^ doxycycline for 96 h, or, when antibodies were unavailable, genomic DNA sequencing of the pooled edited population.

### Generation of endogenous knock-in cell lines

sgRNAs targeting the exon of interest with high on-target and low off-target scores were designed using Benchling and cloned into the pGL3-U6-sgRNA-PGK-puromycin plasmid (Addgene cat. no. 51133). A homology-directed repair (HDR) DNA template containing the mutation of interest surrounded by 25-bp homology arms (Supplementary Table 5) cloned into the pUC19 vector was ordered from Genewiz. Then, 250,000 eHAP iCas9 cells in a 6-well plate were transfected with 4 µg sgRNA plasmid and 2 µg HDR plasmid using Lipofectamine 2000 (Thermo Fisher Scientific). At 35 h post-transfection, cells were selected with 0.4 µg ml^−1^ puromycin for 48 h, and 1 µg ml^−1^ doxycycline, 1 µM ART558 and 1 µM DNA-PKcs inhibitor were maintained in the medium during transfection, selection and expansion of the cells for a total of 5 days to increase HDR rates. Single-cell clones were isolated through serial dilution in 96-well plates. HDR was confirmed through Sanger sequencing of the locus of interest. Selected clones were expanded and further validated for protein expression levels using immunoblotting.

### CRISPR–Cas9 screening

Whole-genome CRISPR–Cas9 dropout and enrichment screens were performed as described^83^. eHAP iCas9 wild-type or ATRX^KO^ cells were transduced with the lentiviral Brunello library (Addgene cat. no. 73179-LV, sgRNA only vector). One hundred million cells were transduced to achieve a multiplicity of infection (MOI) of 0.4 in three biologically independent transductions, selecting for a coverage of 500 cells per sgRNA. Transduced cells were selected with puromycin (0.4 µg ml^−1^) for 48 h, after which Cas9 expression was induced with doxycycline (1 µg ml^−1^). Cells were subcultured in doxycycline for the initial 6 days and were later subcultured without doxycycline, with representation of 40 million cells (theoretical library coverage of 500 cells per sgRNA) maintained at every step by passaging every two days. Cells were collected both at an early time point (day 6 after Dox) and a late time point (day 16 after Dox), because identifying different sets of genes for which mechanisms of lethality may differ in timings. Samples for sequencing were collected by washing 60 million cells in PBS, freezing cell pellets and storing them at −80 °C.

Genomic DNA was isolated with PureLink Genomic DNA Mini Kit (Thermo Fisher Scientific). The quantity of genomic DNA was measured by Nanodrop and Qubit (Thermo Fisher Scientific). From each sample, 200 µg of genomic DNA was then used for library preparation, with one-step amplification of genome-integrated sgRNAs using P5 mix and P7 barcoded oligonucleotides (Sigma) in a PCR reaction with Ex Taq polymerase (TaKaRa) (Supplementary Table 5). PCR products were purified through the agarose gel extraction method using QIAquick Gel Extraction Kit (Qiagen) and were additionally purified using MinElute PCR Purification Kit (Qiagen). Each PCR product was quantified using the Qubit dsDNA HS assay (Thermo Fisher Scientific) and 25 ng of PCR product at 4 nM were submitted for sequencing, before which samples went through quality control using Bioanalyzer (Agilent). Libraries were sequenced using HiSeq 4000 with 100-bp reads (30 million reads per sample).

### CRISPR sequencing analysis

Raw data were trimmed to include 20 bp after the first occurrence of ‘CACCG’ in the read sequence. Trimmed reads were then mapped with BWA (version 0.5.9-r16)^84^ to a database of guide sequences for the human CRISPR Brunello lentiviral pooled library downloaded from Addgene (https://www.addgene.org/pooled-library/broadgpp-human-knockout-brunello/) with the parameters ‘-l 20 -k 2 -n 2’. sgRNA counts were obtained after filtering the mapped reads for those that had zero mismatches and mapped to the forward strand of the guide sequence. The MAGeCK ‘test’ command (version 0.5.7)^85^ was used to perform the sgRNA ranking analysis between the relevant conditions with parameters ‘–norm-method total–remove-zero both’. Two pairwise comparisons of samples were done: WT ‘day 6’ vs ATRX^KO^ ‘day 6’ and WT ‘day 16’ vs ATRX^KO^ ‘day 16’.

### Gene Ontology and clustering analysis

Dropout hits (top 50 for day 6, top 100 for day 16) were mapped on the protein interaction network through STRING (https://string-db.org), with a minimum required interaction score of 0.4. Subsequently, functional enrichments in both networks were analyzed against Gene Ontology (GO) terms Molecular Function, Biological Process and Cellular Component. A whole genome background was assumed for statistical analysis of GO enrichment. Statistical results (FDR) are presented as *P* values corrected for multiple testing in each category using the Benjamini–Hochberg procedure.

### Analysis of sensitivity to DNA-replication inhibitors and checkpoint inhibitors

For eHAP iCas9 cells, 200 cells per well were seeded in opaque 96-well plates. For NCI-H460 cells, 400 cells per well were seeded in opaque 96-well plates. Cells were treated the following day with the range of concentrations indicated for each compound. The compounds used are hydroxyurea (Sigma), aphidicolin (Sigma), WEE1 inhibitor MK-1775 (Selleckchem) and ATR inhibitor AZD6738 (Selleckchem). After 5 days of treatment, survival was assessed using CellTiter-Glo (Promega) assay, with luminescence measured on a CLARIOStar microplate reader (BMG Labtech). For each cell line, luminescence values were normalized against the value of the untreated wells.

### Clonogenic survival assay

Four hundred or 200 eHAP iCas9 cells per well were seeded in 24-well plates (in technical triplicate). Colonies were grown for 6 days. Eight hundred NCI-H460 iCas9 cells per well were seeded in 24-well plates (in technical triplicate). Colonies were grown for 7 days. Colonies were then fixed and stained with 0.5% crystal violet solution with 20% methanol. Plates were scanned and analyzed using GelCount (Oxford Optronics). The mean value for the eHAP or NCI-H460 iCas9 wild-type cell line with integrated sgNTC or untreated was used for normalization.

### IncuCyte proliferation imaging

One thousand eHAP iCas9 cells per well were seeded in opaque 96-well plates (in technical triplicate) and grown in an IncuCyte S3 System (Sartorius), providing continuous live-cell imaging every 3-4 h up to 5–6 days post-seeding. The ‘Basic Analyzer’ confluence processing analysis tool was used to tailor phase segmentation and quantification. Three to four images were taken per time point per well, and the mean value for technical replicates is plotted.

### Two-colour competitive growth assays

eHAP iCas9 ATRX^KO^ or NCI-H460 iCas9 ATRX^KO^ cells were transduced with virus particles expressing sgRNAs either in lenti-sgRNA-GFP-NLS-P2A-puromycin or lenti-sgRNA-mCherry-NLS-P2A-puromycin constructs. The sgRNAs used targeted *AAVS1* (control) or a specific gene of interest (Supplementary Table 4). Selected transductants were mixed in a 1:1 ratio (12,000 cells each) and seeded in a 24-well plate. Cells were imaged for GFP and mCherry signals in an IncuCyte S5 System (Sartorius). During the course of the experiment, cells were subcultured upon reaching near-confluency. The ‘Basic Analyzer’ fluorescent object ratio analysis tool was used to tailor segmentation and quantification. Nine to 16 images were taken per time point per well. Mean values for *n* = 3 biological replicates are plotted.

### DNA fiber assay

eHAP iCas9 cells were pulse-labeled with 25 µM CldU and subsequently with 250 µM IdU, for 20 min each. Cells were then harvested by trypsinization. DNA fiber spreads were prepared by spotting 2 µl of cells (500,000 cells/ml in PBS) onto microscope slides followed by lysis with 7 µL of lysis buffer (200 mM Tris-HCl pH 7.4 and 50 mM EDTA, 0.5% SDS). Slides were then tilted to allow a stream of DNA to move slowly toward the bottom of the slide, and DNA spreads were fixed in methanol/acetic acid (3:1). Slides were subsequently denatured in HCl 2.5 M, washed, and blocked in 1% BSA/PBS. HCl-denatured fiber spreads were incubated with rat anti-bromodeoxyuridine (detects CldU, Abcam, ab6326, 1:1,200) and mouse anti-bromodeoxyuridine (detects IdU, B44, BD Biosciences, 1:500) for 1 h and incubated with Alexa-Fluor-555-conjugated anti-rat-IgG and Alexa-Fluor-488-conjugated anti-mouse-IgG (both at 1:500, Thermo Fisher Scientific) for 1.5 h. Slides were mounted in Fluoroshield (Sigma) or Prolong Gold (Thermo Fisher Scientific). Images were acquired using a Zeiss Axio Imager M1 microscope, equipped with an ORCA-ER digital camera (Hamamatsu) controlled by Volocity 6.3 software (Perkin Elmer). Fiber length was analyzed using ImageJ (NIH). For fork speed analysis, a minimum of 100 fibers were measured per condition in each independent experiment. Fork asymmetry was calculated as the percentage of the length ratio of the shortest to the longest fiber of first label origin fibers.

For fork-degradation fibers, cells were labeled as above, followed by treatment with 4 mM hydroxyurea for 3 h. Fibers were spread, fixed and stained as described above. A ratio of IdU (green tract) over CldU (red tract) lengths was calculated to assess the extent of fork protection following treatment with hydroxyurea. A minimum of 100 fibers were measured per condition per independent replicate.

### Whole-cell extracts

For whole-cell lysates, cells were rinsed with PBS, trypsinized and collected in growth medium. Cells were pelleted by centrifugation at 500*g* for 5 min and washed once with PBS. Cell pellets were frozen on dry ice and stored at −80 °C. For lysis, cell pellets were thawed on ice and resuspended in RIPA Buffer (10 mM Tris-Cl pH 8.0, 1 mM EDTA, 0.5 mM EGTA, 1% Triton X-100, 0.1% sodium deoxycholate, 0.1% SDS, 140 mM NaCl, 1× phosphatase (PhosSTOP, Roche) and protease (cOmplete, EDTA-free, Roche) inhibitor mixes) and incubated on ice for 20 min. Lysates were sonicated with a probe at medium intensity for 10 s in a Soniprep 150 instrument and clarified by centrifugation at 13,000*g* for 15 min at 4 °C. Protein concentration was determined using the DC Protein Assay (Bio-Rad), according to the manufacturer’s instructions. Proteins were denatured in 2× NuPAGE LDS sample buffer (Invitrogen) and 1% 2-mercaptoethanol (Sigma) for 5 min at 95 °C. Lysates were frozen on dry ice and stored at −80 °C.

### SDS–PAGE and immunoblotting

Proteins were separated by SDS–PAGE using NuPAGE mini gels (Invitrogen) and transferred onto 0.2-µm pore nitrocellulose membrane (Amersham Protran; Sigma) using standard procedures. Membranes were blocked with 5% skim milk/TBST (TBS/0.1%Tween-20) for 1 h at room temperature (RT) and probed with the indicated primary antibodies overnight at 4 °C. Membranes were then washed 3 times for 10 min with TBST, incubated with appropriate secondary antibodies conjugated to horseradish peroxidase (HRP) for 1 h at RT and washed again 3 times for 10 min with TBST. All incubations were carried out on a horizontal shaker. Immunoblots were developed using Clarity or Clarity Max Western ECL Substrate (Bio-Rad). Chemiluminescence was acquired using a ChemiDoc MP imaging system (Bio-Rad).

### Cell-cycle analysis

For EdU and DAPI flow cytometry, eHAP iCas9 cells were labeled for 15 min with 10 µM EdU, fixed in 4% formaldehyde, permeabilized in 0.5% Triton X-100 and PBS and washed in 1% BSA and PBS before samples were processed using the Click-iT EdU Flow Cytometry Assay (Thermo Fisher Scientific) with Alexa Fluor 488. DNA was counterstained with DAPI (2 µg ml^−1^). Newly synthesized DNA (EdU) and DNA content (DAPI) were detected using a BD LSRFortessa analyzer (Becton Dickinson). Gating of single cells and cell-cycle analysis was performed manually using FlowJo.

### Fluorescence in situ hybridization in metaphase chromosome spreads

Cells were arrested in metaphase by incubation for 4 h in medium containing 100 ng ml^−1^ nocodazole (Sigma). Mitotic cells were collected, pelleted and swelled in a hypotonic solution (IMDM:deionized water at 1:3 ratio) for 6 min at RT. Subsequently, cells were fixed dropwise with freshly made Carnoy’s buffer (methanol:glacial acetic acid (3:1)) for 5 min at room temperature (RT) and spun down; this fixation step was repeated three times. The cell suspension in Carnoy’s buffer was stored at −20 °C. For spreading, the cell suspension (100 µl) was dropped on clean glass slides and dried overnight at RT.

The chromosome-spread slides were rehydrated in PBS for 5 min, fixed in 4% formaldehyde for 5 min, treated with 1 mg ml^−1^ pepsin for 10 min at 37 °C and fixed in 4% formaldehyde for 5 min. Next, slides were dehydrated in 70%, 85% and 100% (vol/vol) ethanol for 15 min each and then air-dried. Metaphase chromosome spreads were hybridized with a telomeric TelC-Cy5 PNA probe (PNA Bio) and centromeric CENPB-Cy3 probe (PNA Bio) in hybridizing solution (70% formamide, 0.5% blocking reagent (Roche), 10 mM Tris-HCl pH 7.2) for 90 s at 80 °C, followed by 1.5 h at RT. Slides were washed twice with washing buffer (70% formamide, 10 mM Tris-HCl pH 7.2) for 15 min at RT. Slides were mounted using ProLong Gold antifade with DAPI (Life Technologies). Chromosome images and telomere signals were captured using a Nikon Ti2 microscope fitted with a Prime 95B camera (Photometrics) using a Plan Apochromat ×100, 1.45-numerical-aperture oil objective lens, controlled by Nikon NIS-Elements.

### Immunofluorescence microscopy coupled to fluorescence in situ hybridization

Cells were grown on no. 1.5 18-mm glass coverslips. Cells were fixed with 4% formaldehyde in PBS for 15 min at RT. After fixation, cells were washed with PBS three times and then processed for immunofluorescence. Cells were blocked with ADB (Antibody Dilution Buffer; 10% normal goat serum, 0.1% Triton X-100, 0.1% saponin in PBS) for 1 h at RT. Cells were incubated with primary antibodies (diluted in ADB) for 1 h at RT, washed three times with PBS and then counterstained with Alexa-Fluor-conjugated secondary antibodies raised in goat (Thermo Fisher Scientific) diluted in ADB, for 1 h at RT. Cells were then washed three times with PBS. Cells were then fixed again with 4% formaldehyde in PBS for 15 min at RT and then washed twice with PBS. Next, coverslips were dehydrated in 70%, 85% and 100% (vol/vol) ethanol for 5 min each and then air-dried. Dry coverslips were hybridized with a telomeric TelC-Cy5 PNA probe (PNA Bio) in hybridizing solution (70% formamide, 0.5% blocking reagent (Roche), 10 mM Tris-HCl pH 7.2) for 90 s at 80 °C followed by 2 h at RT and washed twice with washing buffer (70% formamide, 10 mM Tris-HCl pH 7.2) for 15 min at RT. The coverslips were mounted onto glass slides with ProLong Gold antifade with DAPI (Life Technologies). Images were acquired using a Nikon Ti2 microscope fitted with a CSU-W1 spinning disk confocal unit (Yokogawa) and a Prime 95B camera (Photometrics) using Nikon CFI Apochromat LWD Lambda S ×40, 1.15-water-immersion objective lens and controlled by Nikon NIS-Elements. Following acquisition, images were imported into Fiji (NIH) for automated foci counting and colocalization quantitation (https://github.com/todd-fallesen/Sandra_Segura_Bayona_Diana).

### C-circle assay

The C-circle assay protocol was adapted from Henson et al.^86^, by using the quick C-circle preparation (QCP) protocol. DNA concentration was measured through fluorimetry using the Qubit dsDNA HS Assay (Thermo Fisher Scientific). Samples were pre-diluted in QCP lysis buffer at 30 ng µl^−1^. Then, 30 ng DNA was diluted to 10 µl in 10 mM Tris-HCl pH 7.6 and mixed with 9.25 µl Rolling Circle Master Mix (RCMM) (8.65 mM DTT, 2.16X phi29 buffer, 8.65 µg ml^−1^ BSA, 0.216% Tween-20 and 2.16 mM of each dATP, dCTP, dGTP and dTTP) and 0.75 µl of phi29 DNA Polymerase (Thermo Fisher Scientific). Rolling circle amplification was performed by incubating samples in a thermocycler at 30 °C for 8 h, polymerase was inactivated at 70 °C for 20 min and then kept at 8–10 °C. For slot blot detection, samples were blotted onto Amersham Hybond N+ positively charged nylon membranes (GE Healthcare) under native conditions. After cross-linking, membranes were hybridized with non-radioactive 3′-DIG-labeled probes, as previously described^87^. Membranes were developed using anti-DIG-AP (Roche) and CDP-Star substrate (Roche). Chemiluminescence was acquired using a ChemiDoc MP imaging system (Bio-Rad). Membranes were stripped and re-hybridized with 3′-DIG-labeled Alu probe as a loading control.

### Native fluorescence in situ hybridization (ssCCCTAA/ssTTAGGG)

Cells were grown on coverslips in 12-well plates or 96-well microplates (PerkinElmer) and fixed in 2% formaldehyde for 15 min at RT. After fixation, cells were washed with PBS twice and then processed for native fluorescence in situ hybridization. Cells were incubated with 250 μg ml^−1^ RNase A in ADB blocking solution (Antibody Dilution Buffer; 10% normal goat serum, 0.1% Triton X-100, 0.1% saponin in PBS) for 1 h at 37 °C. Next, coverslips and plates were dehydrated in 70%, 85% and 100% (vol/vol) ethanol for 5 min each and then air-dried. Dry coverslips and plates were hybridized with a telomeric TelG-TAMRA or TelG-CY3 PNA probe (PNA Bio) (for ssCCCTAA) or TelC-Cy5 PNA probe (PNA Bio) (for ssTTAGGG) in hybridizing solution (70% formamide, 1 mg ml^−1^ blocking reagent (Roche), 10 mM Tris-HCl pH 7.5) for 2 h at RT and washed twice with PBS. Nuclei were stained with incubation for 5 min with DAPI in PBS, followed by three washes with PBS. Coverslips were mounted in ProLong Gold antifade with DAPI (Life Technologies) and imaged in a Nikon Ti2 microscope fitted with a Prime 95B camera (Photometrics) using Plan Apochromat ×60, 1.4-numerical-aperture oil objective lens and controlled by Nikon NIS-Elements. Alternatively, plates were sealed and scanned using an Opera Phenix Plus High-Content Screening System (PerkinElmer), where images were captured using a ×40 objective and analyzed using Harmony High-Content Imaging and Analysis Software (PerkinElmer).

### Telomere G-overhang assay

For the analysis of human telomeric DNA by in gel hybridization, the protocol was adapted from Wu et al.^15^. Cells were trypsinized and resuspended in PBS. They were then mixed 1:1 with 2% agarose (Lonza) in PBS to obtain a concentration of one million cells per plug. Plugs were digested with 1 mg ml^−1^ proteinase K in digest buffer (100 mM EDTA pH 8.0, 0.2% sodium deoxycholate, 1% sarcosyl) overnight at 50 °C. Plugs were washed four times for 1 h each in TE buffer, with 1 mM PMSF in the last wash, once with sterile water for 30 min and with restriction enzyme buffer (CutSmart) for 30 min. Plugs were incubated with 60 units MboI in restriction enzyme buffer overnight at 37 °C. The following day, the plugs were rinsed once in TE, and once in 0.5× TBE, and loaded onto a 1% agarose/0.5× TBE gel and separated by PFGE for 18–24 h (CHEF DR III, Bio-Rad) in 0.5× TBE. The gels were dried and prehybridized in Church mix for 1 h at 50 °C. Hybridization with radioactively labeled γ-32P-ATP [AACCCT]_4_ was performed overnight at 50 °C in Church mix. The gel was washed at 55 °C three times for 30 min each in 4× SSC and once for 30 min in 0.1% SDS and 4× SSC. The gel was exposed to a phosphorimaging plate (GE Healthcare) and scanned using Typhoon FLA 9500 (GE Healthcare). The gel was subsequently denatured in 1.5 M NaCl, 0.5 M NaOH for 30 min, neutralized with two 15-min washes in 0.5 M Tris-HCl pH 7.5, 3 M NaCl and pre-hybridized with the same probe overnight at 55 °C. The gel was washed, exposed and imaged as described above. The single-stranded overhang signal in the native gel relative to the total telomeric DNA in the denatured gel was quantified using Fiji (NIH).

### Telomerase activity assay

Telomerase activity in cell lysates was measured by TRAP assay using the TRAPeze RT Telomerase Detection Kit (Millipore, cat. no. S7710), according to the manufacturer’s instructions. In brief, 300,000 cells were lysed in CHAPS lysis buffer supplemented with 200 U/reaction RNaseOUT (Invitrogen, cat. no. 10000840), and cell extracts equivalent to 3,000 cells per reaction were assayed using Titanium Taq DNA Polymerase (TaKaRa, cat. no. 639209). Relative telomerase activity was determined from a linear plot of log_10_-transformed TSR8 control template quantities versus their corresponding Ct values. For telomerase inhibitor samples, cell lysates were incubated with 15 μM BIBR1532 (SelleckChem, cat. no. S1186) for 30 min at 30 °C before the TRAPeze reaction. All samples were assayed in triplicate and included non-treated and heat-inactivated controls on the same plate. Real-time fluorescence data were acquired using a StepOnePlus Real-Time PCR System (Thermo Fisher Scientific).

### SILAC labeling

eHAP cells were grown in IMDM for SILAC (Thermo Fisher Scientific) supplemented with 10% (vol/vol) SILAC dialyzed FBS (Gemini Biosciences) and 1% penicillin–streptomycin. Cells designated as ‘heavy’ were cultured in medium supplemented with L-lysine (13C6/15N2), L-arginine (13C6/15N4) (both from Cambridge Isotope Laboratories) and L-proline, while cells designated as ‘light’ were cultured in medium supplemented with unlabelled L-lysine, L-arginine and L-proline. Cells were passaged for at least 10–12 population doublings, and a labelling efficiency test was performed to confirm label incorporation.

### iPOND

Asynchronous eHAP WT and ATRX^KO^ cultures were pulsed with 10 µM EdU for 10 min. For HU chase, cells were pulsed with 10 µM EdU for 10 min, washed once in pre-equilibrated chase medium, and treated with 0.5 mM HU for 60 min. For the thymidine chase, cells were pulsed with 10 µM EdU for 10 min, washed once in pre-equilibrated chase medium, and treated with 10 µM thymidine for 60 min. Cells were immediately crosslinked with 1% formaldehyde in PBS for 20 min at RT, quenched for 20 min with 0.125 M glycine in PBS, collected by scraping, and washed three times in cold PBS. For SILAC, heavy and light cells were pooled 1:1 at this stage. Pellets were resuspended in 10 ml permeabilization buffer (0.25% (vol/vol) Triton X-100 in PBS), incubated at RT for 30 min and spun at 1,000*g* for 5 min. The pellets were washed in 10 ml 0.5% (wt/vol) BSA in PBS, spun at 1,000*g* for 5 min and resuspended in Click Reaction cocktail (in a total volume of 5 ml in PBS: 10 µM biotin azide, 10 mM sodium ascorbate, 2 mM CuSO_4_) and incubated for 2 h at RT. The pellets were then washed in 0.5% (wt/vol) BSA in PBS, spun at 1,000*g* for 5 min, resuspended in 700 µl nuclear lysis buffer (10 mM Tris-Cl pH 8.0, 1 mM EDTA, 0.5 mM EGTA, 1% Triton X-100, 0.1% sodium deoxycholate, 0.1% SDS, 140 mM NaCl, 1× phosphatase (PhosSTOP, Roche) and protease (cOmplete, EDTA-free, Roche) inhibitor mixes), incubated on ice for 15 min, sonicated in a Bioruptor Pico instrument (Diagenode) for a total of 25 cycles (30 s on, 30 s off) and spun in a microcentrifuge at maximum speed for 10 min. Lysate was diluted 1:1 with cold PBS and subjected to pull-down with 50 µl Sera-Mag SpeedBeads Neutravidin-Coated Magnetic Particles (Cytiva) overnight at 4 °C. Beads were washed four times with Lysis buffer, eluted for 20 min at 95 °C in 2× NuPAGE LDS sample buffer (Invitrogen) and 2% 2-mercaptoethanol (Sigma), and eluate was run 3 cm into a 10% NuPAGE Bis-Tris gel (Invitrogen) for mass-spectrometry analysis.

### Immunoprecipitation

eHAP ATRX-KO cells stably complemented with ATRX-HA were lysed by incubating them for 40 min under constant agitation in IP buffer (10 mM Tris pH 7.5, 150 mM NaCl, 0.5 mM EDTA, 2.5 mM MgCl_2_, 0.5% Triton X-100) complemented with 1× phosphatase (PhosSTOP, Roche) and protease (cOmplete, EDTA-free, Roche) inhibitor mixes and benzonase (E1014, Sigma). Cells were then centrifuged at 15,000*g* for 15 min and the cleared cell lysates were diluted in dilution buffer (10 mM Tris pH 7.5, 150 mM NaCl, 0.5 mM EDTA, 2.5 mM MgCl_2_) complemented with 1× phosphatase (PhosSTOP, Roche) and protease (cOmplete, EDTA-free, Roche) inhibitor mixes. Diluted lysates were immunoprecipitated using 30 μl Pierce anti-HA magnetic beads (cat. no. 88826, Thermo) overnight at 4 °C. Beads were washed three times for 10 min with dilution buffer buffer containing 0.05% NP-40 and eluted and boiled in 2× NuPAGE LDS sample buffer (Invitrogen) and 1% 2-mercaptoethanol (Sigma).

### Chromatin fractionation

eHAP iCas9 cells were seeded 36–48 h prior to collection. Asynchronous cultures were trypsinized and resuspended in ice-cold PBS. Three million cells were kept on ice for whole-cell lysate control. Three million cells were spun down for 5 min at 500*g* and resuspended in 200 µl CSK buffer (10 mM PIPES pH 7.0, 100 mM NaCl, 300 mM sucrose, 1.5 mM MgCl_2_, 5 mM EDTA, 0.5% Triton X-100, 1× phosphatase (PhosSTOP, Roche) and protease (cOmplete, EDTA-free, Roche) inhibitor mixes) and incubated on ice for 10 min. Cells were spun down at full speed for 10 s and the supernatant (soluble fraction) was collected. The chromatin pellet was washed in 500 µl of CSK buffer. Chromatin pellets were resuspended in 200 µl 1× NuPAGE LDS sample buffer (Invitrogen) and 1% 2-mercaptoethanol (Sigma). Fifty microliters of 4× NuPAGE LDS sample buffer (Invitrogen) and 4% 2-mercaptoethanol (Sigma) was added to 150 µl whole-cell lysate samples. All samples were boiled at 95 °C for 10 min and sonicated with a probe at medium intensity for 10 s in a Soniprep 150 instrument. Twenty microliters of each fraction was loaded and subjected to SDS–PAGE, as described above.

### Cell-cycle synchronization and S-phase entry analysis

eHAP and NCI-H460 cells were synchronized at the G1/S boundary using a double thymidine block. For this purpose, cells were incubated with 2 mM thymidine for 16 h, released into fresh medium for 8 h and treated again with 2 mM thymidine for 16 h. For S-phase entry, cells were released into thymidine-free medium for 4 h. To measure S-phase progression, cells were pulsed with 100 µM EdU for 30 min, fixed in 4% paraformaldehyde, permeabilized with 0.2% Triton X-100 in PBS for 10 min and processed using the Click-iT EdU Alexa Fluor 647 Imaging Kit (Invitrogen), according to the manufacturer’s protocol. Nuclei were counterstained with DAPI. Stained plates were scanned using the Operetta CLS confocal high-content system with a ×20 objective. Single image slices were used for phenotypic analysis on 45 fields of view. Harmony 5.1 software was used for primary nuclei segmentation and quantification of EdU intensity to determine the cell-cycle stage.

### Chromatin fractionation of synchronized cells

Cells were collected and lysed in TEB150 buffer (50 mM HEPES pH 7.4, 150 mM NaCl, 2 mM MgCl_2_, 1 mM dithiothreitol (DTT), 0.5% Triton X-100 and 10% glycerol) supplemented with protease inhibitor cocktail (Roche) and phosphatase inhibitor cocktail (PhosSTOP, Sigma) for 45 min on ice, followed by centrifugation (15 min at 16,000*g*). The supernatant was collected as the soluble fraction. The pellet was washed twice with TEB150 buffer and centrifuged for 6 min at 16,000*g* each time. The final pellet (chromatin fraction) was resuspended in TEB150 buffer containing Benzonase (Sigma). Samples were mixed with SDS sample buffer, heated for 5 min at 90 °C and subjected to SDS–PAGE, as described above.

### Proximity ligation assay

Cells were seeded on 13-mm coverslips in a 24-well plate. The next day, cells were pulse-labeled with 10 µM EdU for 10 min, followed by pre-extraction in CSK buffer (10 mM HEPES pH 7.5, 100 mM NaCl, 300 mM sucrose, 3 mM MgCl_2_) containing 0.2% Triton X-100 for 5 min on ice. Cells were immediately fixed in 4% (vol/vol) PFA in PBS for 15 min at room temperature. Next, epitope exposure and permeabilization were performed with ice-cold 100% methanol for 10 min. Following permeabilization, cells were washed three times in PBS, and EdU click reactions were performed by incubating cells with Click reaction buffer containing 2 mM CuSO_4_, 100 mM sodium ascorbate (prepared fresh) and 1 μM Alexa Fluor 647 azide (Invitrogen cat. no. A10277) in PBS for 30 min at room temperature. Next, a proximity ligation assay (PLA) was performed using the Duolink in situ red starter kit (Sigma-Aldrich), following the manufacturer’s instructions. In brief, cells were incubated in blocking buffer for 1 h at room temperature followed by incubation with rabbit anti-HA-Tag (Cell Signaling Technology cat. no. 3724, 1:250) and mouse anti-PCNA (Santa Cruz Bio sc-56, 1:250) antibodies for 1 h at 37 °C. Coverslips were then washed with Duolink Wash Buffer A and incubated with anti-mouse MINUS and anti-rabbit PLUS PLA probes for 1 h at 37 °C. Next, ligation and amplification reaction were performed, following the manufacturer’s instructions. Finally, nuclei were counterstained with DAPI and mounted on slides using the Prolong Gold Antifade reagent (Thermo Fisher Scientific). Images were acquired using Nikon Ti2-E inverted microscope equipped with a Nikon Plan Apo ×60, 1.40 Oil objective lens and Photometrics Prime 95B camera. PLA foci were detected and analyzed using CellProfiler, and data are presented as the number of foci per nucleus.

### Chromatin immunoprecipitation and slot blot

Cells were cross-linked with 1% methanol-free formaldehyde in PBS for 10 min at room temperature and quenched with 125 mM glycine for 5 min. Cells were then rinsed twice with PBS and subsequently collected by scraping on ice with cold 0.05% Tween in PBS. Cells were pelleted by centrifugation and flash frozen in liquid nitrogen. For chromatin extraction, pellets of about ten million cells were sequentially resuspended and incubated for 10 min in rotation at 4 °C first in lysis buffer 1 (50 mM HEPES pH 7.9, 140 mM NaCl, 10% glycerol, 0.5% NP-40, 0.25% Triton X-100, 1 mM EDTA) and then in lysis buffer 2 (10 mM HEPES pH 8, 200 mM NaCl, 1 mM EDTA, 0.5 mM EGTA). Nuclei were collected by centrifugation after both lysis steps. All buffers were supplemented with protease and phosphatase inhibitors. Pellets were resuspended in lysis buffer 3 (10 mM HEPES pH 8, 100 mM NaCl, 1 mM EDTA, 0.5 mM EGTA, 0.1% sodium deoxycholate, 0.5% *N*-lauroylsarcosine sodium salt solution) and transferred in 1 ml AFA Fiber milliTUBE (Covaris 520135). Chromatin was sheared to about 300-bp fragments with a Covaris LE220, with the following settings: duty factor, 30%; peak incident power, 450; cycles per burst, 400; time, 10 min. The chromatin concentration was adjusted to about 1 mg ml^−1^. The lysate was supplemented with Triton X-100 to a final concentration of 1%, then cleared (16,000*g*, 10 min at 4 °C); 50 µl supernatant was kept as input, and 950 µl was incubated overnight at 4 °C with 5 µg of H3.3 antibody (Active Motif, cat. no. 91191). The immunoprecipitated complexes were recovered with 50 µl of a 1:1 mixture of protein A and G Dynabeads (2 h in rotation at 4 °C). Beads were then washed four times with wash buffer (50 mM HEPES pH 7.5, 250 mM LiCl, 0.7% Na-deoxycholate, 1% NP-40, 1 mM EDTA) and once with TE pH 8. Elution was performed with 100 µl elution buffer (50 mM Tris-HCl pH 8, 1% SDS, 10 mM EDTA) at 65 °C for 10 min. DNA was recovered after incubation first with 80 µg of RNase A at 37 °C for 30 min, then with 80 µg of proteinase K at 55 °C overnight and purified with the QIAquick PCR purification kit. For the slot blot, the DNA was first denatured for 30 min at 55 °C with 2 volumes of 0.5 M NaOH and 1.5 M NaCl, then neutralized with 3.3 volumes of neutralizing solution (0.5 M Tris-HCl pH 7.5, 1.5 M NaCl). The DNA was then applied to a Hybond N+ positively charged nylon membrane (Amersham) and cross-linked in a 254 nm UV cross-linker (120 mJ cm^−^^2^). The membrane was then incubated overnight with a 3’-DIG-labeled probe and processed as previously described^87^.

### Statistical analysis

Sample number (*n*) indicates the number of independent biological samples in each experiment and are indicated in figure legends. Statistical details of each experiment (including the statistical tests used and the exact value of *n*) can be found in the figure legends. Prism 9/10 (GraphPad) and RStudio were used for statistical analysis: one-way ANOVA, followed by Tukey’s multiple-comparison test, was used unless stated otherwise.

### Reporting summary

Further information on research design is available in the Nature Portfolio Reporting Summary linked to this article.

## Online content

Any methods, additional references, Nature Portfolio reporting summaries, source data, extended data, supplementary information, acknowledgements, peer review information; details of author contributions and competing interests; and statements of data and code availability are available at 10.1038/s41594-026-01827-2.

## Supplementary information

Reporting Summary

Supplementary Tables 1–5 Supplementary Table 1. First three tabs: mAGeCK analysis of sequenced sgRNA reads from genome-wide CRISPR–Cas9 screens in eHAP iCas9 wild type versus *ATRX*-null cells. Comparisons from day 6 and 16 are shown. Supplementary Table 2 (fourth tab). BLISS score analysis for validated synthetic lethal interactions with *ATRX*. An unpaired *t*-test was used for the statistical analysis of actual vs theoretical values. Supplementary Table 3 (fifth tab). Processed iPOND–SILAC mass-spectrometry data set. Supplementary Table 4 (sixth tab). Target sequences of sgRNA used for cell line generation (full, inducible knockouts) and crRNA transfections. Supplementary Table 5 (seventh tab). Oligonucleotides used in this study.

## Source data

Source Data Figs. 1–6 Single file containing all source data for main figures.

Source Data Figs. 1–6 Single file with clearly labeled unprocessed blots for each figure.

Source Data Extended Data Figs. 1–9 Single file containing all source data for extended figures.

Source Data Extended Data Figs. 1–9 Single file with clearly labeled unprocessed blots for each extended figure.

## Data Availability

Sequencing read raw counts from CRISPR screens generated in this study have been deposited at Mendeley data (10.17632/4wyg87kxrr.1). Raw proteomics data have been deposited in the PRIDE repository under accession code PXD068487. The code used for image analysis of nuclear foci and colocalization in IF-FISH assays has been deposited at GitHub (10.5281/zenodo.10069688). Any requests for resources or reagents should be directed to and will be fulfilled by the corresponding author. Source data are provided with this paper.

## References

[1] Saxena, S. & Zou, L. Hallmarks of DNA replication stress. *Mol. Cell* **82**, 2298–2314 (2022). 35714587 10.1016/j.molcel.2022.05.004 PMC9219557

[2] Zou, L. & Elledge, S. J. Sensing DNA damage through ATRIP recognition of RPA–ssDNA complexes. *Science* **300**, 1542–1548 (2003). 12791985 10.1126/science.1083430

[3] Cortez, D., Guntuku, S., Qin, J. & Elledge, S. J. ATR and ATRIP: partners in checkpoint signaling. *Science* **294**, 1713–1716 (2001). 11721054 10.1126/science.1065521

[4] Delacroix, S., Wagner, J. M., Kobayashi, M., Yamamoto, K. & Karnitz, L. M. The Rad9–Hus1–Rad1 (9-1-1) clamp activates checkpoint signaling via TopBP1. *Genes Dev.* **21**, 1472–1477 (2007). 17575048 10.1101/gad.1547007 PMC1891424

[5] Haahr, P. et al. Activation of the ATR kinase by the RPA-binding protein ETAA1. *Nat. Cell Biol.* **18**, 1196–1207 (2016). 27723717 10.1038/ncb3422

[6] Bass, T. E. et al. ETAA1 acts at stalled replication forks to maintain genome integrity. *Nat. Cell Biol.* **18**, 1185–1195 (2016). 27723720 10.1038/ncb3415 PMC5245861

[7] Matsuoka, S. et al. ATM and ATR substrate analysis reveals extensive protein networks responsive to DNA damage. *Science* **316**, 1160–1166 (2007). 17525332 10.1126/science.1140321

[8] Liu, Q. et al. Chk1 is an essential kinase that is regulated by Atr and required for the G_2_/M DNA damage checkpoint. *Genes Dev.* **14**, 1448–1459 (2000). 10859164 PMC316686

[9] Saldivar, J. C., Cortez, D. & Cimprich, K. A. The essential kinase ATR: ensuring faithful duplication of a challenging genome. *Nat. Rev. Mol. Cell Biol.* **18**, 622–636 (2017). 28811666 10.1038/nrm.2017.67 PMC5796526

[10] Sfeir, A. et al. Mammalian telomeres resemble fragile sites and require TRF1 for efficient replication. *Cell* **138**, 90–103 (2009). 19596237 10.1016/j.cell.2009.06.021 PMC2723738

[11] Hom, R. A. & Wuttke, D. S. Human CST prefers G-rich but not necessarily telomeric sequences. *Biochemistry* **56**, 4210–4218 (2017). 28726394 10.1021/acs.biochem.7b00584 PMC6151266

[12] Chen, L. Y., Redon, S. & Lingner, J. The human CST complex is a terminator of telomerase activity. *Nature* **488**, 540–544 (2012). 22763445 10.1038/nature11269

[13] Zaug, A. J. et al. CST does not evict elongating telomerase but prevents initiation by ssDNA binding. *Nucleic Acids Res.* **49**, 11653–11665 (2021). 34718732 10.1093/nar/gkab942 PMC8599947

[14] Wang, F. et al. Human CST has independent functions during telomere duplex replication and C-strand fill-in. *Cell Rep.* **2**, 1096–1103 (2012). 23142664 10.1016/j.celrep.2012.10.007 PMC3513692

[15] Wu, P., Takai, H. & de Lange, T. Telomeric 3’ overhangs derive from resection by Exo1 and Apollo and fill-in by POT1b-associated CST. *Cell* **150**, 39–52 (2012). 22748632 10.1016/j.cell.2012.05.026 PMC3392515

[16] Takai, H., Aria, V., Borges, P., Yeeles, J. T. P. & de Lange, T. CST-polymerase alpha-primase solves a second telomere end-replication problem. *Nature* **627**, 664–670 (2024). 38418884 10.1038/s41586-024-07137-1 PMC11160940

[17] Stewart, J. A. et al. Human CST promotes telomere duplex replication and general replication restart after fork stalling. *EMBO J.* **31**, 3537–3549 (2012). 22863775 10.1038/emboj.2012.215 PMC3433780

[18] Chastain, M. et al. Human CST facilitates genome-wide RAD51 recruitment to GC-Rich repetitive sequences in response to replication stress. *Cell Rep.* **16**, 2048 (2016). 27533181 10.1016/j.celrep.2016.08.008 PMC5669620

[19] Wang, Y. & Chai, W. Pathogenic CTC1 mutations cause global genome instabilities under replication stress. *Nucleic Acids Res.* **46**, 3981–3992 (2018). 29481669 10.1093/nar/gky114 PMC5934659

[20] Lei, K. H. et al. Crosstalk between CST and RPA regulates RAD51 activity during replication stress. *Nat. Commun.* **12**, 6412 (2021). 34741010 10.1038/s41467-021-26624-x PMC8571288

[21] Lyu, X. et al. Human CST complex protects stalled replication forks by directly blocking MRE11 degradation of nascent-strand DNA. *EMBO J.* **40**, e103654 (2021). 33210317 10.15252/embj.2019103654 PMC7809791

[22] Zhang, M. et al. Mammalian CST averts replication failure by preventing G-quadruplex accumulation. *Nucleic Acids Res.* **47**, 5243–5259 (2019). 30976812 10.1093/nar/gkz264 PMC6547417

[23] Mirman, Z. et al. 53BP1-RIF1-shieldin counteracts DSB resection through CST- and Polalpha-dependent fill-in. *Nature* **560**, 112–116 (2018). 30022158 10.1038/s41586-018-0324-7 PMC6072559

[24] Gibbons, R. J., Picketts, D. J., Villard, L. & Higgs, D. R. Mutations in a putative global transcriptional regulator cause X-linked mental retardation with alpha-thalassemia (ATR-X syndrome). *Cell* **80**, 837–845 (1995). 7697714 10.1016/0092-8674(95)90287-2

[25] Cerami, E. et al. The cBio cancer genomics portal: an open platform for exploring multidimensional cancer genomics data. *Cancer Discov* **2**, 401–404 (2012). 22588877 10.1158/2159-8290.CD-12-0095 PMC3956037

[26] Gao, J. et al. Integrative analysis of complex cancer genomics and clinical profiles using the cBioPortal. *Sci. Signal* **6**, pl1 (2013). 23550210 10.1126/scisignal.2004088 PMC4160307

[27] Goldberg, A. D. et al. Distinct factors control histone variant H3.3 localization at specific genomic regions. *Cell* **140**, 678–691 (2010). 20211137 10.1016/j.cell.2010.01.003 PMC2885838

[28] Lewis, P. W., Elsaesser, S. J., Noh, K. M., Stadler, S. C. & Allis, C. D. Daxx is an H3.3-specific histone chaperone and cooperates with ATRX in replication-independent chromatin assembly at telomeres. *Proc. Natl Acad. Sci. USA* **107**, 14075–14080 (2010). 20651253 10.1073/pnas.1008850107 PMC2922592

[29] Drane, P., Ouararhni, K., Depaux, A., Shuaib, M. & Hamiche, A. The death-associated protein DAXX is a novel histone chaperone involved in the replication-independent deposition of H3.3. *Genes Dev.* **24**, 1253–1265 (2010). 20504901 10.1101/gad.566910 PMC2885661

[30] Law, M. J. et al. ATR-X syndrome protein targets tandem repeats and influences allele-specific expression in a size-dependent manner. *Cell* **143**, 367–378 (2010). 21029860 10.1016/j.cell.2010.09.023

[31] Wong, L. H. et al. ATRX interacts with H3.3 in maintaining telomere structural integrity in pluripotent embryonic stem cells. *Genome Res.* **20**, 351–360 (2010). 20110566 10.1101/gr.101477.109 PMC2840985

[32] Voon, H. P. et al. ATRX plays a key role in maintaining silencing at interstitial heterochromatic loci and imprinted genes. *Cell Rep.* **11**, 405–418 (2015). 25865896 10.1016/j.celrep.2015.03.036 PMC4410944

[33] Sadic, D. et al. Atrx promotes heterochromatin formation at retrotransposons. *EMBO Rep.* **16**, 836–850 (2015). 26012739 10.15252/embr.201439937 PMC4515123

[34] Teng, Y. C. et al. ATRX promotes heterochromatin formation to protect cells from G-quadruplex DNA-mediated stress. *Nat. Commun.* **12**, 3887 (2021). 34162889 10.1038/s41467-021-24206-5 PMC8222256

[35] Truch, J. et al. The chromatin remodeller ATRX facilitates diverse nuclear processes, in a stochastic manner, in both heterochromatin and euchromatin. *Nat. Commun.* **13**, 3485 (2022). 35710802 10.1038/s41467-022-31194-7 PMC9203812

[36] Clynes, D. et al. ATRX dysfunction induces replication defects in primary mouse cells. *PLoS ONE* **9**, e92915 (2014). 24651726 10.1371/journal.pone.0092915 PMC3961441

[37] Huh, M. S. et al. Stalled replication forks within heterochromatin require ATRX for protection. *Cell Death Dis.* **7**, e2220 (2016). 27171262 10.1038/cddis.2016.121 PMC4917659

[38] Nguyen, D. T. et al. The chromatin remodelling factor ATRX suppresses R-loops in transcribed telomeric repeats. *EMBO Rep.* **18**, 914–928 (2017). 28487353 10.15252/embr.201643078 PMC5452009

[39] Wang, Y. et al. G-quadruplex DNA drives genomic instability and represents a targetable molecular abnormality in ATRX-deficient malignant glioma. *Nat. Commun.* **10**, 943 (2019). 30808951 10.1038/s41467-019-08905-8 PMC6391399

[40] Juhasz, S., Elbakry, A., Mathes, A. & Lobrich, M. ATRX promotes DNA repair synthesis and sister chromatid exchange during homologous recombination. *Mol. Cell* **71**, 11–24(2018). 29937341 10.1016/j.molcel.2018.05.014

[41] Cotta-Ramusino, C. et al. A DNA damage response screen identifies RHINO, a 9-1-1 and TopBP1 interacting protein required for ATR signaling. *Science* **332**, 1313–1317 (2011). 21659603 10.1126/science.1203430 PMC4357496

[42] Drosopoulos, W. C., Kosiyatrakul, S. T., Yan, Z., Calderano, S. G. & Schildkraut, C. L. Human telomeres replicate using chromosome-specific, rather than universal, replication programs. *J. Cell Biol.* **197**, 253–266 (2012). 22508510 10.1083/jcb.201112083 PMC3328383

[43] Loe, T. K. et al. Telomere length heterogeneity in ALT cells is maintained by PML-dependent localization of the BTR complex to telomeres. *Genes Dev.* **34**, 650–662 (2020). 32217664 10.1101/gad.333963.119 PMC7197349

[44] Mazzucco, G. et al. Telomere damage induces internal loops that generate telomeric circles. *Nat. Commun.* **11**, 5297 (2020). 33082350 10.1038/s41467-020-19139-4 PMC7576219

[45] Huang, C., Jia, P., Chastain, M., Shiva, O. & Chai, W. The human CTC1/STN1/TEN1 complex regulates telomere maintenance in ALT cancer cells. *Exp. Cell. Res.* **355**, 95–104 (2017). 28366536 10.1016/j.yexcr.2017.03.058 PMC5551977

[46] Lee, J. et al. Extrachromosomal telomere DNA derived from excessive strand displacements. *Proc. Natl Acad. Sci. USA* **121**, e2318438121 (2024). 38696464 10.1073/pnas.2318438121 PMC11087782

[47] Lovejoy, C. A., Takai, K., Huh, M. S., Picketts, D. J. & de Lange, T. ATRX affects the repair of telomeric DSBs by promoting cohesion and a DAXX-dependent activity. *PLoS Biol.* **18**, e3000594 (2020). 31895940 10.1371/journal.pbio.3000594 PMC6959610

[48] Zhong, S. et al. A role for PML and the nuclear body in genomic stability. *Oncogene* **18**, 7941–7947 (1999). 10637504 10.1038/sj.onc.1203367

[49] Carbone, R., Pearson, M., Minucci, S. & Pelicci, P. G. PML NBs associate with the hMre11 complex and p53 at sites of irradiation induced DNA damage. *Oncogene* **21**, 1633–1640 (2002). 11896594 10.1038/sj.onc.1205227

[50] Marchesini, M. et al. PML is required for telomere stability in non-neoplastic human cells. *Oncogene* **35**, 1811–1821 (2016). 26119943 10.1038/onc.2015.246 PMC4830905

[51] Jiang, H. et al. BLM helicase unwinds lagging strand substrates to assemble the ALT telomere damage response. *Mol. Cell* **84**, 1684–1698 (2024). 38593805 10.1016/j.molcel.2024.03.011 PMC11069441

[52] Buisson, R., Boisvert, J. L., Benes, C. H. & Zou, L. Distinct but concerted roles of ATR, DNA-PK, and Chk1 in countering replication stress during S phase. *Mol. Cell* **59**, 1011–1024 (2015). 26365377 10.1016/j.molcel.2015.07.029 PMC4575890

[53] Kim, S. T., Lim, D. S., Canman, C. E. & Kastan, M. B. Substrate specificities and identification of putative substrates of ATM kinase family members. *J. Biol. Chem.* **274**, 37538–37543 (1999). 10608806 10.1074/jbc.274.53.37538

[54] Liang, J. et al. Genome-Wide CRISPR–Cas9 screen reveals selective vulnerability of ATRX-mutant cancers to WEE1 inhibition. *Cancer Res.* **80**, 510–523 (2020). 31551363 10.1158/0008-5472.CAN-18-3374

[55] Sirbu, B. M. et al. Identification of proteins at active, stalled, and collapsed replication forks using isolation of proteins on nascent DNA (iPOND) coupled with mass spectrometry. *J. Biol. Chem.* **288**, 31458–31467 (2013). 24047897 10.1074/jbc.M113.511337 PMC3814742

[56] Dungrawala, H. et al. The replication checkpoint prevents two types of fork collapse without regulating replisome stability. *Mol. Cell* **59**, 998–1010 (2015). 26365379 10.1016/j.molcel.2015.07.030 PMC4575883

[57] Wessel, S. R., Mohni, K. N., Luzwick, J. W., Dungrawala, H. & Cortez, D. Functional analysis of the replication fork proteome identifies BET proteins as PCNA regulators. *Cell Rep.* **28**, 3497–3509 (2019). 31553917 10.1016/j.celrep.2019.08.051 PMC6878991

[58] Mukherjee, C. et al. RIF1 promotes replication fork protection and efficient restart to maintain genome stability. *Nat. Commun.* **10**, 3287 (2019). 31337767 10.1038/s41467-019-11246-1 PMC6650494

[59] Alabert, C. et al. Nascent chromatin capture proteomics determines chromatin dynamics during DNA replication and identifies unknown fork components. *Nat. Cell Biol.* **16**, 281–293 (2014). 24561620 10.1038/ncb2918 PMC4283098

[60] Unger, S. et al. FAM111A mutations result in hypoparathyroidism and impaired skeletal development. *Am. J. Hum. Genet.* **92**, 990–995 (2013). 23684011 10.1016/j.ajhg.2013.04.020 PMC3675238

[61] Kojima, Y. et al. FAM111A protects replication forks from protein obstacles via its trypsin-like domain. *Nat. Commun.* **11**, 1318 (2020). 32165630 10.1038/s41467-020-15170-7 PMC7067828

[62] Hoffmann, S. et al. FAM111 protease activity undermines cellular fitness and is amplified by gain-of-function mutations in human disease. *EMBO Rep.* **21**, e50662 (2020). 32776417 10.15252/embr.202050662 PMC7534640

[63] Rios-Szwed, D. O. et al. FAM111A regulates replication origin activation and cell fitness. *Life Sci. Alliance* **6**, e202302111 (2023). 37793778 10.26508/lsa.202302111 PMC10551639

[64] Eustermann, S. et al. Combinatorial readout of histone H3 modifications specifies localization of ATRX to heterochromatin. *Nat. Struct. Mol. Biol.* **18**, 777–782 (2011). 21666677 10.1038/nsmb.2070

[65] Iwase, S. et al. ATRX ADD domain links an atypical histone methylation recognition mechanism to human mental-retardation syndrome. *Nat. Struct. Mol. Biol.* **18**, 769–776 (2011). 21666679 10.1038/nsmb.2062 PMC3130887

[66] Hoelper, D., Huang, H., Jain, A. Y., Patel, D. J. & Lewis, P. W. Structural and mechanistic insights into ATRX-dependent and -independent functions of the histone chaperone DAXX. *Nat. Commun.* **8**, 1193 (2017). 29084956 10.1038/s41467-017-01206-y PMC5662737

[67] Xue, Y. et al. The ATRX syndrome protein forms a chromatin-remodeling complex with Daxx and localizes in promyelocytic leukemia nuclear bodies. *Proc. Natl Acad. Sci. USA* **100**, 10635–10640 (2003). 12953102 10.1073/pnas.1937626100 PMC196856

[68] Tang, J. et al. A novel transcription regulatory complex containing death domain-associated protein and the ATR-X syndrome protein. *J. Biol. Chem.* **279**, 20369–20377 (2004). 14990586 10.1074/jbc.M401321200

[69] Wasylishen, A. R. et al. Daxx maintains endogenous retroviral silencing and restricts cellular plasticity in vivo. *Sci. Adv.* **6**, eaba8415 (2020). 32821827 10.1126/sciadv.aba8415 PMC7406367

[70] Pinto, L. M. et al. DAXX promotes centromeric stability independently of ATRX by preventing the accumulation of R-loop-induced DNA double-stranded breaks. *Nucleic Acids Res.* **52**, 1136–1155 (2024). 38038252 10.1093/nar/gkad1141 PMC10853780

[71] Tomaska, L., Cesare, A. J., AlTurki, T. M. & Griffith, J. D. Twenty years of t-loops: a case study for the importance of collaboration in molecular biology. *DNA Repair* **94**, 102901 (2020). 32620538 10.1016/j.dnarep.2020.102901 PMC8679138

[72] Surovtseva, Y. V. et al. Conserved telomere maintenance component 1 interacts with STN1 and maintains chromosome ends in higher eukaryotes. *Mol. Cell* **36**, 207–218 (2009). 19854131 10.1016/j.molcel.2009.09.017 PMC2768651

[73] Miyake, Y. et al. RPA-like mammalian Ctc1–Stn1–Ten1 complex binds to single-stranded DNA and protects telomeres independently of the Pot1 pathway. *Mol. Cell* **36**, 193–206 (2009). 19854130 10.1016/j.molcel.2009.08.009

[74] Huang, C., Dai, X. & Chai, W. Human Stn1 protects telomere integrity by promoting efficient lagging-strand synthesis at telomeres and mediating C-strand fill-in. *Cell Res.* **22**, 1681–1695 (2012). 22964711 10.1038/cr.2012.132 PMC3515754

[75] Flynn, R. L. et al. Alternative lengthening of telomeres renders cancer cells hypersensitive to ATR inhibitors. *Science* **347**, 273–277 (2015). 25593184 10.1126/science.1257216 PMC4358324

[76] van Schendel, R., Romeijn, R., Buijs, H. & Tijsterman, M. Preservation of lagging strand integrity at sites of stalled replication by Pol alpha-primase and 9-1-1 complex. *Sci. Adv.* **7**, eabf2278 (2021). 34138739 10.1126/sciadv.abf2278 PMC8133754

[77] Goncalves, T. et al. Phosphorylation of ‘SDT-like’ motifs in ATRX mediates its interaction with the MRN complex and is important for ALT pathway suppression. *Open Biol.* **14**, 240205 (2024). 39657822 10.1098/rsob.240205 PMC11631451

[78] Panda, D., Fernandez, D. J., Lal, M., Buehler, E. & Moss, B. Triad of human cellular proteins, IRF2, FAM111A, and RFC3, restrict replication of orthopoxvirus SPI-1 host-range mutants. *Proc. Natl Acad. Sci. USA* **114**, 3720–3725 (2017). 28320935 10.1073/pnas.1700678114 PMC5389286

[79] Nie, M. et al. FAM111A induces nuclear dysfunction in disease and viral restriction. *EMBO Rep.* **22**, e50803 (2021). 33369867 10.15252/embr.202050803 PMC7857424

[80] Palani, S. et al. Dimerization-dependent serine protease activity of FAM111A prevents replication fork stalling at topoisomerase 1 cleavage complexes. *Nat. Commun.* **15**, 2064 (2024). 38453899 10.1038/s41467-024-46207-w PMC10920703

[81] Hewitt, G. et al. Defective ALC1 nucleosome remodeling confers PARPi sensitization and synthetic lethality with HRD. *Mol. Cell.* **81**, 767–783 (2021). 33333017 10.1016/j.molcel.2020.12.006 PMC7895907

[82] Stanage, T. H. et al. RPA exhaustion activates SLFN11 to eliminate cells with heightened replication stress. *Nat. Cell Biol.* **28**, 240–254 (2026). 41514018 10.1038/s41556-025-01852-1 PMC12904793

[83] Doench, J. G. et al. Optimized sgRNA design to maximize activity and minimize off-target effects of CRISPR–Cas9. *Nat. Biotechnol.* **34**, 184–191 (2016). 26780180 10.1038/nbt.3437 PMC4744125

[84] Li, H. et al. The Sequence Alignment/Map format and SAMtools. *Bioinformatics* **25**, 2078–2079 (2009). 19505943 10.1093/bioinformatics/btp352 PMC2723002

[85] Li, W. et al. MAGeCK enables robust identification of essential genes from genome-scale CRISPR/Cas9 knockout screens. *Genome Biol.* **15**, 554 (2014). 25476604 10.1186/s13059-014-0554-4 PMC4290824

[86] Henson, J. D. et al. The C-Circle assay for alternative-lengthening-of-telomeres activity. *Methods* **114**, 74–84 (2017). 27595911 10.1016/j.ymeth.2016.08.016

[87] Idilli, A. I., Segura-Bayona, S., Lippert, T. P. & Boulton, S. J. A C-circle assay for detection of alternative lengthening of telomere activity in FFPE tissue. *STAR Protoc.* **2**, 100569 (2021). 34136834 10.1016/j.xpro.2021.100569 PMC8182116

