# Supplementary material for: Distinct ATRX functions cooperate with 9-1-1 and CST complexes to safeguard replication and telomere integrity
Source: Nat Struct Mol Biol. 2026 Jun 30;33(7):1037–50. doi: 10.1038/s41594-026-01827-2 (PMC13372662; doi:10.1038/s41594-026-01827-2)
Supplement: Supplementary file 1 — Reporting Summary [file 41594_2026_1827_MOESM1_ESM.pdf]

nature portfolio

Corresponding author(s): Simon J. Boulton

Last updated by author(s): 2026.05.10

Reporting Summary

Nature Portfolio wishes to improve the reproducibility of the work that we publish. This form provides structure for consistency and transparency in reporting. For further information on Nature Portfolio policies, see our Editorial Policies and the Editorial Policy Checklist.

Statistics

For all statistical analyses, confirm that the following items are present in the figure legend, table legend, main text, or Methods section.

n/a	Confirmed	
☐	☒	The exact sample size (*n*) for each experimental group/condition, given as a discrete number and unit of measurement
☐	☒	A statement on whether measurements were taken from distinct samples or whether the same sample was measured repeatedly
☐	☒	The statistical test(s) used AND whether they are one- or two-sided<br>*Only common tests should be described solely by name; describe more complex techniques in the Methods section.*
☒	☐	A description of all covariates tested
☒	☐	A description of any assumptions or corrections, such as tests of normality and adjustment for multiple comparisons
☐	☒	A full description of the statistical parameters including central tendency (e.g. means) or other basic estimates (e.g. regression coefficient) AND variation (e.g. standard deviation) or associated estimates of uncertainty (e.g. confidence intervals)
☐	☒	For null hypothesis testing, the test statistic (e.g. *F*, *t*, *r*) with confidence intervals, effect sizes, degrees of freedom and *P* value noted<br>*Give P values as exact values whenever suitable.*
☒	☐	For Bayesian analysis, information on the choice of priors and Markov chain Monte Carlo settings
☒	☐	For hierarchical and complex designs, identification of the appropriate level for tests and full reporting of outcomes
☒	☐	Estimates of effect sizes (e.g. Cohen's *d*, Pearson's *r*), indicating how they were calculated

Our web collection on statistics for biologists contains articles on many of the points above.

Software and code

Policy information about availability of computer code

Data collection	Chemidoc MP Image Lab Touch Software, Bio-Rad (version 3.0.1.14) for chemiluminescence imaging, Clariostar BMG Labtech (version 5.20 R5) for luminescence readings, BD FACSDiva software (v10) was used with BD Biosciences LSRFortessa analyser for flow cytometry data acquisition, Oxford Optronix Gelcount v1.2 for colony formation assays, Opera Phenix Plus High-Content Screening System (PerkinElmer) for native FISH image acquisition, Incucyte S3 software for live cell imaging acquisition and analysis.
Data analysis	CLARIOstar MARS Data analysis software (BML Labtech version 3.10), FlowJo version 10 for flow cytometry data analysis, GelCount (Oxford Optronics) for colony survival analysis, Harmony v5.1 High-Content Imaging and Analysis Software (PerkinElmer) for Opera Phenix image analysis, MaGeCK v0.5.9 analysis (Li. et al, 2014 Genome Biology). Graphs and numerical data (including statistics/error bars) were analyzed and plotted by Prism (GraphPad, Version 10.6) or in R Studio (R version 4.2.3 and ggplot2 package).

For manuscripts utilizing custom algorithms or software that are central to the research but not yet described in published literature, software must be made available to editors and reviewers. We strongly encourage code deposition in a community repository (e.g. GitHub). See the Nature Portfolio guidelines for submitting code & software for further information.

Data

Policy information about availability of data

All manuscripts must include a data availability statement. This statement should provide the following information, where applicable:

- Accession codes, unique identifiers, or web links for publicly available datasets
- A description of any restrictions on data availability
- For clinical datasets or third party data, please ensure that the statement adheres to our policy

Sequencing read raw counts from CRISPR screens generated in this study have been deposited at Mendeley data [DOI 10.17632/4wyg87kxrr.1]. Raw proteomics data have been deposited in the PRIDE repository under accession code PXD068487. The code used for image analysis of nuclear foci and colocalization in IF-FISH assays has been deposited at GitHub [DOI 10.5281/zenodo.10069688]. Source Data are provided with this paper. Any requests for resources or reagents should be directed to and will be fulfilled by the corresponding author.

Research involving human participants, their data, or biological material

Policy information about studies with human participants or human data. See also policy information about sex, gender (identity/presentation), and sexual orientation and race, ethnicity and racism.

Reporting on sex and gender	N/A
Reporting on race, ethnicity, or other socially relevant groupings	N/A
Population characteristics	N/A
Recruitment	N/A
Ethics oversight	N/A

Note that full information on the approval of the study protocol must also be provided in the manuscript.

Field-specific reporting

Please select the one below that is the best fit for your research. If you are not sure, read the appropriate sections before making your selection.

☒ Life sciences ☐ Behavioural & social sciences ☐ Ecological, evolutionary & environmental sciences

For a reference copy of the document with all sections, see nature.com/documents/nr-reporting-summary-flat.pdf

Life sciences study design

All studies must disclose on these points even when the disclosure is negative.

Sample size	No pre-determined sample sizes were utilised in this study. Experiments were always performed in biological duplicate (at a minimum). Sample sizes in immunofluorescence-based experiments were not pre-determined but were determined by capturing a set number of fields of view across multiple biological replicates.
Data exclusions	No data were excluded in these studies
Replication	All experiments were performed in at least biological duplicate at a minimum. No replicates were excluded in downstream analyses and all data were therefore reported. No issues arose with replication of experiments.
Randomization	No experiments were performed on organisms or participants. Randomization of samples was not relevant in this study as all experiments were conducted in an unbiased manner. Immunofluorescence-based experiments were performed using automated image acquisition as described in the materials and methods and thus was inherently unbiased.
Blinding	Blinding is not relevant to this study as microscopy images were taken and analyzed in an unbiased manner.<br>For metaphase spreads, samples were coded/numbered by researcher 1 (SSB) and processed and analyzed by researcher 2 (TT) without knowing genotype for each sample.

Reporting for specific materials, systems and methods

We require information from authors about some types of materials, experimental systems and methods used in many studies. Here, indicate whether each material, system or method listed is relevant to your study. If you are not sure if a list item applies to your research, read the appropriate section before selecting a response.

Materials & experimental systems

n/a	Involved in the study	
☐	☒	Antibodies
☐	☒	Eukaryotic cell lines
☒	☐	Palaeontology and archaeology
☒	☐	Animals and other organisms
☒	☐	Clinical data
☒	☐	Dual use research of concern
☒	☐	Plants

Methods

n/a	Involved in the study	
☒	☐	ChIP-seq
☐	☒	Flow cytometry
☒	☐	MRI-based neuroimaging

Antibodies

Antibodies used

Rabbit polyclonal anti-ATRX Bethyl Laboratories Cat# A301-045A, RRID:AB_2243144 (1:1000)
Rabbit monoclonal anti-ATRX (clone D1N2E) Cell Signaling Technology Cat# 14820, RRID:AB_2798630 (1:500)
Rabbit monoclonal anti-CASPASE-3 cleaved D175 (clone 5A1E) Cell Signaling Technology Cat# 9664, RRID:AB_2070042 (1:500)
Mouse monoclonal anti-CHK1 Cell Signaling Technology Cat# 2360, RRID:AB_2080320 (1:1000)
Rabbit monoclonal anti-CHK1 pS345 (clone 133D3)Cell Signaling Technology Cat# 2348, RRID:AB_331212 (1:1000)
Rabbit polyclonal anti-DAXX Santa Cruz Biotechnology Cat# sc-7152, RRID:AB_2088784 (1:500)
Rabbit monoclonal anti-FAM111A (clone EPR14407) Abcam Cat# ab184572, RRID:AB_3096019 (1:1000)
Rat monoclonal anti-HA tag (clone 3F10) Roche Cat# 11867423001, RRID:AB_390918 (1:2000)
Rabbit polyclonal anti-H2AX pS139 (gH2AX) Cell Signaling Technology Cat# 2577, RRID:AB_2118010 (1:1000, for Western)
Mouse monoclonal anti-H2AX pS139 (gH2AX) Millipore Cat# 05-636, RRID:AB_309864 (1:1000, for IF)
Mouse monoclonal anti-Histone H3 Abcam Cat# ab10799, RRID:AB_470239 (1:1000)
Rabbit monoclonal anti-HP1 alpha (clone EPR5777) Abcam Cat#ab109028, RRID:AB_10858495 (1:1000)
Rabbit polyclonal anti-HUS1 Abcam Cat# ab96297, RRID:AB_10680568 (1:500)
Rabbit polyclonal anti-KAP1 pS824 Bethyl Laboratories Cat# A300-767A, RRID:AB_669740 (1:1000)
Mouse monoclonal anti-KAP1 (clone 20C1) Abcam Cat# ab22553, RRID:AB_447151 (1:5000)
Rabbit monoclonal anti-MCM7 (clone EP1974Y) Abcam Cat# ab52489, RRID:AB_881187 (1:1000)
Mouse polyclonal anti-OBFC1/STN1 Abcam Cat# ab89250, RRID:AB_2156016 (1:1000)
Rabbit polyclonal anti-PARP & cleaved PARP Cell Signaling Technology Cat# 9542, RRID:AB_2160739 (1:1000)
Mouse monoclonal anti-PCNA (clone PC10) Santa Cruz Biotechnology Cat# sc-56, RRID:AB_628110 (1:1000)
Rabbit polyclonal anti-PML Bethyl Laboratories Cat# A301-167A, RRID:AB_873108 (1:1000, for Western)
Mouse monoclonal anti-PML (clone PG-M3) Santa Cruz Biotechnology Cat#sc-966, RRID:AB_628162 (1:500, for IF)
Rabbit polyclonal anti-RAD1 Novus Biologicals Cat# NBP2-13196 (1:1000)
Goat polyclonal anti-RAD17 Abcam Cat# ab2847, RRID:AB_2176307 (1:2000)
Mouse monoclonal anti-RAD9 Novus Biologicals Cat# NB120-13600, RRID:AB_791820 (1:500)
Mouse monoclonal anti-RPA (clone 9H8) Abcam Cat# ab2175, RRID:AB_302873 (1:1000)
Rabbit polyclonal anti-RPA pS33 Bethyl Laboratories Cat# A300-246A, RRID:AB_2180847 (1:500)
Rabbit polyclonal anti-RPA pS4/8 Bethyl Laboratories Cat# A300-245A, RRID:AB_210547 (1:500)
Mouse monoclonal anti-alpha-Tubulin Sigma-Aldrich Cat# T6074, RRID:AB_477582 (1:20000)
Mouse monoclonal anti-Vinculin Abcam Cat# ab11194, RRID:AB_297835 (1:10000)
Sheep polyclonal anti-Digoxigenin-AP, Fab fragments Roche Cat# 11093274910, RRID:AB_2734716 (1:10000)
Rat monoclonal anti-BrdU [BU1/75 (ICR1)] Abcam Cat# ab6326, RRID:AB_305426
Mouse monoclonal anti-BrdU (clone B44) BD Biosciences Cat# B44, RRID:AB_2313824
AbFlex® Histone H3.3 antibody (rAb) Active Motif, Cat#91191, RRID:AB_2793796
Goat polyclonal anti-mouse Immunoglobulins/HRP Agilent-Dako Cat# P0447, RRID:AB_2617137
Swine polyclonal anti-rabbit Immunoglobulins/HRP Agilent-Dako Cat# P0399, RRID:AB_2617141
Goat polyclonal anti-rat IgG Immunoglobulins/HRP Abcam Cat# ab97057, RRID:AB_10680316
Horse anti-mouse IgG, rat adsorbed (H+L), biotinylated Vector Laboratories Cat# BA-2001, RRID:AB_2336180
Goat anti-mouse IgG (H+L), Alexa Fluor 488 Thermo Fisher Scientific Cat# A11001, RRID:AB_2534069
Goat anti-rabbit IgG (H+L), Alexa Fluor 488 Thermo Fisher Scientific Cat# A11008, RRID:AB_143165
Goat anti-rabbit IgG (H+L), Alexa Fluor 546 Thermo Fisher Scientific Cat# A11010, RRID:AB_2534077
Goat anti-rat IgG (H+L), Alexa Fluor 555 Thermo Fisher Scientific Cat# A-21434, RRID:AB_2535855

Validation

Key antibodies used in this study (ATRX, DAXX, 9-1-1/RAD17, STN1, FAM111A, PML) were confirmed in genetic knockouts, which showed loss of the corresponding band in western blot. Antibodies against common DDR markers (KAP1, RPA, H2AX) and apoptotic makers (PARP, Caspase 3) have been used extensively and validated in the literature, from the supplier's catalog or by using the CiteAb database.

Eukaryotic cell lines

Policy information about cell lines and Sex and Gender in Research

Cell line source(s)

No primary cell lines or cell lines derived from participants were utilised in this study. All commercially available cell lines in this study were sourced from the Cell Science facility at the Francis Crick Institute.

Human: eHAP iCas9 Hewitt et al. Mol Cell 81, 767-783.
Human: NCI-H460 iCas9 Stanage et al. Nat Cell Biol 28, 240–254.

Human: eHAP iCas9 ATRX-KO g3 This paper
Human: eHAP iCas9 ATRX-KO g3 + PB-empty This paper
Human: eHAP iCas9 ATRX-KO g3 + PB-ATRX-HA This paper
Human: eHAP iCas9 ATRX-KO g3 + PB-ATRX-LXVXLmut-HA This paper
Human: eHAP iCas9 ATRX-KO g3 + PB-ATRX-DBMmut-HA This paper
Human: eHAP iCas9 ATRX-KO g3 + PB-ATRX-PIPmut-HA This paper
Human: eHAP iCas9 ATRX-K1600R This paper
Human: eHAP iCas9 ATRX-KO g4 This paper
Human: eHAP iCas9 STN1-KO This paper
Human: eHAP iCas9 CTC1-KO This paper
Human: eHAP iCas9 TERT-KO This paper
Human: eHAP iCas9 FAM111A-KO c2 This paper
Human: eHAP iCas9 FAM111A-KO c6 This paper
Human: eHAP iCas9 DAXX-KO c1 This paper
Human: eHAP iCas9 DAXX-KO c2 This paper
Human: eHAP iCas9 ATRX-TERT-DKO This paper
Human: eHAP iCas9 ATRX-FAM111A-DKO c4 This paper
Human: eHAP iCas9 ATRX-FAM111A-DKO c9 This paper
Human: eHAP iCas9 ATRX-DAXX-DKO c1 This paper
Human: eHAP iCas9 ATRX-DAXX-DKO c2 This paper
Human: NCI-H460 iCas9 ATRX-KO This paper
Human: eHAP iCas9 lenti-sgRNA (Supp. Table 4) This paper
Human: eHAP iCas9 ATRX-KO g3 lenti-sgRNA (Supp. Table 4) This paper
Human: eHAP iCas9 FAM111A-KO c6 lenti-sgRNA (Supp. Table 4) This paper
Human: eHAP iCas9 ATRX-FAM111A-DKO c9 lenti-sgRNA (Supp. Table 4) This paper
Human: NCI-H460 iCas9 lenti-sgRNA (Supp. Table 4) This paper
Human: NCI-H460 iCas9 ATRX-KO lenti-sgRNA (Supp. Table 4) This paper

Authentication

Standard procedure in the Cell Science facility at the Francis Crick Institute is to authenticate cell lines by STR profiling. Cell lines generated in this study were also authenticated by Western Blotting and Sanger Sequencing of CRISPR/Cas9 cut site.

Mycoplasma contamination

Standard procedure in the Cell Science facility at the Francis Crick Institute is to confirm all cell lines are free of mycoplasma contamination - all cell lines in this study are mycoplasma negative.

Commonly misidentified lines
(See ICLAC register)

No commonly misidentified cell lines were used in this study.

Plants

Seed stocks

No plants were used in this study.

Novel plant genotypes

No plants were used in this study.

Authentication

No plants were used in this study.

Flow Cytometry

Plots

Confirm that:

- [x] The axis labels state the marker and fluorochrome used (e.g. CD4-FITC).
- [x] The axis scales are clearly visible. Include numbers along axes only for bottom left plot of group (a 'group' is an analysis of identical markers).
- [x] All plots are contour plots with outliers or pseudocolor plots.
- [x] A numerical value for number of cells or percentage (with statistics) is provided.

Methodology

Sample preparation

Cell lines were tested for Cas9 activity as described in the materials and methods section. Cells were trypsinised and diluted in PBS and run natively on the flow cytometers (for Cas9 activity) or fixed and stained as detailed in the materials and methods (cell cycle analysis/EdU).

Instrument

BD Biosciences LSRFortessa

Software	BD FACSDiva was used to collect data and FlowJo was used to analyse the data
Cell population abundance	For Cas9 cutting efficiency analysis, 20,000 cells were analysed as BFP+. For cell cycle analysis, 10,000 cells were analysed as 'Single cells'. No sorting of specific cell populations was included in flow cytometry experiments included within this manuscript.
Gating strategy	Cells were gated using Forward Scatter Area (FSC-A) vs Side Scatter Area (SSC-A) to remove debris. 'Single cells' were then gated from doublets/clumps using FSC-H vs FSC-A ratio and gating of objects with FSC-A:FSC-H ratio of roughly 1. For Cas9 cutting efficiency analysis, BFP+ population was gated (450-50-A vs FSC-A) and then the BFP+/GFP- population was gated with the use of a non-Dox control (450-50-A vs 530-30-A). For EdU and cell cycle analysis, 'Single cells' were gated with a gating strategy that is shown in Extended Data Figure 5b.

☒ Tick this box to confirm that a figure exemplifying the gating strategy is provided in the Supplementary Information.
