# Supplementary material for: Distinct ATRX functions cooperate with 9-1-1 and CST complexes to safeguard replication and telomere integrity
Source: Nat Struct Mol Biol. 2026 Jun 30;33(7):1037–50. doi: 10.1038/s41594-026-01827-2 (PMC13372662; doi:10.1038/s41594-026-01827-2)

Fig. 1a: ATRX

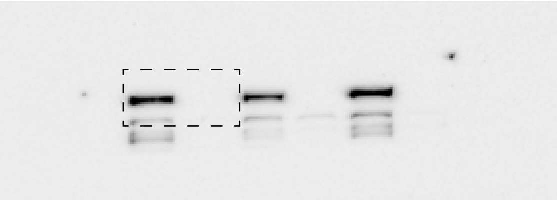

Fig. 1a: CHK1

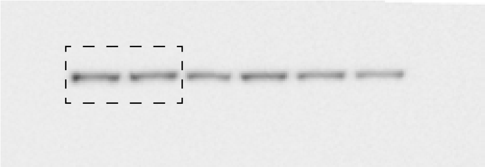

Fig. 1a: Ponc.

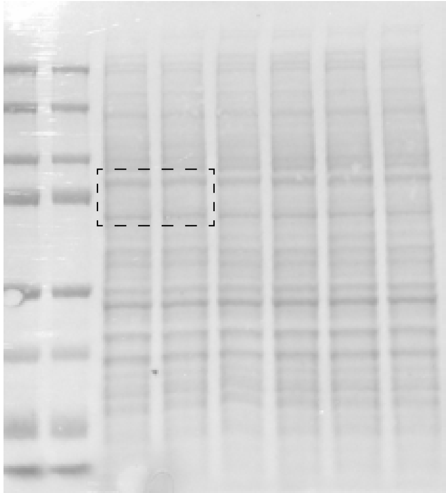

Fig. 2e: ATRX

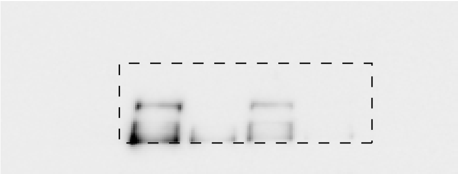

Fig. 2e: KAP1

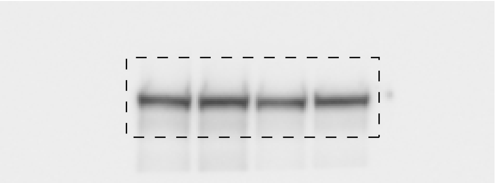

Fig. 2e: KAP1 pS824

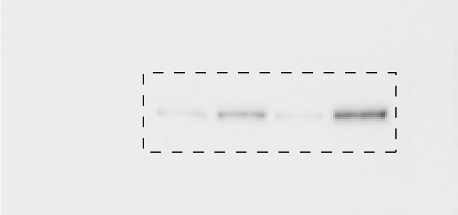

Fig. 2e: RPA32 pS4/8

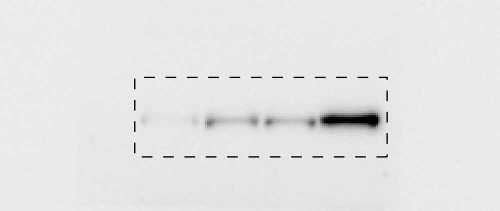

Fig. 2e: RPA32

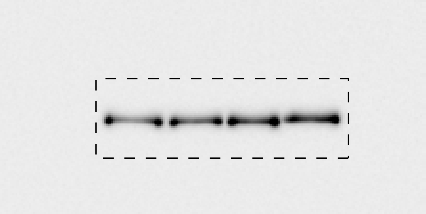

Fig. 2e: Vinculin

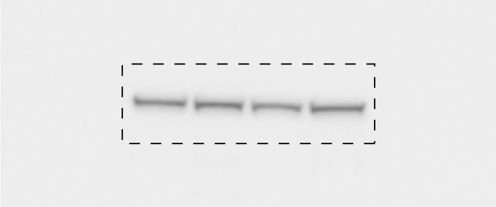

Fig. 2e: Ponc.

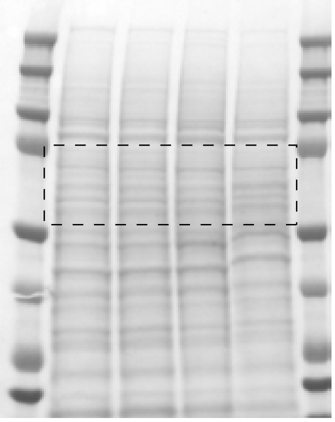

Fig. 3a: ATRX

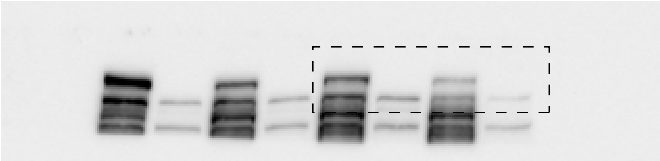

Fig. 3a: PARP

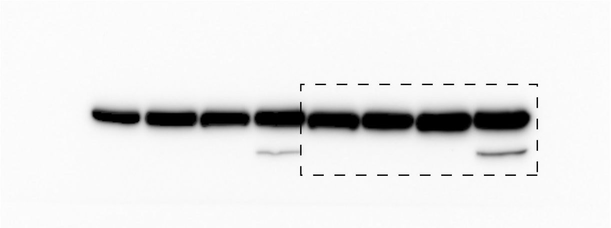

Fig. 3a: CHK1

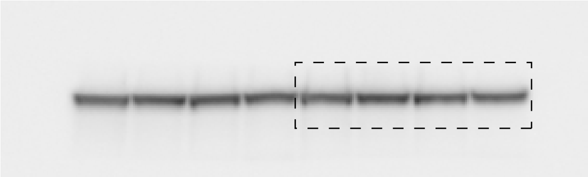

Fig. 3a: RPA32

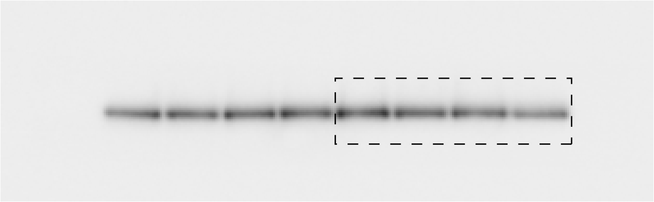

Fig. 3a: cleaved Caspase-3

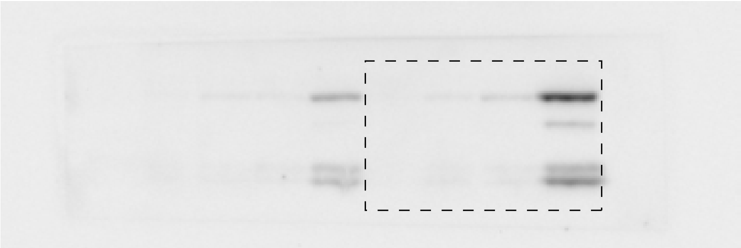

Fig. 3a: α-Tubulin

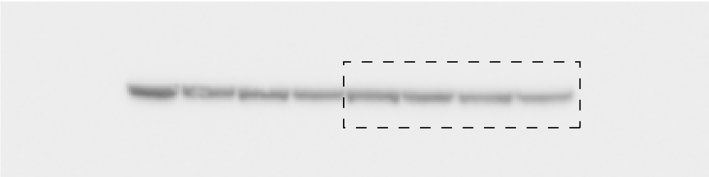

Fig. 3a: RAD17

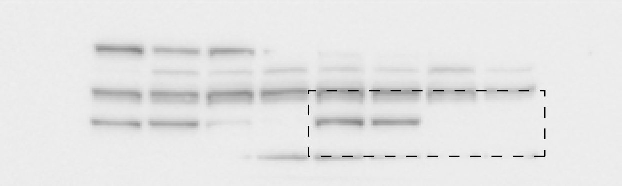

Fig. 3a: CHK1 pS345

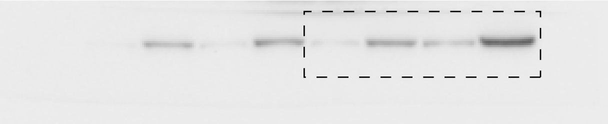

Fig. 3a: RPA32 pS4/8

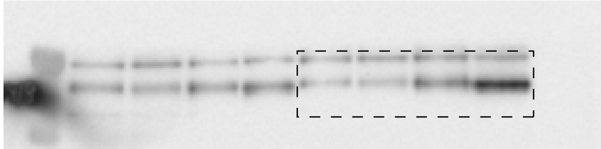

Fig. 3a: γH2AX

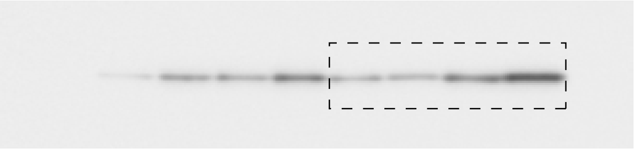

Fig. 3a: Ponc.

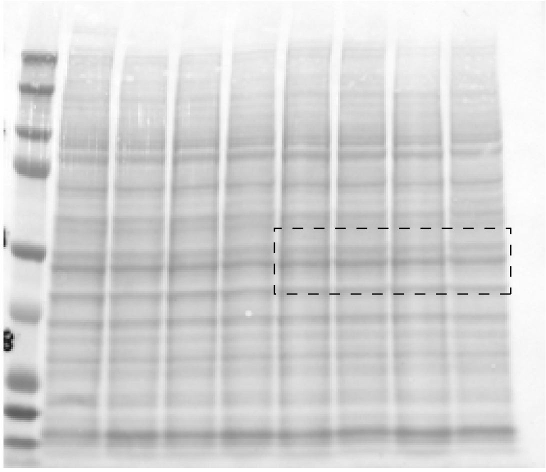

Fig. 4b: MCM7

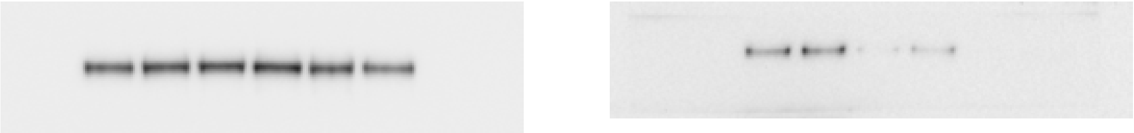

Fig. 4b: PCNA

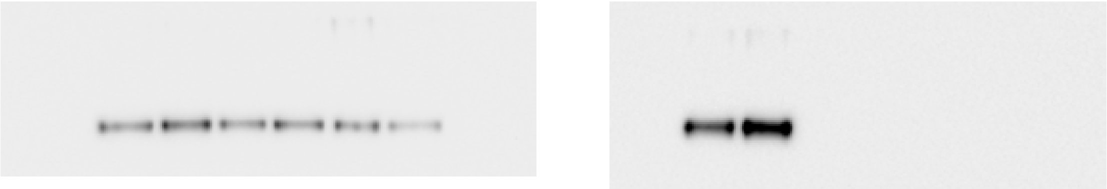

Fig. 4b: γH2AX

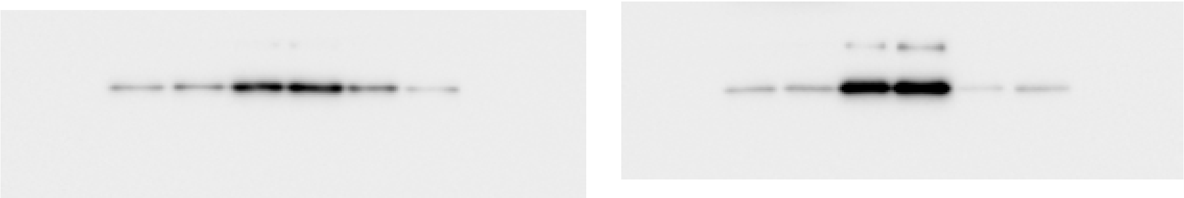

Fig. 4b: Histone H3

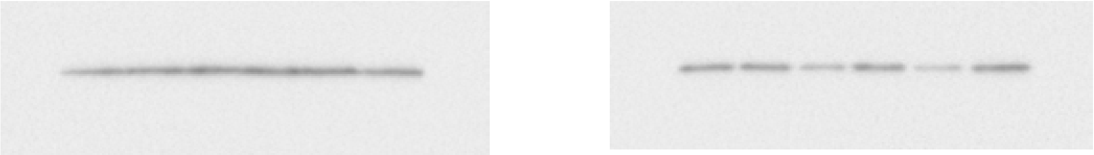

Fig. 4b: Ponc.

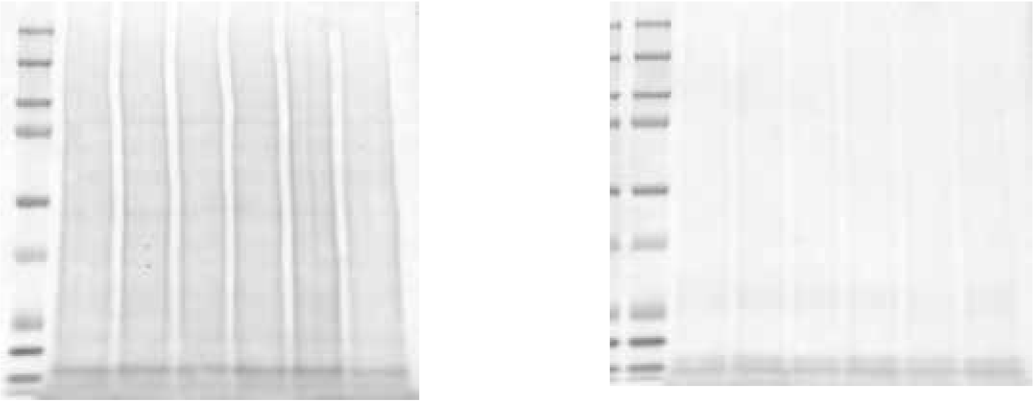

Fig. 4h: ATRX

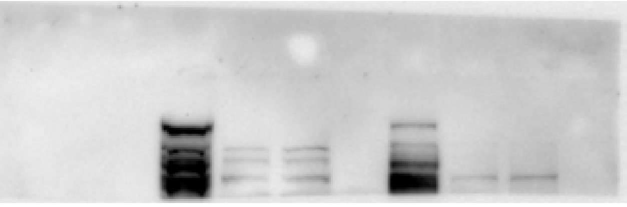

Fig. 4j: ATRX

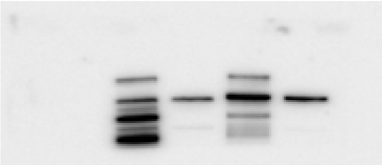

Fig. 4h: FAM111A

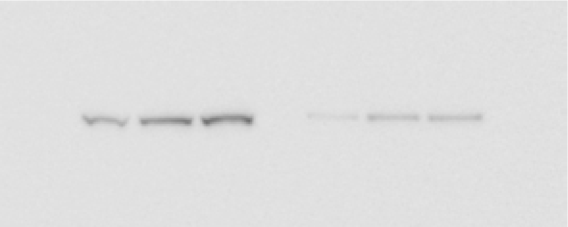

Fig. 4j: FAM111A

Fig. 4h: α-Tubulin

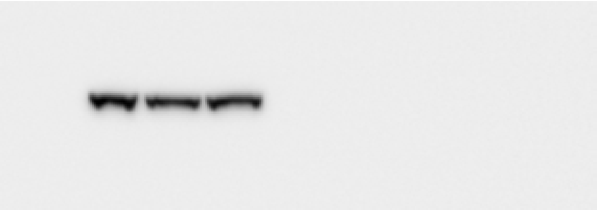

Fig. 4j: α-Tubulin

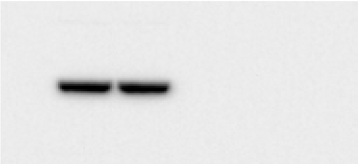

Fig. 4h: Histone H3

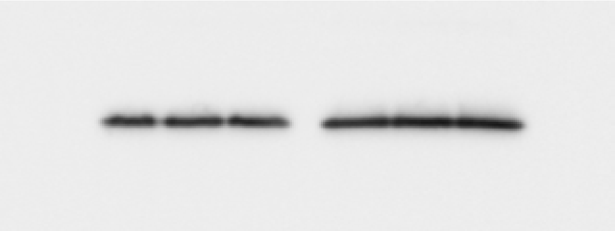

Fig. 4j: Histone H3

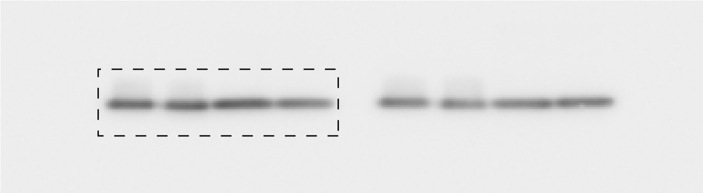

Fig. 4h: Ponc.

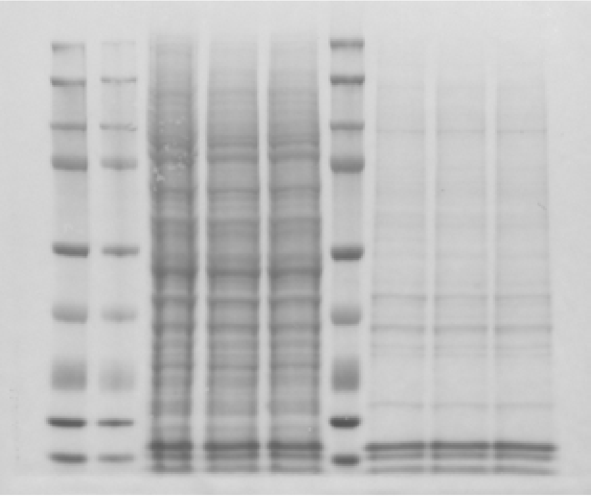

Fig. 4j: Ponc.

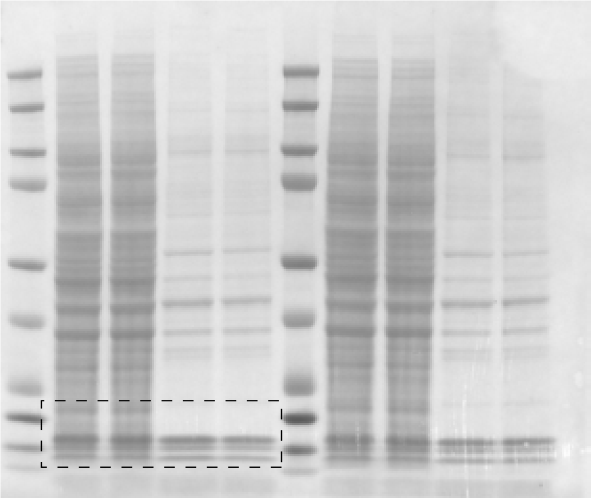

Fig. 5b: ATRX

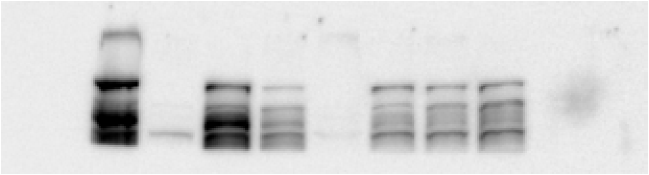

Fig. 5b: HA

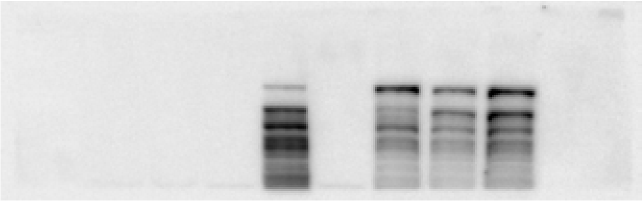

Fig. 5b: α-Tubulin (top)

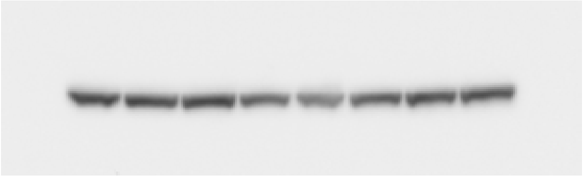

Fig. 5b: α-Tubulin (bottom)

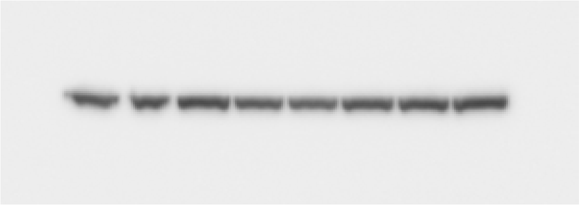

Fig. 5b: Ponc. (top)

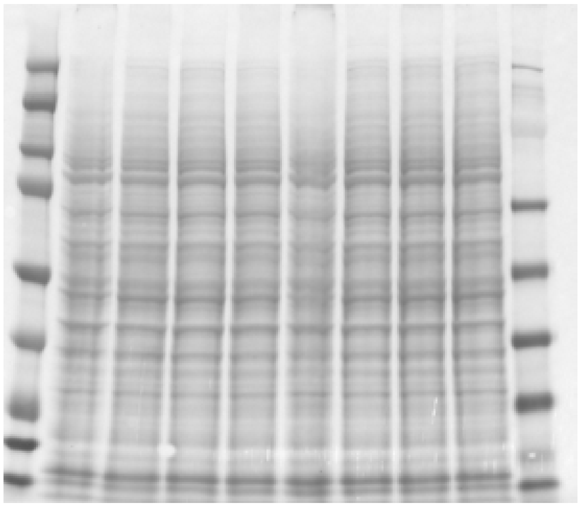

Fig. 5b: Ponc. (bottom)

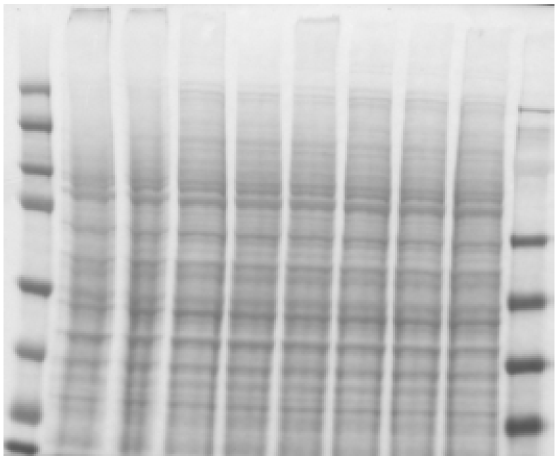

Fig. 5f: ATRX

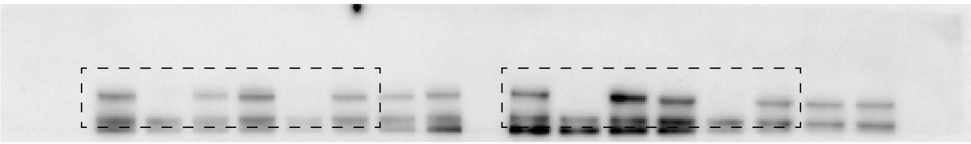

Fig. 5f: HA

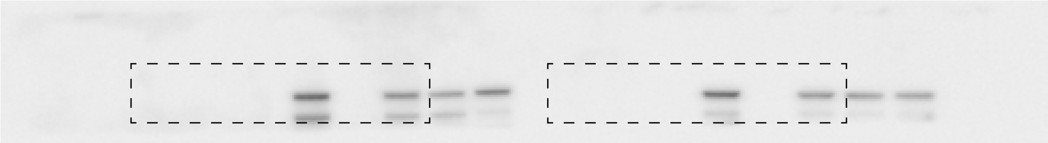

Fig. 5f: FAM111A

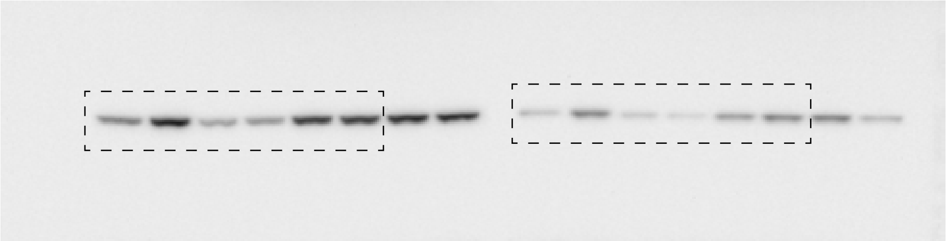

Fig. 5f: α-Tubulin

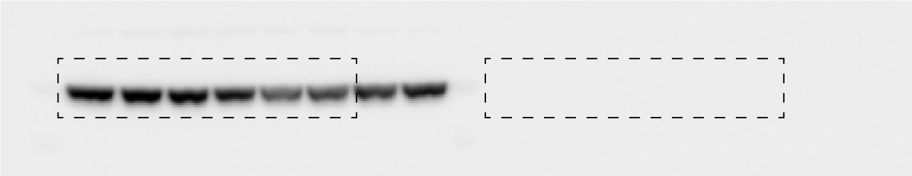

Fig. 5f: Histone H3

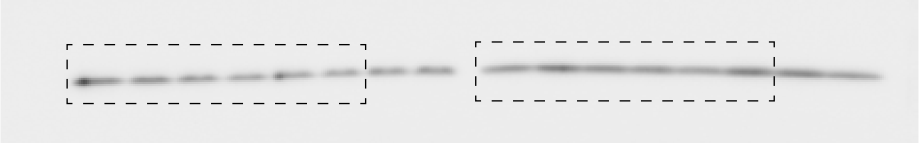

Fig. 5f: Ponc.

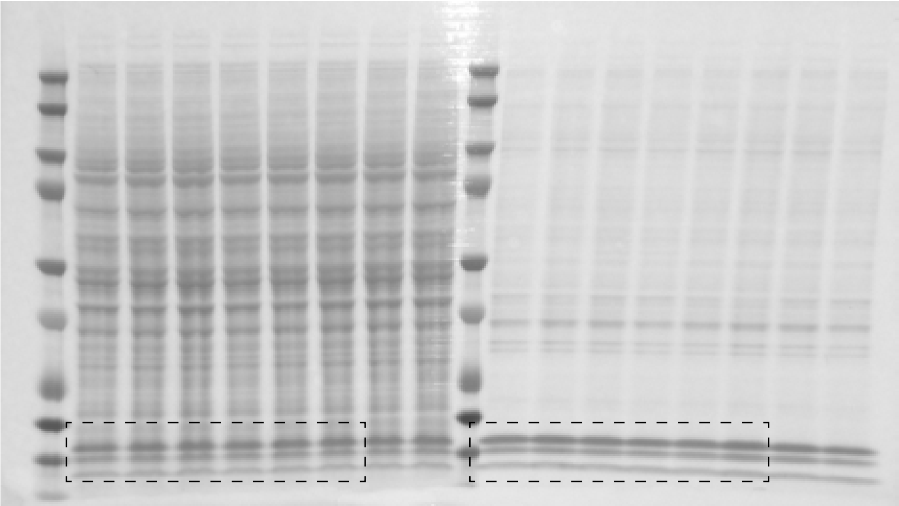

Supplement: Supplementary file 4 — Single file with clearly labeled unprocessed blots for each figure. [file 41594_2026_1827_MOESM4_ESM.pdf]
