# Supplementary material for: Distinct ATRX functions cooperate with 9-1-1 and CST complexes to safeguard replication and telomere integrity
Source: Nat Struct Mol Biol. 2026 Jun 30;33(7):1037–50. doi: 10.1038/s41594-026-01827-2 (PMC13372662; doi:10.1038/s41594-026-01827-2)

ED Fig. 1f: ATRX

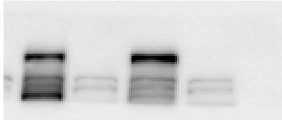

ED Fig. 1g: ATRX

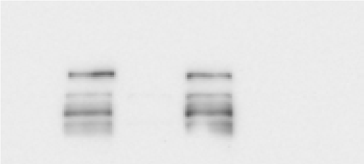

ED Fig. 1h: ATRX

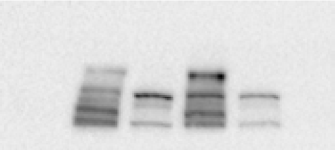

ED Fig. 1i: ATRX

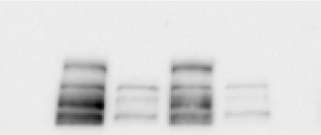

ED Fig. 1f: RAD9A

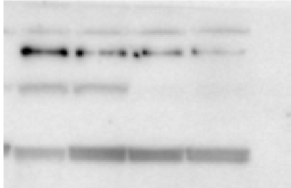

ED Fig. 1g: HUS1

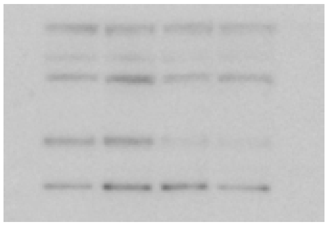

ED Fig. 1h: RAD1

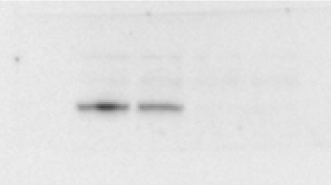

ED Fig. 1i: RAD17

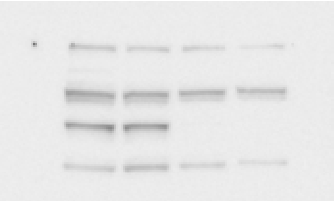

ED Fig. 1f: Ponc.

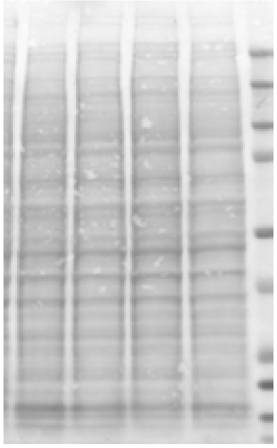

ED Fig. 1g: Ponc.

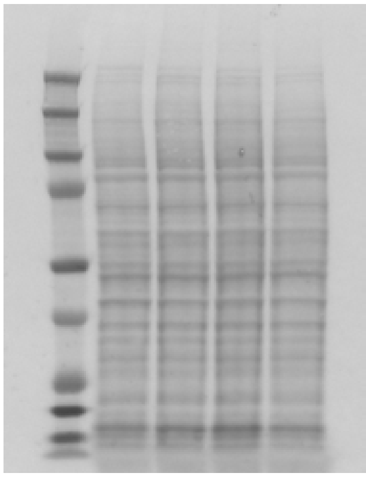

ED Fig. 1h: Ponc.

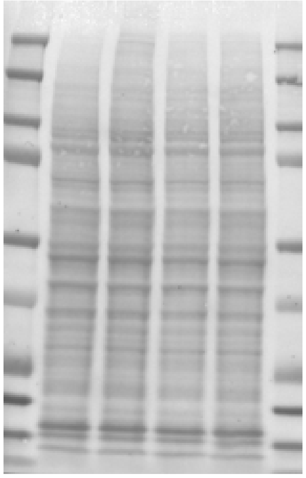

ED Fig. 1i: Ponc.

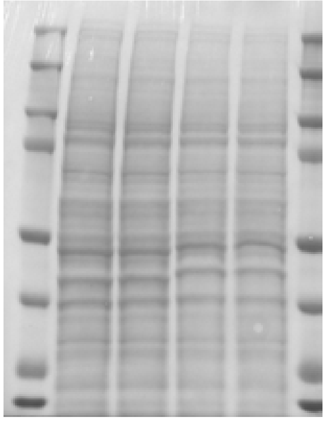

ED Fig. 2e: ATRX

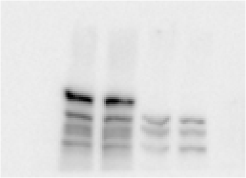

ED Fig. 2j: ATRX (left)

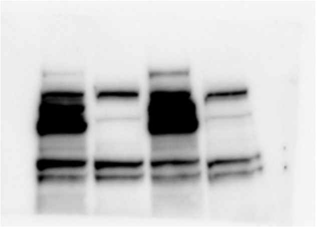

ED Fig. 2j: ATRX (right)

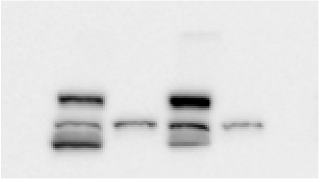

ED Fig. 2e: STN1

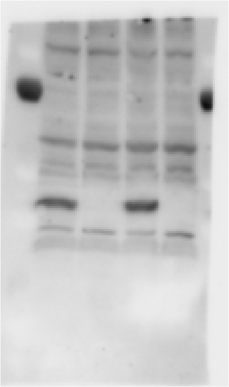

ED Fig. 2j: RAD1

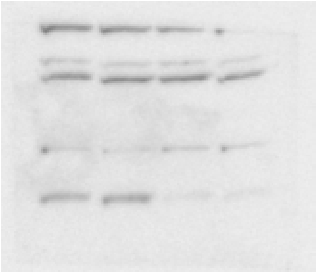

ED Fig. 2j: STN1

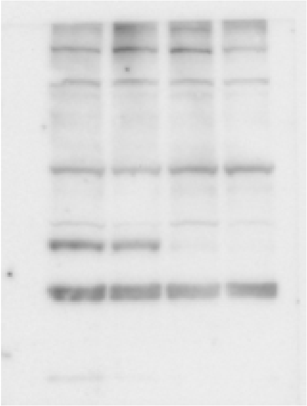

ED Fig. 2e: Ponc.

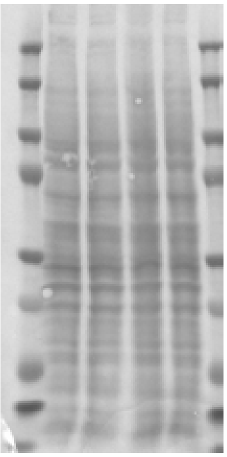

ED Fig. 2j: Ponc. (left)

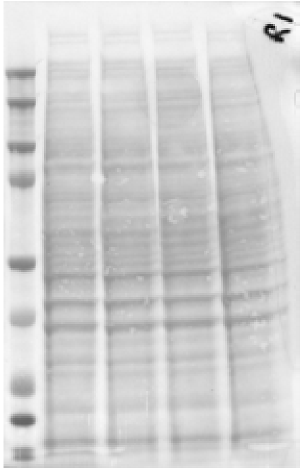

ED Fig. 2j: Ponc. (right)

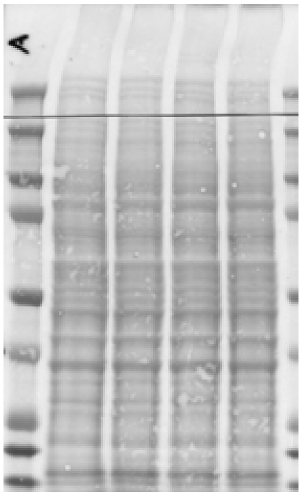

ED Fig. 3a: STN1

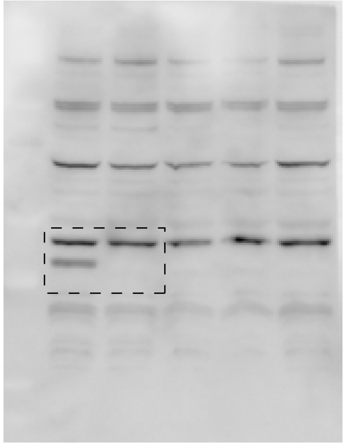

ED Fig. 3a: α-Tubulin

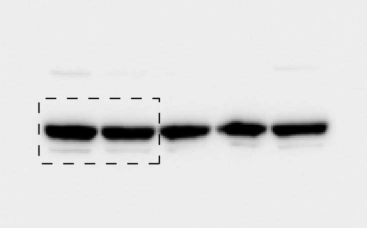

ED Fig. 4f: ATRX

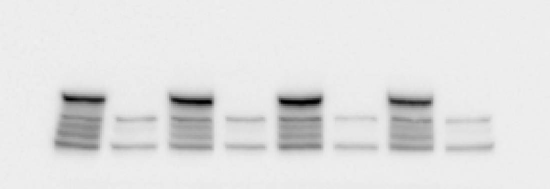

ED Fig. 4f: STN1

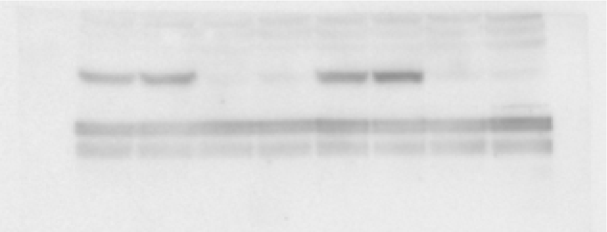

ED Fig. 4f: PML

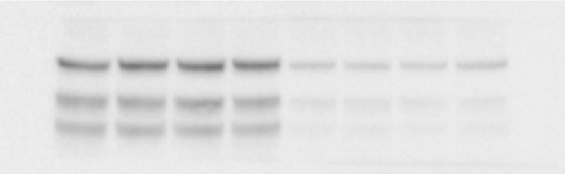

ED Fig. 3c: ATRX

ED Fig. 3c: STN1

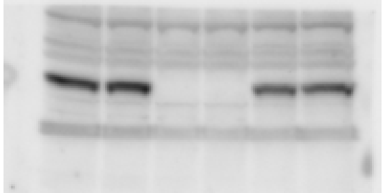

ED Fig. 3c: Vinculin

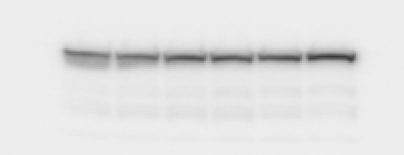

ED Fig. 3c: Ponc.

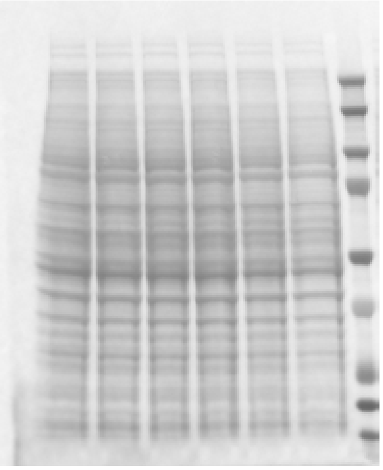

ED Fig. 4f: Ponc.

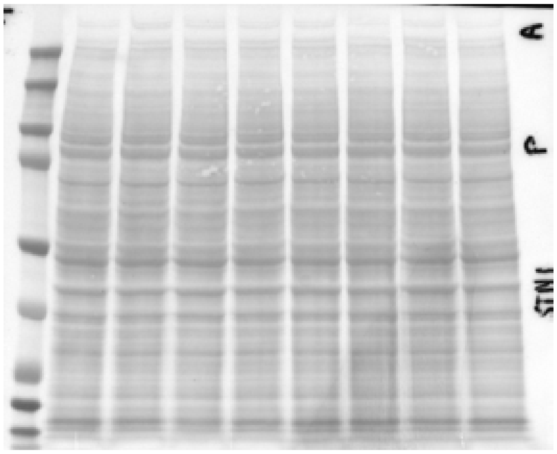

ED Fig. 5a: ATRX

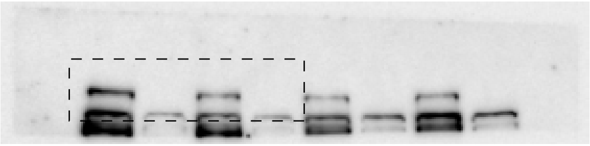

ED Fig. 5a: RAD1

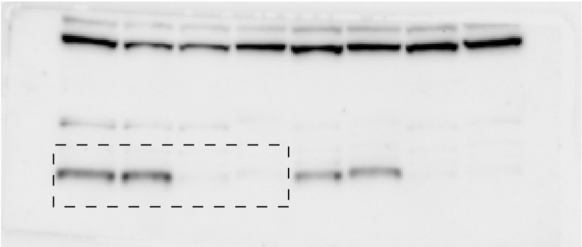

ED Fig. 5a: PARP

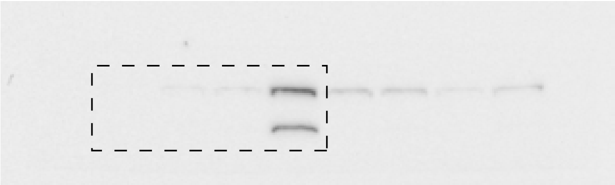

ED Fig. 5a: CHK1 pS345

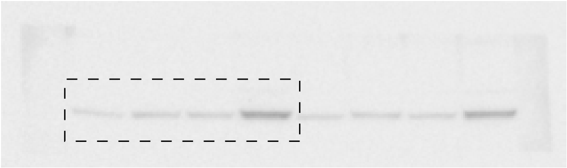

ED Fig. 5a: CHK1

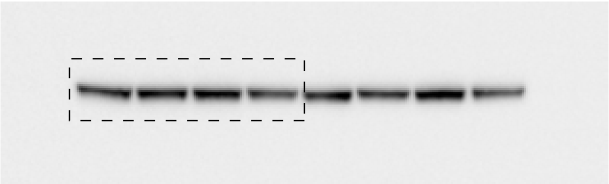

ED Fig. 5a: RPA32 pS4/8

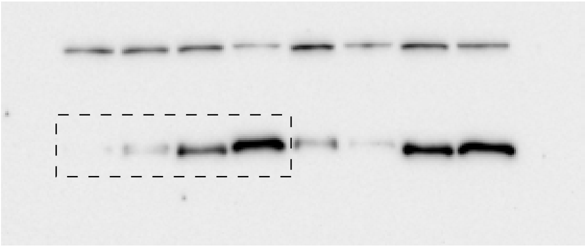

ED Fig. 5a: RPA32

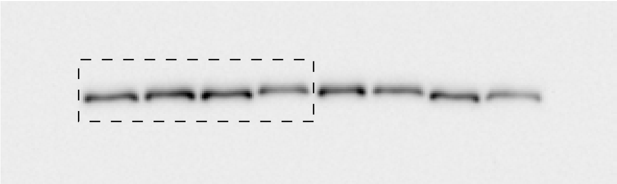

ED Fig. 5a: Vinculin

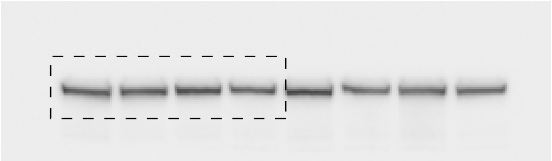

ED Fig. 5a: Ponc.

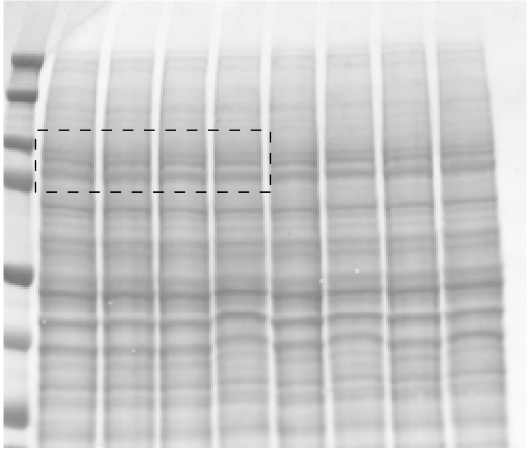

ED Fig. 5h: ATRX

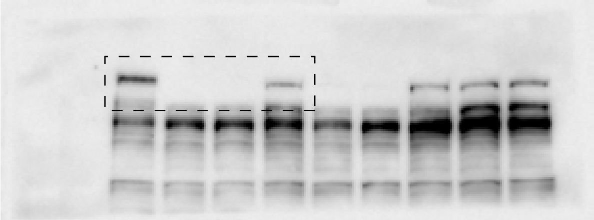

ED Fig. 5h: HA

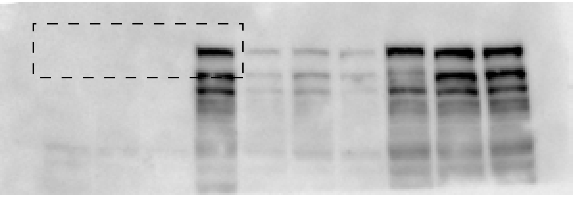

ED Fig. 5h: Ponc. (top)

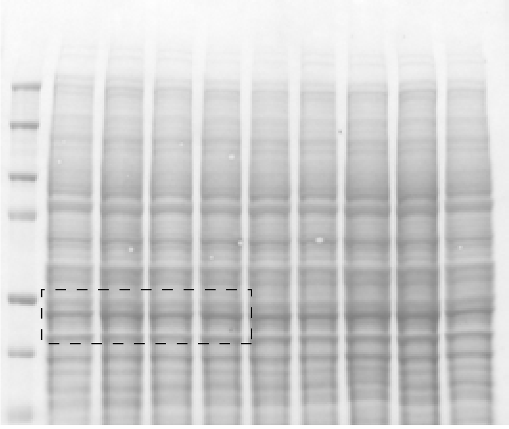

ED Fig. 5h: Ponc. (bottom)

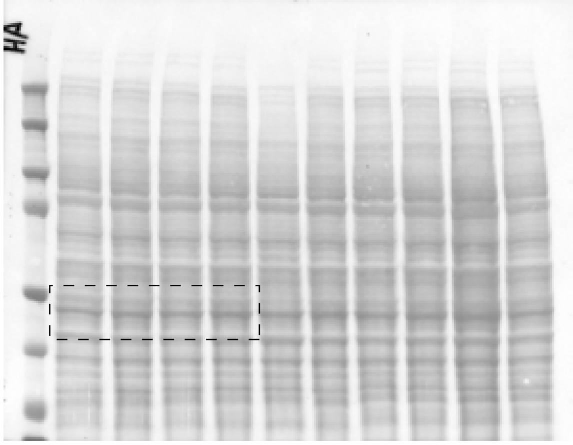

ED Fig. 6j: FAM111A

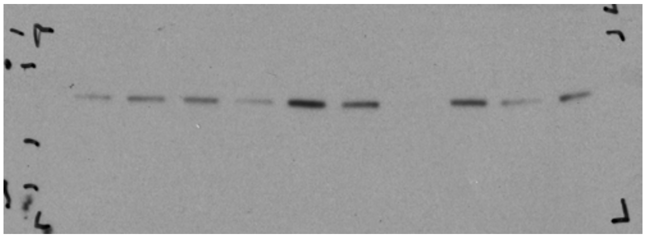

ED Fig. 6j: Histone H3

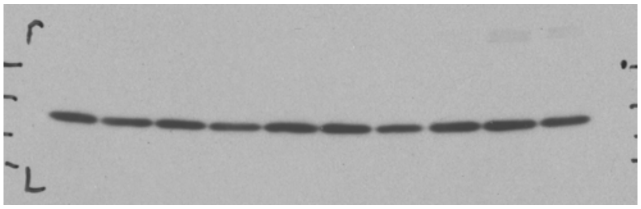

ED Fig. 7a: ATRX

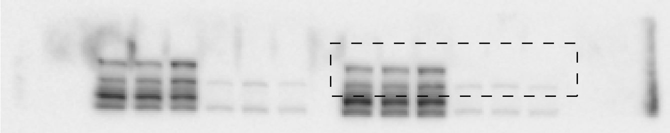

ED Fig. 7a: Ponc.

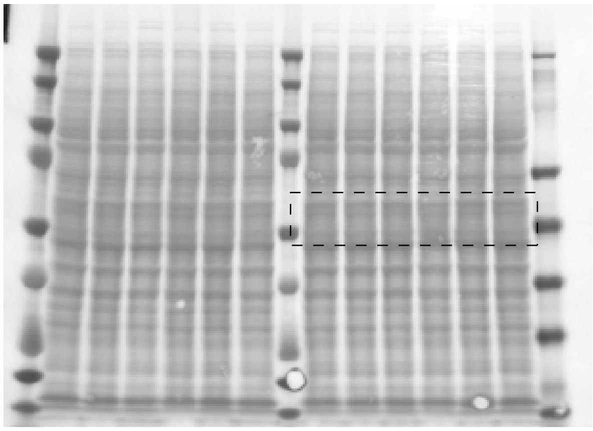

ED Fig. 7a: FAM111A

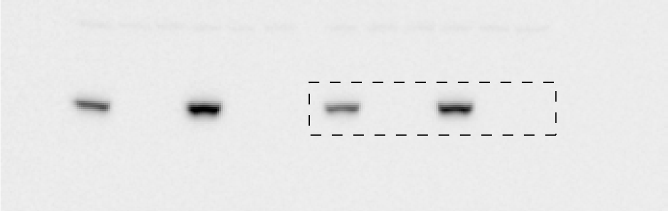

ED Fig. 7a: RPA32

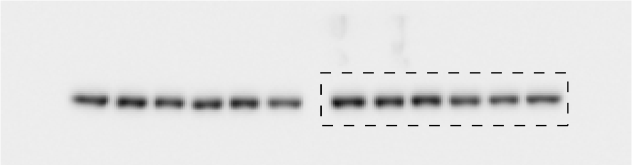

ED Fig. 7b: ATRX

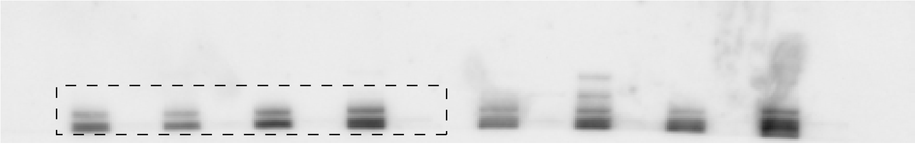

ED Fig. 7b: FAM111A

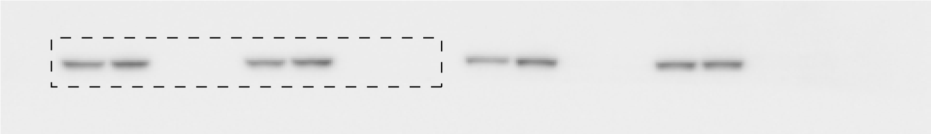

ED Fig. 7b: RPA32 pS4/8

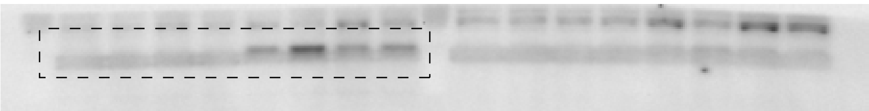

ED Fig. 7b: RPA32

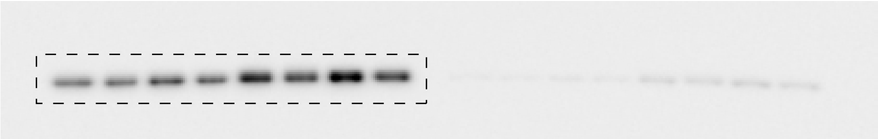

ED Fig. 7b: γH2AX

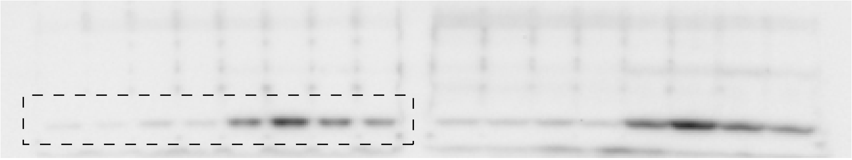

ED Fig. 7b: α-Tubulin

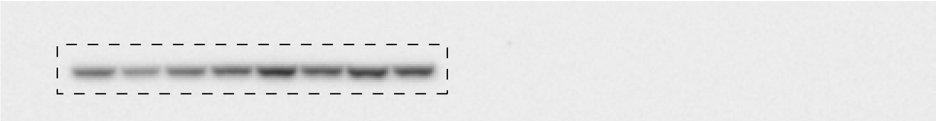

ED Fig. 7b: Ponc.

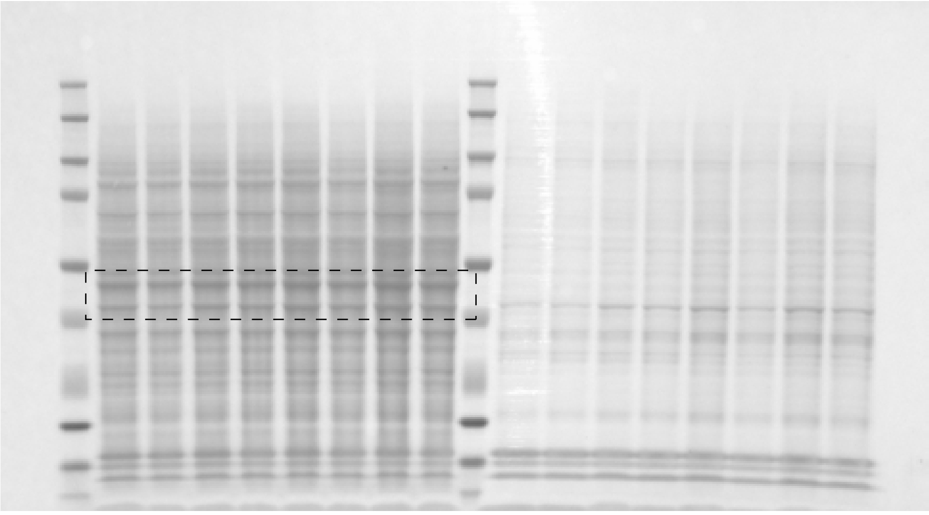

ED Fig. 7e: ATRX

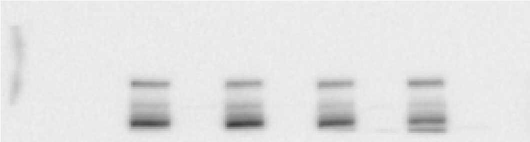

ED Fig. 7e: FAM111A

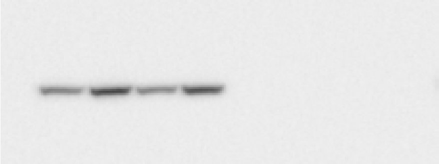

ED Fig. 7e: RAD1

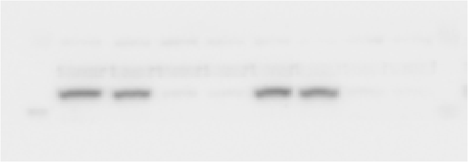

ED Fig. 7e: Ponc.

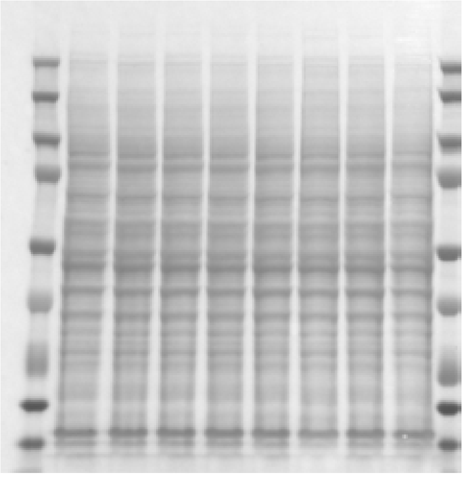

ED Fig. 8a: ATRX (input)

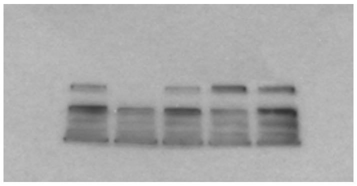

ED Fig. 8a: ATRX (IP)

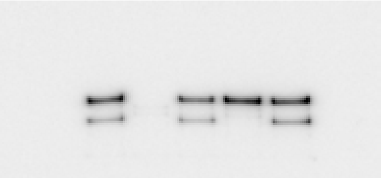

ED Fig. 8a: DAXX (input)

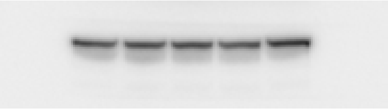

ED Fig. 8a: DAXX (IP)

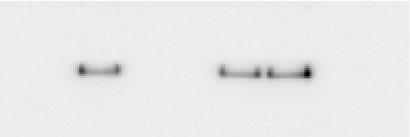

ED Fig. 8a: HP1α (input)

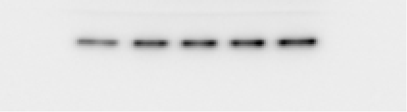

ED Fig. 8a: HP1α (IP)

ED Fig. 8g: HA

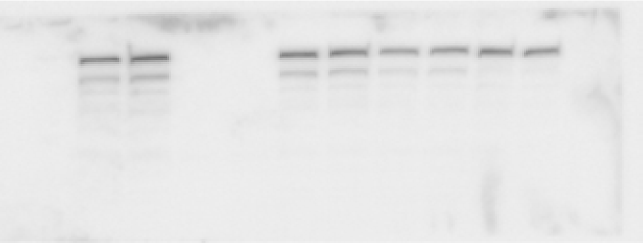

ED Fig. 8g: RAD1

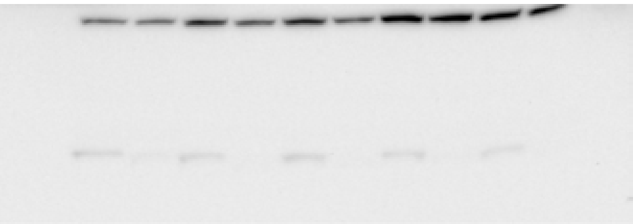

ED Fig. 8g: Ponc.

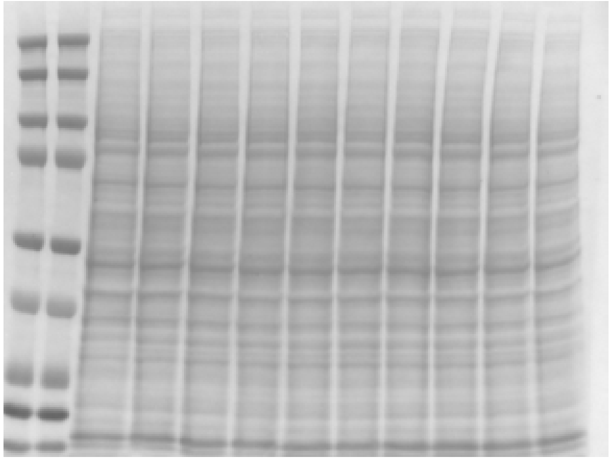

ED Fig. 9e: ATRX

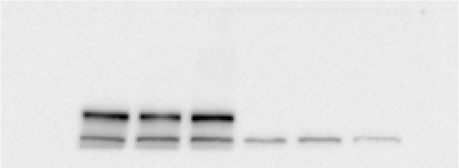

ED Fig. 9e: DAXX

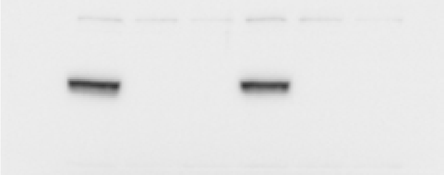

ED Fig. 9e: α-Tubulin

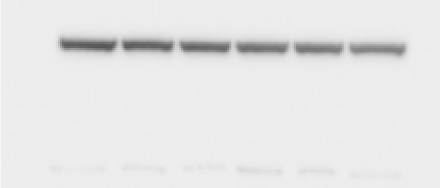

ED Fig. 9e: Ponc.

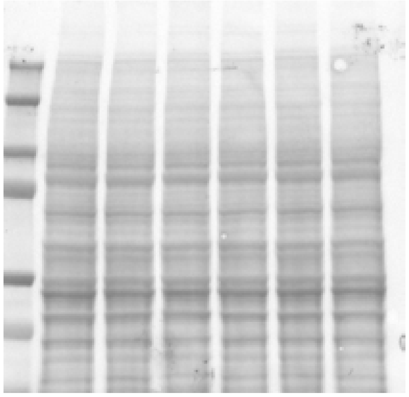

Supplement: Supplementary file 6 — Single file with clearly labeled unprocessed blots for each extended figure. [file 41594_2026_1827_MOESM6_ESM.pdf]
